# Differential expression pattern of CC chemokine receptor 7 guides precision treatment of hepatocellular carcinoma

**DOI:** 10.1038/s41392-025-02308-6

**Published:** 2025-07-21

**Authors:** Jie Qin, Qianyi Gong, Cheng Zhou, Jietian Xu, Yifei Cheng, Weiyue Xu, Di Zhu, Yiming Liu, Yuye Zhang, Yanru Wang, Lingling Gao, Lanfang Li, Wulei Hou, Qian Li, Binbin Liu, Yazhen Zhu, Zuoyun Wang, Jieyi Shi, Shuangjian Qiu, Chunmin Liang

**Affiliations:** 1 Lab of Tumor Immunology, Department of Human Anatomy, Histology & Embryology, School of Basic Medical Sciences, Fudan University, Shanghai, China; 2 Liver Cancer Institute, Zhongshan Hospital, Fudan University, and Key Laboratory of Carcinogenesis and Cancer Invasion of Ministry of Education, Shanghai, China; 3 Department of Colorectal Surgery and Colorectal Cancer Center, Zhongshan Hospital, Fudan University, Shanghai, China; 4 Department of Pathology and Laboratory Medicine, David Geffen School of Medicine at UCLA, Los Angeles, CA 90095 USA

**Keywords:** Cancer microenvironment, Medical research

## Abstract

The treatment of hepatocellular carcinoma (HCC) faces challenges of low response rates to targeted drugs and immune checkpoint inhibitors, which are influenced by complicated microenvironment of HCC. In this study, the complex tumor microenvironment was identified by using tissue microarray (TMA), spatial transcriptomes and single-cell sequencing. High expression of CC chemokine receptor 7 (CCR7) in tumor cells predicted lower Overall Survival (OS). Conversely, CRISPR-Cas9-mediated knockout of CCR7 enhanced the sensitivity of HCC to sorafenib in preclinical experiments, resulting from the inhibition of epithelial-mesenchymal transition through the AKT and ERK signaling pathways. Simultaneously, we revealed CCR7 expression in stromal cells, with increased infiltration of CCR7^+^ immune cells into the tumor mesenchyme associated with high CCL21 expression at tumor sites. Subsequently, VEGF-C was identified as an independent predictor of higher patient OS and showed a significant positive correlation with CCR7 signaling. Interestingly, exogenous VEGF-C was found to promote the formation of tertiary lymphoid structures (TLSs) by activating lymphatic angiogenesis and the CCL21/CCR7 axis. As a result, VEGF-C treatment enhanced the efficacy of anti-PD-1 immunotherapy. This study highlights the opposing effects of tumor cell-derived versus stromal cell-derived CCR7 expression and guides the precision treatment for HCC.

## Introduction

Hepatocellular carcinoma (HCC) incidence is increasing over the years and has become the leading cause of cancer-related deaths worldwide.^1^ A considerable proportion of patients with HCC are diagnosed at advanced stages and need effective systemic therapies. The current approved systemic therapies for advanced HCC include Sorafenib, Lenvatinib and nivolumab (anti-PD-1), etc.^2,3^ Sorafenib is a first-line treatment for advanced HCC and has been proven to provide clinical benefits for patients with advanced HCC.^4^ However, the emergence of reduced sensitivity has led to an increasing number of patients being unable to achieve long-term survival with sorafenib treatment.^5^ In HCC, sorafenib-insensitive tumor cells often exhibit distinct mesenchymal phenotypes.^6^ In cancer immunotherapy, immune checkpoint inhibitors (ICIs), particularly anti-PD-1 and anti-PD-L1, have emerged as effective therapeutic options.^7^ However, only a small subset of HCC patients respond to the immunotherapy,^3^ which is mainly determined by the tumor microenvironment (TME), such as heterogeneity in HCC resulting in different responses to immunotherapy.^8^ Previous studies have reported that within the TME, a high density of infiltrating lymphocytes, increased inflammatory immune infiltration, low infiltration of immunosuppressive cells, and the presence of tertiary lymphoid structures (TLSs) can predict a favorable response to immunotherapy.^9–11^ In brief, although these treatments have conveyed effectiveness, the improvement in overall survival (OS) remains insignificant, potentially owning to heterogeneous TME and leading to primary or acquired resistance to treatment.^12–14^ Obviously, there is an urgent need to explain the specific mechanisms by which tumor cells elicit resistance to these therapies, in order to refine these regimens and stratify patients who may benefit from them.

Chemokines are chemotactic cytokines that not only regulate the migration of immune cells, but also regulate the phenotype and function of immune cells by regulating their localization and cellular interactions in lymphoid tissues and the TME.^15^ CCR7 (CC chemokine receptor 7) is a well-known and G-protein-coupled transmembrane protein with two high-affinity ligands, CC motif chemokine 21 (CCL21) and 19 (CCL19). In physiological conditions, CCR7 is predominantly expressed in various T cell populations and mature dendritic cells (mDCs), regulating the homing of immune cells to secondary lymphoid organs.^15^ CCL21 and CCL19, when present at elevated levels in the TME, can enhance antitumor immune responses and reverse immune evasion by tumor cells.^16^ In cancer vaccines, CCL21 has been used as an adjuvant to promote the recruitment and activation of dendritic cells (DCs) and T cells, thereby enhancing the efficacy of cancer vaccines in preclinical models and being evaluated in clinical trials for various cancers.^17^ So, investigating the mechanisms by which chemokines influence targeted therapy and immunotherapy in HCC is of great significance. In recent years, accumulating evidence has elucidated its different roles in various malignant tumors. High expression of CCR7 in tumor cells was proved to promote tumor metastasis such as esophageal squamous cell carcinoma, gastric cancer and colorectal cancer, the process could be achieved via epithelial-mesenchymal transition (EMT).^18^ Nevertheless, our previous studies also found CCL21/CCR7 axis could be harnessed in anti-tumor immunobiological therapy by its function of attracting CD8^+^T cells,^19,20^ and the combination of CCL21 and CD25 monoclonal antibody significantly inhibited the growth of HCC.^21^ A recent study in our institute has shown that the CCL21 determines immunotherapy response in HCC by affecting neutrophil polarization,^22^ which is consistent with a previous study that activating the CCL21/CCR7 axis by using CCL21 enhanced the antitumor responses.^23^ These findings indicate that the TME is complicated and the effects of the CCL21/CCR7 axis are related to the different responses to the drug treatments of HCC.

In this study, in order to explore the detailed relationships and related mechanisms, we used tissue microarray (TMA), spatial transcriptomic sequencing and single-cell sequencing to assess the complex TME mediated by CCL21/CCR7 axis with 2 cohorts (n1 = 240, n2 = 382, Zhongshan Hospital), 6 tumors and paired adjacent liver tissues from HCC patients, as well as pre-clinical experiments in vivo and in vitro, using CRISPR-cas9 system to establish CCR7 knock-out (KO) HCCLM3 cell line. The data showed high expression of CCR7 in tumor cells predicted lower Overall Survival (OS) of patients and knocking out CCR7 enhanced the sensitivity of HCC to sorafenib as a result of inhibiting EMT through the AKT and ERK signaling pathways. Unexpectedly, it was further revealed that VEGF-C promoted the formation of TLSs by activating lymphatic angiogenesis and the CCL21/CCR7 axis. As a result, it was finally demonstrated by pre-clinical experiments that VEGF-C increased the efficacy of anti-PD-1 immunotherapy.

This study highlights the importance of CCL21/CCR7 axis combined with VEGF-C and guides precision clinical treatments for HCC patients. Besides HCC, we have also investigated the role of CCR7 signal in other tumors derived from the endodermal epithelium and have sought similarities among tumors from the same embryonic origin. Interestingly, the data revealed a significant association between CCR7 expression and improved OS in patients with gastric cancer, colon-rectal cancer as well as lung adenocarcinoma and pancreatic cancer (data not shown in this study). CCR7 axis may serve as a common therapeutic target of pan-cancer derived from the endodermal epithelium, including HCC.

## Results

### CCR7 is highly expressed in tumor cells and predicts poor prognosis of HCC patients

**Fig. 1:**
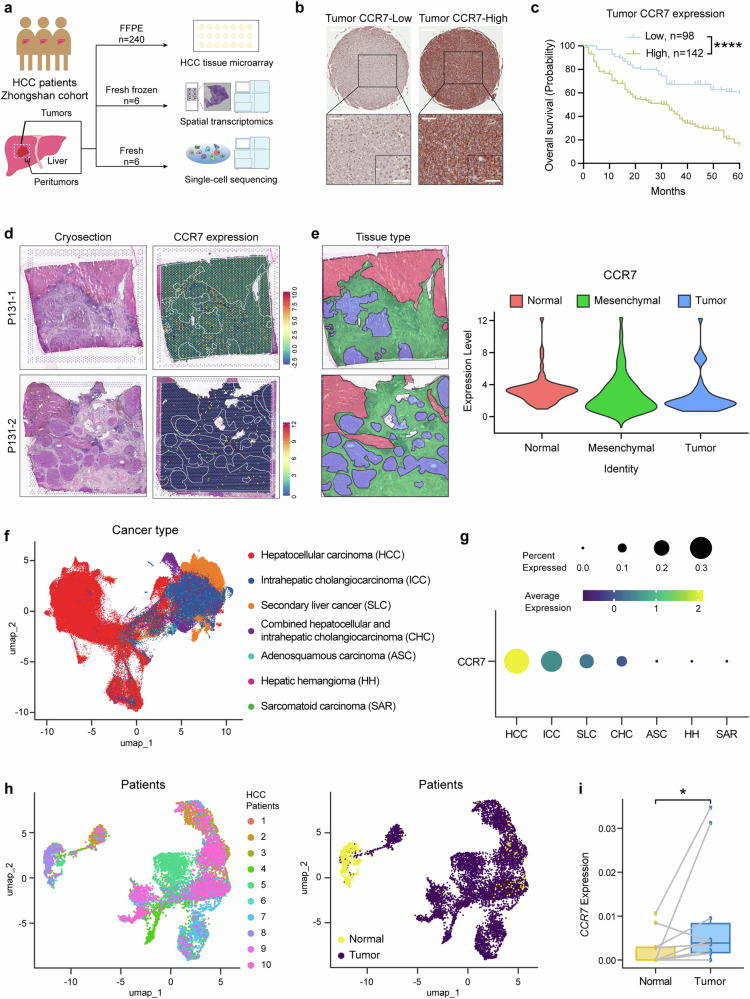
CCR7 is highly expressed in the tumor site. **a** Schematic overview of experiments in this study. **b** The representative image of CCR7-Low or CCR7-High expression in HCC TMA by IHC staining. Scale bar (top): 500 μm. Scale bar (bottom-left): 100 μm. Scale bar (bottom-right): 50 μm. **c** The survival curves of HCC patients with low or high tumor CCR7 expression identified by TMA, *n* = 240. **d** Two cryosections were applied for spatial transcriptomics (left). The expression of CCR7 in HCC tissues (right). **e** The sections were divided into different areas according to the results of dimensionality reduction clustering and pathological diagnosis (left). The distribution of CCR7 in HCC tissues (right). **f** The uniform manifold approximation and projection (UMAP) plot displaying the distribution of 192,670 epithelial cells across different liver neoplastic diseases. Cells were colored by disease categories. **g** The mean level of CCR7 gene expression and the percentage of CCR7^+^ cells determined by scRNA-seq across different liver neoplastic diseases contexts. **h** UMAP plots showing the identities of 9,315 epithelial cells derived from HCC patients with paired normal and tumor samples. Cells were colored by patient (left) and by tissue type (right). **i** The average single-cell expression of CCR7 in epithelial cells from paired normal and HCC tumor samples. *p* value by two-sided log-rank test. *p < 0.05, **p < 0.01, ***p < 0.001, ****p < 0.0001 is considered statistically significant. Data are represented as mean ± SD

Our previous study has proved that CC-chemokine receptor like 1 (CCRL1) impairs chemotactic events associated with CCR7 in the progression and metastasis of HCC.^24^ In order to explore the function of another CC-chemokine CCL21/CCR7 axis in HCC, we investigated the complex microenvironment of HCC by TMA, spatial transcriptomic sequencing and single-cell sequencing (Fig. 1a).

Firstly, the CCR7 expression by TMA of HCC patients (*n* = 240, one of our previous Zhongshan Hospital Cohort^13^) was identified by IHC. Positive staining of CCR7 protein was primarily observed on the membrane and in the cytoplasm of tumor cells, and based on the staining score of tumor tissue, HCC patients were divided into CCR7-high expression group and CCR7-low expression group (Fig. 1b). The OS of patients revealed a significant difference between the two groups analyzed by the Kaplan–Meier method and the CCR7-high group presented a significantly lower OS compared with the CCR7-low group (Fig. 1c, *p* < 0.0001). A positive correlation was also found between CCR7 expression and tumor size (*p* < 0.001), TNM stage (*p* < 0.001) as well as Age (*p* = 0.002) (Supplementary Fig. 1a and Supplementary Table 1). Furthermore, it was identified that CCR7 expression served as an independent predictor for OS of HCC patients based on multivariate and univariate analysis (Supplementary Fig. 1b and Supplementary Table 2). To detect CCR7 expression in mesenchymal sites, we observed the samples under different magnifications and were able to clearly identify the mesenchymal sites of the tumor tissues (outlined with dashed lines and separated from the tumor sites, Supplementary Fig. 2a). Representative images showing low (top) and high (bottom) CCR7 expression in mesenchymal sites were presented (Supplementary Fig. 2a). We further analyzed the correlation between the OS of patients and the CCR7 expression in mesenchymal sites of tumor tissues. The results showed that CCR7 expression in mesenchymal sites was not associated with OS of patients (Supplementary Fig. 2b).

Followingly, for further analysis of TME of HCC in detail, 6 tumors and paired adjacent liver tissues from HCC patients (Zhongshan Hospital) were collected for spatial transcriptomic sequencing. CCR7 and CCR7 closely related signals, such as CCL21, CCL19, Pecam1(CD31) and Lyve1, et al., were detected. The representative images of H&E staining (Left) and CCR7 expression of the spatial cluster distribution (Right) from one patient's tumor tissue were presented (Fig. 1d). Based on pathological diagnosis, the tissues were categorized into 3 regions: normal, mesenchymal and tumor (Fig. 1e, Left). It was shown that CCR7 expression was up-regulated both in the tumor sites and mesenchymal sites (Fig. 1e, Right).

To support these findings, we analyzed a published single-cell RNA-seq dataset from Chinese patients with primary liver cancer.^25^ The data shown that CCR7 was more widely and highly expressed in HCC epithelial cells, compared with other liver neoplastic diseases, for examples, intrahepatic cholangiocarcinoma (ICC), secondary liver cancer (SLC), combined hepatocellular and intrahepatic cholangiocarcinoma (CHC), adenosquamous carcinoma (ASC), hepatic hemangioma (HH) and sarcomatoid carcinoma (SAR) (Fig. 1f, g). Moreover, CCR7 was significantly higher expressed in epithelial cells of HCC tumor tissues, compared with paired adjacent normal liver tissues of HCC (Fig. 1h, i, *p* < 0.05, *n* = 10 patients).

### Knockout CCR7 on HCC tumor cells enhances sorafenib sensitivity by inhibiting EMT

It has recently been demonstrated that EMT is one of the chemotherapy resistance mechanisms in urothelial carcinoma (UC), additionally, UC with EMT features was associated with remodeled TME,^26^ which is consistent with one of our previous studies on cetuximab resistance in colorectal cancer.^27^ So, we explored whether EMT also participated in the mechanisms of sorafenib resistance in HCC.

**Fig. 2:**
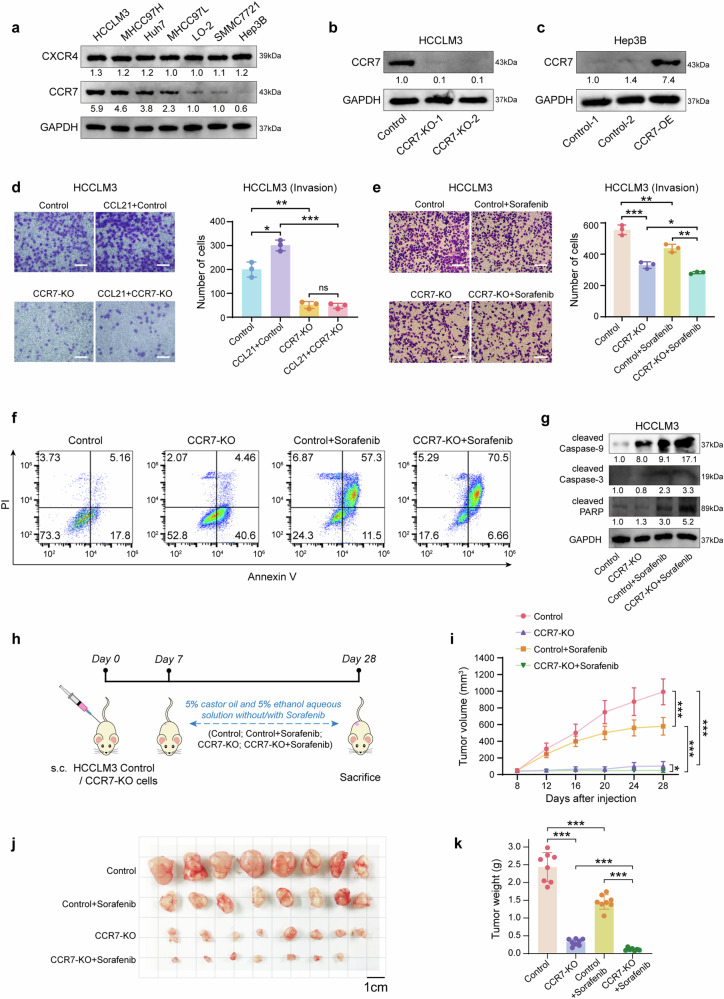
Blocking CCR7 signal enhances the sensibility of sorafenib. **a** The expression of CCR7 in human HCC cell lines was detected by Western Blot. **b**, **c** CCR7 expression after gene knockout (KO) on HCCLM3 (**b**) and overexpression (OE) on Hep3B (**c**). **d** The invasion ability of HCCLM3 was detected by trans-well assay after CCL21 stimulation (left). Quantitative analysis by using Image J (right). **e** The invasion ability of HCCLM3 was detected after the treatment of sorafenib (left). Quantitative analysis by using Image J (right). **f** The cell apoptosis was detected by flow cytometry. **g** The apoptosis-related proteins (cleaved Caspase-9, cleaved Caspase-3 and cleaved PARP) were detected by Western Blot. **h** The schedule of treatment in mice. **i** The tumor volume was monitored every four days in all groups. **j** The image of subcutaneous tumors on HCC mouse models was shown. **k** The tumor weight was presented. Data in (**h**–**k**) were conducted in the same batch, *n* = 8. *ns* > 0.05, *p < 0.05, **p < 0.01, ***p < 0.001 by unpaired two-tailed Student’s *t* test. Data are represented as mean ± SD

Firstly, we investigated the expression of different chemokine receptors in 6 human HCC tumor cell lines (HCCLM3, MHCC97H, Huh7, MHCC97L, SMMC7721, Hep3B) and 1 normal human hepatocyte line (LO2). Interestingly, it was shown that CCR7 expression was obviously different in different cell lines and displayed various levels, compared with CXC-chemokine receptor CXCR4 (Fig. 2a). With CRISPR-cas9 system (Supplementary Fig. 3), HCCLM3 was chosen to establish CCR7 knockout (KO) cell line due to its higher CCR7 expression, whereas Hep3B was chosen to establish CCR7 overexpression (OE) cell line in validation experiments due to its lower CCR7 expression (Fig. 2b, c). It was found that stimulating with CCL21, the invasion of HCCLM3-CCR7-KO decreased, compared with HCCLM3-Control (Fig. 2d, *p* < 0.001). CCK-8 analysis revealed that the proliferative ability of HCCLM3-CCR7-KO was significantly reduced, compared to HCCLM3-Control (Supplementary Fig. 4, *p* < 0.01). In addition, sorafenib treatment markedly inhibited the cell growth in the HCCLM3-Control+Sorafenib group, compared to the HCCLM3-Control group (Supplementary Fig. 4, *p* < 0.001). More importantly, HCCLM3-CCR7-KO+Sorafenib exhibited more sensitivity to sorafenib treatment with the lowest proliferative ability (Supplementary Fig. 4, *p* < 0.001). With the sorafenib treatment, HCCLM3-CCR7-KO exhibited lower invasion ability compared with HCCLM3-Control (Fig. 2e, *p* < 0.01). Flow cytometry analysis revealed a higher percentage of apoptotic cells in HCCLM3-CCR7-KO with the sorafenib treatment, compared to HCCLM3-Control (Fig. 2f). Additionally, apoptotic-related markers (cleaved Caspase-9, cleaved Caspase-3 and cleaved PARP) were up-regulated in the HCCLM3-CCR7-KO group treated with sorafenib (Fig. 2g).

Followingly, the subcutaneous tumor mouse models were established (Fig. 2h). It was found that tumors treated with sorafenib in the HCCLM3-CCR7-KO group grew more slowly than the HCCLM3-Control group (Fig. 2i, *p* < 0.001). Both tumor size and weight of the HCCLM3-CCR7-KO group were decreased compared with the HCCLM3-Control group (Fig. 2j, k, *p* < 0.001). It is suggested that blocking the CCR7 signal on HCC tumor cells enhances their sensitivity to sorafenib.

The migration ability of HCCLM3 was obviously enhanced with the stimulation of CCL21 for 48 h, whereas there was no significant stimulation effect on the migration ability of Hep3B (Supplementary Fig. 5a), while binding CCR7 with CCR7-specific neutralizing antibody (neu-CCR7) could block the migration and invasion of HCCLM3, respectively (Supplementary Fig. 5b, c). Furthermore, knocking out CCR7 in HCCLM3 by the CRISPR-cas9 system decreased the ability of migration in HCCLM3-CCR7-KO cells (Supplementary Fig. 5d, *p* < 0.01). At the same time, it was identified that both the invasion and migration abilities of the Hep3B-CCR7-OE group were increased, respectively, compared with Hep3B-CCR7-Control (Supplementary Fig. 5e, f, *p* < 0.05).

At the same time, we investigated the expression of different EMT-related markers in the seven different cell lines previously mentioned. It was identified that EMT-related markers, such as Vimentin and Twist, were obviously higher expressed whereas E-cadherin were obviously lower expressed in HCCLM3 cells, compared with Hep3B cells (Supplementary Fig. 6). With the CCL21 stimulation, the EMT markers were followingly analyzed in HCCLM3-neu-CCR7 group and HCCLM3-CCR7-KO group, it was identified that EMT-related markers were reversed in both groups, compared with HCCLM3-Control (Supplementary Fig. 7a, c), whereas there was no obvious change in Hep3B groups (Supplementary Fig. 7b). As expected, Overexpression CCR7 of Hep3B cells increased EMT-related markers, and it was identified that N-cadherin, β-catenin, Twist and Snail were up-regulated in the group of CCL21-CCR7-OE (Supplementary Fig. 7d). Meanwhile, comparing to HCCLM3-Control+Sorafenib, HCCLM3-CCR7-KO+Sorafenib down-regulated the expression of N-cadherin, Twist and Snail, whereas up-regulated the expression of E-cadherin, resulting in the lower EMT ability. (Supplementary Fig. 7e).

Finally, the downstream pathways of CCR7-mediated EMT were subsequently analyzed and determined that both p-AKT and p-ERK were significantly increased by CCL21 stimulation in HCCLM3 cells, which was reversed by neu-CCR7 and CCR7-Knockout, as shown in CCL21-neu-CCR7 group and CCL21-CCR7-KO group (Supplementary Fig. 8a, c). Meanwhile, there was no obvious change in Hep3B cells treated with CCL21 and neu-CCR7 (Supplementary Fig. 8b), whereas over expression of CCR7 in Hep3B enhanced the p-AKT and p-ERK in the CCL21-CCR7-OE group (Supplementary Fig. 8d). Meanwhile, HCCLM3-CCR7-KO+Sorafenib exhibited lower p-AKT expression than HCCLM3-Control+Sorafenib. (Supplementary Fig. 8e).

### High infiltration of CCR7^+^ immune cells in HCC tumor mesenchyme with higher expression of CCL21, CCL19 and Pecam1(CD31)

**Fig. 3:**
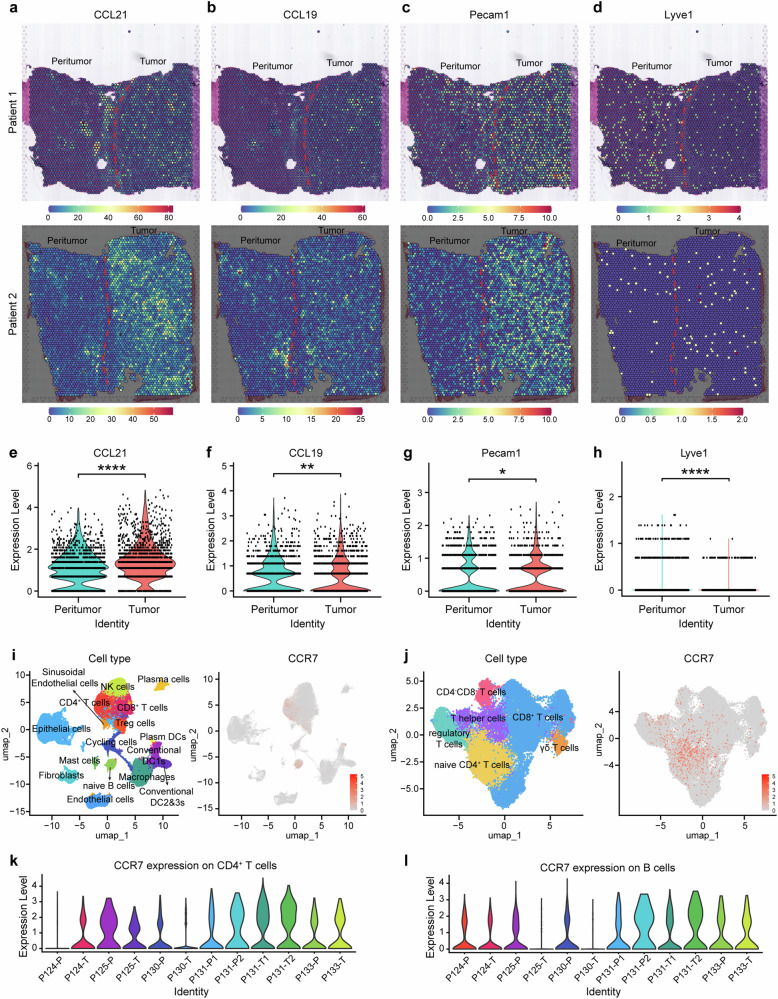
Spatial transcriptomics and single-cell sequencing reveal infiltration of CCR7^+^ immune cells in tumor. **a**–**h** The distribution of CCL21 (**a**), CCL19 (**b**), Pecam1 (**c**) and Lyve1 (**d**) in HCC tissues by spatial sequencing. Quantitative analysis of CCL21 (**e**), CCL19 (**f**), Pecam1 (**g**) and Lyve1 (**h**). **i** Single-cell sequencing was sorted from 6 tumors of HCC patients. UMAP visualization of single-cell sequencing data of non-tumor cells (left). CCR7 expression within non-tumor cells (right). **j** UMAP visualization of single-cell sequencing data of T cells (left). CCR7 expression within T cells (right). **k**, **l** CCR7 expression in CD4^+^ T cells (**k**) and B cells (**l**) between the tumor (-T) and corresponding peritumor tissues (-P). *p < 0.05, **p < 0.01, ***p < 0.001, ****p < 0.0001 by unpaired two-tailed Student’s *t* test. Data are represented as mean ± SD

Although high expression of CCR7 in tumor cells is related to EMT and sorafenib resistance, however, a higher CCR7 expression was also found in mesenchymal sites in HCC (Fig. 1e), which was often involved in the infiltration of CCR7^+^ immune cells. Therefore, in order to identify the complicated TME of HCC, we analyzed the expression of CCR7-related ligands based on the previous spatial transcriptomics analysis on the HCC patient dataset.^28^ The representative images of CCL21, CCL19 and Pecam1(CD31) as well as Lyve1 from Patient 1 and Patient 2 were presented (Fig. 3a–d). Compared with the peritumor area, CCL21 (Fig. 3e), CCL19 (Fig. 3f) and Pecam1(Fig. 3g) were more highly expressed in the tumor area, whereas Lyve1 had lower expression (Fig. 3h). To explore the complicated TME of HCC in detail, we collected fresh tumor tissues from Zhongshan Hospital and used single-cell sequencing to identify endothelial cells, epithelial cells, fibroblasts, cycling cells and immune cells. It was confirmed that endothelial cells, epithelial cells and fibroblasts showed lesser expression of CCR7, whereas CCR7 was predominantly expressed in immune cells, such as CD4^+^ T cells, naïve B cells, DCs and macrophages (MΦs) (Fig. 3i), especially naïve CD4^+^ T cells were the predominant population (Fig. 3j). Notably, CCR7 expression in CD4^+^ T cells and B cells was varied individually in the peritumor and tumor tissues (Fig. 3k, l).

### VEGF-C is positively related to CCR7 expression in mesenchymal sites and identified as an independent predictor for OS of HCC patients

As lower expression of Lyve1 in tumor sites was identified by spatial transcriptomics analysis in this study (Fig. 3h) and lymphatic angiogenesis was often driven by VEGF-families, we subsequently explored the correlation of VEGF-families with CCL21, CCL19, Pecam1 (CD31) and Lyve1 by the TCGA database of HCC. The data revealed that VEGF-C was significantly positively correlated with CCL21, CCL19 and Lyve1 (Supplementary Fig. 9a–c), whereas VEGF-D (Supplementary Fig. 9d-f) and VEGF-A (Supplementary Fig. 9g–i) had no obvious positive correlations with the CCR7 related ligands, suggesting the close correlation between CCR7^+^ infiltrated cells and VEGF-C.

**Fig. 4:**
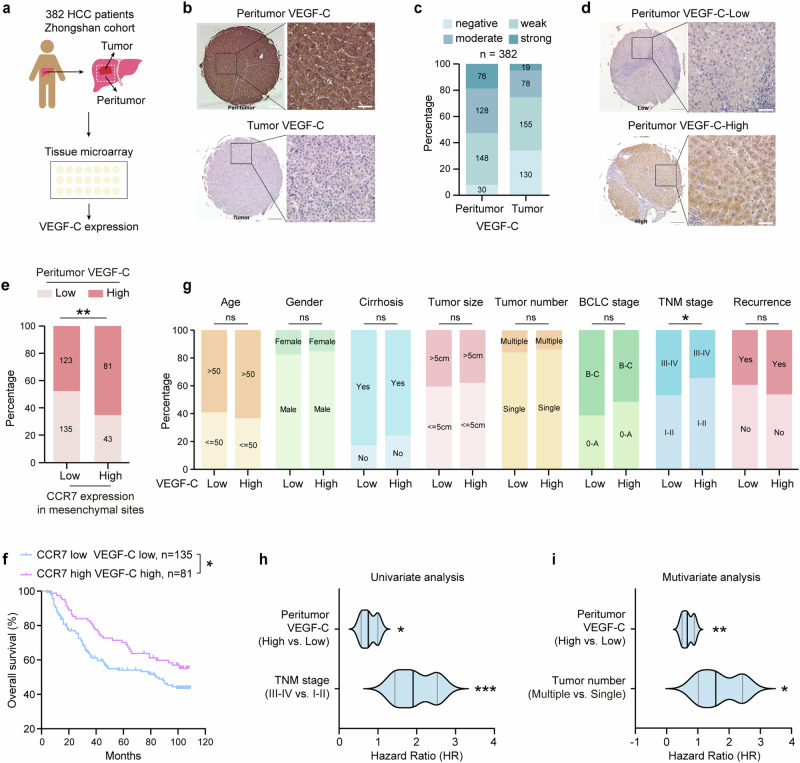
VEGF-C is positively related with CCR7 expression in mesenchymal sites and identified as an independent predictor for OS of HCC patients. **a** Schematic overview of HCC TMA collection and analysis (*n* = 382). **b** The representative images of VEGF-C expression in peritumor and tumor sites in TMA. Scale bar (left):500μm. Scale bar (right):50μm. **c** The intensity distribution of VEGF-C expression in peritumor and tumor sites from HCC TMA. **d** The representative image of VEGF-C-Low or VEGF-C-High expression in peritumor sites from HCC TMA (*n* = 382). Scale bar (left):500μm. Scale bar (right):50μm. **e** The correlation between VEGF-C and CCR7 expression in the mesenchymal sites in HCC TMA. **f** Kaplan–Meier analyses of OS based on CCR7 in mesenchymal sites and VEGF-C in peritumor sites from HCC TMA. **g** Correlations between VEGF-C expression in peritumor sites and clinical characteristics of patients with HCC from TMA. **h** Univariate analysis of factors associated with OS of HCC patients from TMA. **i** Multivariate analysis of factors associated with OS of HCC patients from TMA. Data in (**a**–**i**) were conducted in the same batch, *n* = 382. *p* value by two-sided log-rank test. *ns* > 0.05, *p < 0.05, **p < 0.01, ***p < 0.001 were considered statistically significant

Therefore, VEGF-C expression was further identified by TMA of HCC patients (*n* = 382, Zhongshan Hospital, Fig. 4a). The representative images of VEGF-C expression in tumor and peritumor were shown in Fig. 4b. The quantitative analysis data from tissue sections of 382 patients revealed that the intensity of VEGF-C expression in peritumor sites was higher than in tumor sites (Fig. 4c). Based on the different levels of VEGF-C expression in peritumor sites, all of 382 patients were divided into VEGF-C-High and VEGF-C-Low group. The representative images were shown in Fig. 4d. It was also found that the VEGF-C-High group was significantly correlated with high expression of CCR7 in mesenchymal sites, which was involved in the infiltration of CCR7^+^ immune cells (Fig. 4e, *n* = 382, *p* = 0.001). As a result, it was revealed that the OS of the VEGF-C-High group with high expression of CCR7 was significantly higher than the VEGF-C-Low group with low CCR7 expression (Fig. 4f, *p* = 0.0413), whereas there was no significant difference between other groups (data not shown). Meanwhile, VEGF-C expression in the peritumor was also associated with tumor TNM stage (Fig. 4g and Supplementary Table 3, *p* = 0.016). Univariate and multivariate analysis showed that VEGF-C was an independent predictor for the OS of patients with HCC (Fig. 4h, i and Supplementary Tables 4, 5).

### VEGF-C promotes the TLSs formation by lymphatic angiogenesis and activating CCL21/CCR7 axis

**Fig. 5:**
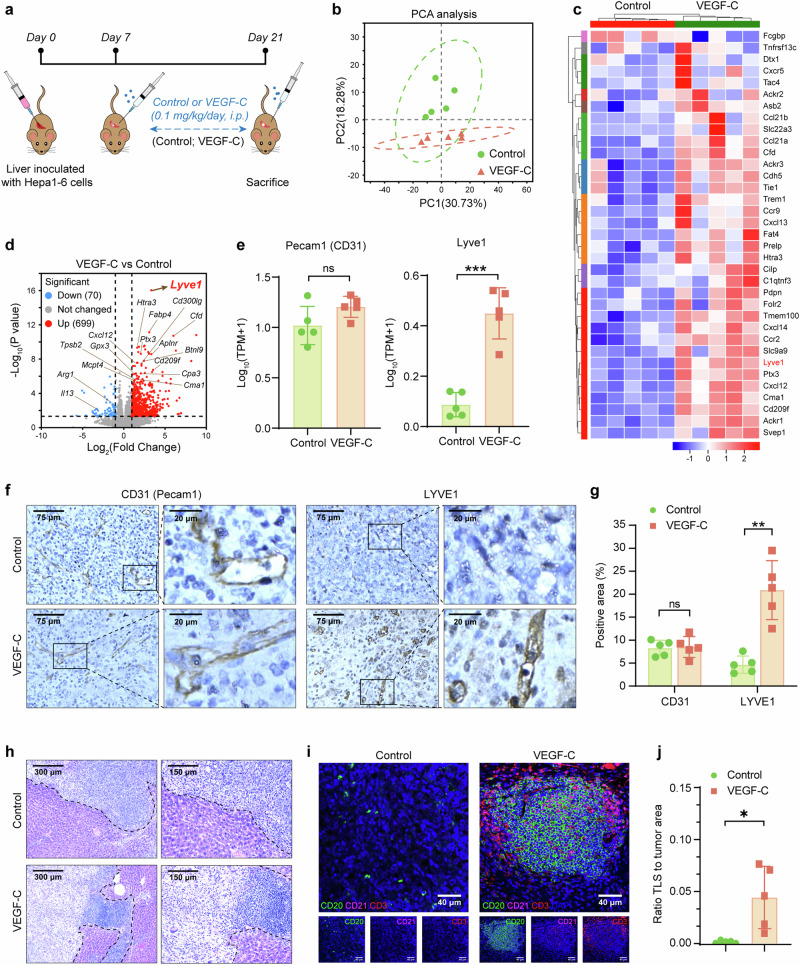
CCL21/CCR7 signal is activated by VEGF-C administration, which promotes the TLSs formation by lymphatic angiogenesis. **a** The schedule of treatments in the orthotopic HCC mouse model. **b** The PCA analysis of transcriptome sequencing of tumor tissues from the orthotopic HCC mouse model. **c**, **d** The heatmap (**c**) and volcano plots (**d**) of differential expression genes from transcriptome sequencing. **e** The expression of Pecam1 (left) and Lyve1 (right) genes in tumor from transcriptome sequencing. **f** The representative images of CD31 and LYVE1 in tumor by IHC staining. **g** Quantitative analysis of (**f**) by using Image J. **h** The tumor and peritumor samples from orthotopic HCC mouse tumors were observed by H&E staining. **i** Multiplex immunofluorescence assay of TLSs was performed in the orthotopic HCC mouse model. **j** The ratio of tumor area occupied by TLSs was quantitatively analyzed using Image J. Data in (**a**–**j**) were conducted in the same batch, *n* = 5. *ns* > 0.05, *p < 0.05, **p < 0.01, ***p < 0.001 by unpaired two-tailed Student’s *t* test. Data are represented as mean ± SD

To explore the detailed role of VEGF-C on HCC in vivo, the orthotopic HCC mouse models were established with or without VEGF-C treatment (Fig. 5a). In the VEGF-C group, the mice were treated with systemic VEGF-C administration, and the concentration of VEGF-C in the serum significantly increased after administration (Supplementary Fig. 10a). In order to explore the effect of VEGF-C on tumor growth in the orthotopic HCC mouse model, the ratio of the tumor-bearing liver weight to body weight in mice was analyzed. It showed no significant difference between the Control group and the VEGF-C group, indicating that VEGF-C treatment had no effect on tumor growth in these models (Supplementary Fig. 10b, c). The tumor tissues were collected to perform transcriptome sequencing. With the help of Principal Component Analysis (PCA) and heatmap statistical visualization, it was identified that VEGF-C treatment obviously changed the transcriptome sequencing of tumor tissues, compared with Control group (Fig. 5b, c). Among the differentially expressed genes between the two groups, it was found that the lymphatic endothelial marker *Lyve1* was significantly upregulated in the volcano plots of VEGF-C group vs Control group (Fig. 5d), while the vascular endothelial marker *Pecam1* showed no significant change between the two groups (Fig. 5e). CD31(*Pecam1*) and LYVE1(*Lyve1*) expression in tumor tissues of the orthotopic HCC mouse models were also identified by IHC analysis, which was shown by the representative images (Fig. 5f). It was confirmed by quantitative analysis with Image J that comparing with control group, the exogenous VEGF-C treatment significantly promoted the lymphatic angiogenesis within the HCC tumor sites (Fig. 5g, *p* < 0.01). Unexpectedly, TLSs were found in the tumor sites of the VEGF-C treatment group (Fig. 5h), which were further confirmed by multiplex immunofluorescence assay, indicating the presence of CD20^+^ B cells, CD3^+^ T cells and CD21^+^ follicular DCs, which were organized into lymphoid structures (Fig. 5i). VEGF-C treatment significantly increased the TLS ratio to tumor area by quantitative analysis (Fig. 5j).

Followingly, the differentially expressed genes by transcriptome sequencing were analyzed with GO enrichment. It was found that there was a significant enrichment in the regulation of chemotaxis pathway in the VEGF-C treatment group (Supplementary Fig. 11a). CCL21 (*Ccl21a* and *Ccl21b*), the ligand of CCR7, rather than CCL19, was significantly upregulated in the VEGF-C treatment group (Supplementary Fig. 11b, c). Although there was no obvious change of CCR7 expression in the VEGF-C treatment group (Supplementary Fig. 11d), it was identified by IHC that not only CCL21 expression, but also the infiltration of CCR7^+^ cells into mesenchymal sites was significantly increased in the VEGF-C treatment group (Supplementary Fig. 11e–g).

To explore the related biological mechanisms, the effect of VEGF-C on mouse lymphatic endothelial cells (mLECs) was tested in vitro. It was identified that VEGF-C improved the tube formation of mLECs (Supplementary Fig. 12a, b). In addition, it was also identified by ELISA the CCL21 concentration in the supernatant of mLECs culture was significantly increased with VEGF-C treatment (Supplementary Fig. 12c, 56.08 ± 5.32 pg/ml *vs* 51.98 ± 5.47 pg/ml, *p* < 0.05). As a result, the improved migration ability of CCR7^+^macrophage (RAW264.7) was identified by co-culture with the supernatant of mLECs treated with VEGF-C (Supplementary Fig. 12d, e).

To investigate the relationships among VEGF-C, CCL21, LYVE1, and CCR7 in clinical HCC patients, we analyzed the spatial distribution of them based on the previous spatial transcriptomics dataset from HCC patients.^28^ The Pearson analysis was used to assess the correlation among VEGF-C, CCL21, LYVE1, and CCR7. The analysis revealed co-localization and strong positive correlations between the expressions of VEGF-C and CCL21 (Supplementary Fig. 13a, b), VEGF-C and LYVE1 (Supplementary Fig. 13c, d), VEGF-C and CCR7 (Supplementary Fig. 13e, f), as well as CCL21 and CCR7 (Supplementary Fig. 13g, h). These findings suggested that VEGF-C induced lymphatic angiogenesis in HCC tumors (VEGF-C vs. LYVE1), then CCR7^+^ cells were introduced into the tumor through lymphatic vessels under the chemotactic influence of CCL21 (VEGF-C vs. CCL21, VEGF-C vs. CCR7, and CCL21 vs. CCR7). Furthermore, we performed double immunostaining on the tumor tissues from orthotopic HCC mouse models. The experimental results revealed the presence of CCR7^+^ cells within lymphatic vessels induced by VEGF-C, which indicated that CCR7^+^ cells migrated into the tumor through the lymphatic vessels (Supplementary Fig. 14).

Additionally, based on the previous dataset of transcriptome sequencing on the mice tumor tissues in this study, it was also revealed that a group of genes associated with tumor immunology, such as Cd209 families (CD209a, CD209d, CD209f), CD36, CD300lg, et al. were upregulated in the VEGF-C treatment group (Supplementary Fig. 15a). Especially, with GSEA (Gene Set Enrichment Analysis) sorting, it was revealed that VEGF-C upregulated several signal pathways, such as the SIGNALING BY THE B_CELL_ RECEPTOR_BCR (Supplementary Fig. 15b) and INITIAL_TRIGGERING_OF_COMPLEMENT (Supplementary Fig. 15c), et al. The increased infiltration of immune cells (CD11b^+^, CD68^+^, MHC II^+^, CD3^+^) into the tumor sites in the VEGF-C treatment group was demonstrated by IHC staining (Supplementary Fig. 15d, e).

### VEGF-C increases the efficacy of anti-PD-1 immunotherapy for HCC

**Fig. 6:**
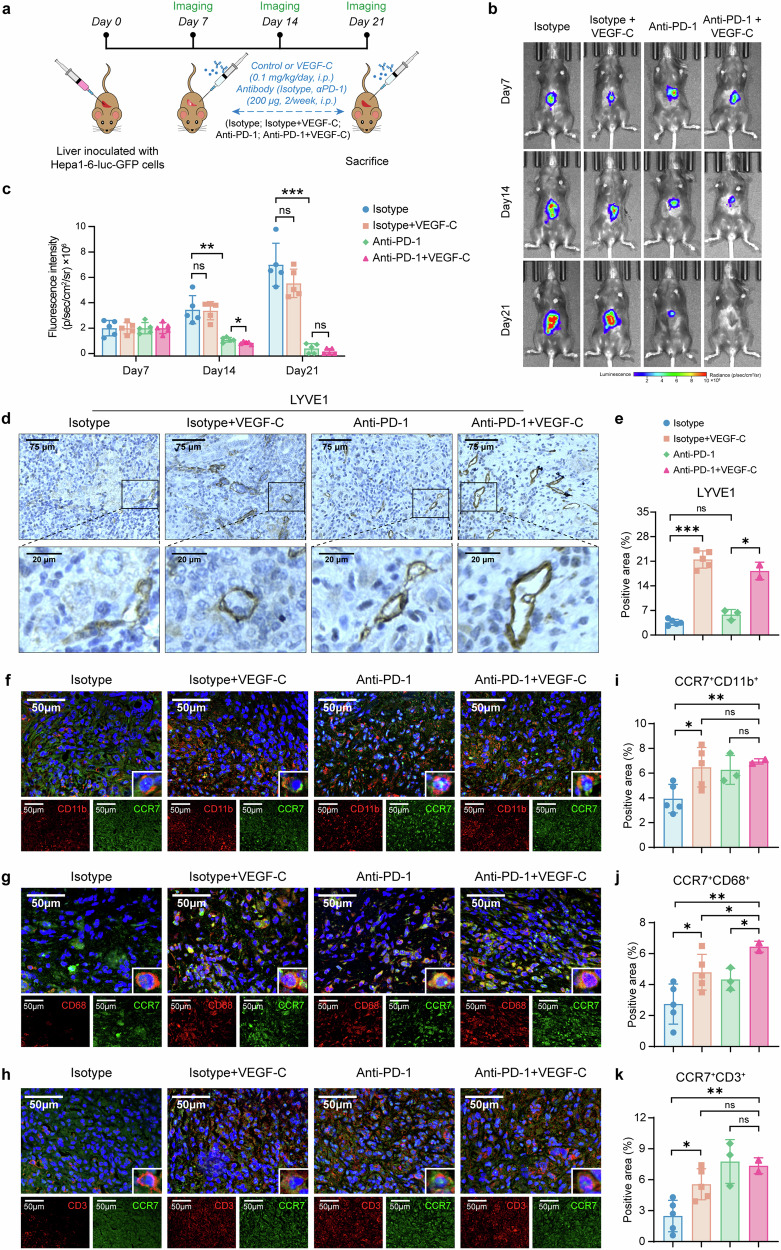
VEGF-C increases the efficacy of Anti-PD-1 immunotherapy for HCC. **a** The schedule of imaging and treatments. **b** The representative images of luciferase signal in vivo. **c** The luciferase signal was monitored by in vivo imaging system once a week. *n* = 5. **d** The representative images of LYVE1 in tumor by immunohistochemical staining. **e** Quantitative analysis of (**d**) by Image J. **f**–**k** The representative images of CCR7^+^CD11b^+^ cells (**f**), CCR7^+^CD68^+^ cells (**g**), CCR7^+^CD3^+^ cells (**h**) in tumor by immunofluorescence staining. Quantitative analysis of CCR7^+^CD11b^+^ cells (**i**), CCR7^+^CD68^+^ cells (**j**), CCR7^+^CD3^+^ cells (**k**) by Image J. Data in (**a**–**k**) were conducted in the same batch, *n* = 5. *ns* > 0.05, *p < 0.05, ***p < 0.001 by unpaired two-tailed Student’s *t* test. Data are represented as mean ± SD

Immunotherapy, particularly anti-PD-1, has been widely used in the clinical treatment of HCC, but the response rate was limited.^3^ Whereas TLSs in tumor tissues have been recently proven to promote immunotherapy response in previous studies,^11,29^ therefore we proposed the hypothesis that VEGF-C might increase the response of anti-PD-1 immunotherapy. The orthotopic HCC mouse models were established with Hepa1-6 cells carrying the luciferase reporter gene (Hepa1-6-luc-GFP), and based on the treatments, the animals were divided into 4 groups: Isotype, Isotype + VEGF-C, Anti-PD-1, Anti-PD-1 + VEGF-C (Fig. 6a). The concentration of VEGF-C was significantly increased in the serum of mice after VEGF-C administration (in Isotype + VEGF-C group and Anti-PD-1 + VEGF-C group, as shown in Supplementary Fig. 16). The luciferase signals were reported with In Vivo Imaging System, which were presented by the representative images (Fig. 6b). Based on the quantitative analysis of luciferase signals, it was revealed that the fluorescence intensity of Anti-PD-1 + VEGF-C group was weaker on Day 14 than the Anti-PD-1 group, suggesting the combination of VEGF-C enhanced the efficacy of Anti-PD-1 immunotherapy (Fig. 6c). It was followingly identified by IHC analysis that VEGF-C treatment promoted the lymph angiogenesis in tumor (Fig. 6d, e), whereas there was no obvious effect on the angiogenesis (Supplementary Fig. 17).

Besides, the infiltration of CCR7^+^ immune cells into the tumor sites through lymphatic vessels were also identified by immunofluorescent staining, such as CCR7^+^CD11b^+^, CCR7^+^CD68^+^and CCR7^+^CD3^+^ cells (Fig. 6f-h). The quantitative analysis data showed that compared with Isotype, Isotype + VEGF-C could increase the infiltration of CCR7^+^CD11b^+^ cells, CCR7^+^CD68^+^ cells and CCR7^+^CD3^+^ cells (Fig. 6i–k, *p* < 0.05), while Anti-PD-1 + VEGF-C increased more infiltrated CCR7^+^ cells (Fig. 6i-k, *p* < 0.01). These findings indicated that VEGF-C treatment increased the infiltration of CCR7^+^ immune cells, which contributed to the enhanced efficacy of anti-PD-1 immunotherapy and resulted in the inhibition of tumor progression.

Additionally, we analyzed the relationships among TLSs, CCR7, VEGF-C in tumor tissues from clinical HCC patients treated with Anti-PD-1 immunotherapy based on publicly available datasets.^30,31^ These studies divided HCC patients into Non-responders and Responders to immunotherapy, and representative HE-stained images were shown in Supplementary Fig. 18a.^30^ Using spatial transcriptomics and bulk transcriptome sequencing, we examined the spatial distribution of TLSs and the related gene expressions marked by a published TLS-related gene signature,^32^ as well as the distribution and expressions of CCR7 and VEGF-C in tumor tissues (Supplementary Fig. 18b–d). The results revealed that TLSs were more frequently observed in tumors in Responders group, and the expressions of TLS-related genes were significantly higher compared to the Non-responders group (Supplementary Fig. 18e). Meanwhile, the expression level of CCR7 × VEGF-C (a combined measure of CCR7 and VEGF-C expressions) was higher in the tumor tissues from the Responders group (Supplementary Fig. 18f). Further analysis demonstrated a significant positive correlation between the expressions of TLS-related genes and CCR7 × VEGF-C (Supplementary Fig. 18g). These findings indicated the closely related relationships among TLSs, CCR7, VEGF-C in HCC tumor tissues. Moreover, the presence of TLSs and the elevated expressions of CCR7 and VEGF-C in tumor tissues can serve as indicators of a favorable response to anti-PD-1 immunotherapy in HCC patients.

## Discussion

**Fig. 7:**
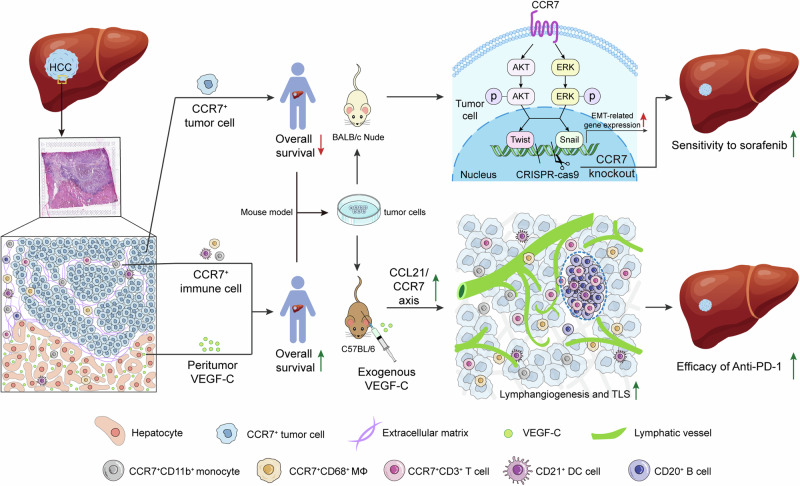
The graphical abstract of the study. CCR7 higher expression in tumor cells predicts lower Overall Survival (OS) of HCC patients, whereas knocking out CCR7 by CRISPR-cas9 enhances the sensitivity of tumor cells to sorafenib by inhibiting epithelial–mesenchymal transition (EMT) through the AKT and ERK signaling pathways. CCR7 is also expressed in stromal cells with the increased infiltration of CCR7^+^ immune cells into the tumor mesenchyme, which is enhanced by VEGF-C treatment by activating lymphatic angiogenesis and CCL21/CCR7 axis. High expression of VEGF-C in peritumor is identified as an independent predictor for higher OS of HCC patients, which is harnessed to increase the efficacy of anti-PD-1 immunotherapy by activating lymphatic angiogenesis and CCL21/CCR7 axis, and promoting the formation of tertiary lymphoid structures (TLSs). This figure was created using the online tools of SMART and Adobe Illustrator software

Recently, in clinical treatment strategies for HCC, sorafenib and Anti-PD-1 immunotherapy has benefited only a fraction of patients.^2,3,33^ In order to expand the scope of beneficiary patients, we conducted an in-depth analysis of the HCC microenvironment and explored the role of the CCL21/CCR7 axis in different components to accurately guide the precision treatment for HCC. In this study, we found that CCR7 was expressed in both tumor cells and stromal cells through the IHC analysis of TMA, spatial transcriptome and single-cell sequencing analysis of HCC tissues, but their effects were opposite. On the one hand, knocking out the expression of CCR7 in tumor cells through the CRISPR-cas9 system enhanced their sensitivity to sorafenib. On the other hand, the infiltration of CCR7^+^ immune cells in tumor mesenchyme was promoted by VEGF-C administration, which increased the efficacy of anti-PD-1 immunotherapy in HCC mouse models (Fig. 7).

In tumor cells, higher expression of CCR7 is related to lower OS of HCC patients, which makes CCR7 expression serve as an independent prognostic factor for HCC patients. Furthermore, it was found that knockout CCR7 on HCC tumor cells inhibited the occurrence of EMT and the development of sorafenib resistance. EMT is a fundamental process underlying the invasion and metastasis of various tumors.^34–36^ Increasing evidence suggests that EMT also plays a crucial role in the development of drug resistance.^37,38^ As the first-generation targeted drug in the past, sorafenib has demonstrated the benefits for advanced-stage HCC patients. Unfortunately, due to the emergence of resistance, an increasing number of patients fail to achieve long-term survival from sorafenib therapy.^5,39^ Prolonged exposure to sorafenib leads to the gradual transformation of HCC cells into a mesenchymal state and the acquisition of sorafenib resistance following EMT^40,41^ In vitro, we found that sorafenib significantly inhibited EMT in CCR7-KO cells, and the AKT and ERK signaling pathways were involved in this process. In addition, in pre-clinical mouse models, sorafenib significantly inhibited the growth of CCR7-KO tumors, providing the possibility for the resurgence of sorafenib.

In stromal cells, single cell sequencing analysis revealed that CCR7 was mainly expressed in immune cells, such as CD4^+^T cells, naïve B cells, DCs and MΦs. The role of CCR7 in immune cells is multifaceted. In T cells, CCR7 interacts with its ligand CCL21 and binds to TCR to jointly stimulate T cell activation.^42^ CCR7 also participated in the migration of neutrophils, DCs, and other immune cells to lymph nodes.^43,44^ The increased expression of CCR7 on DCs induced cell shape changes that facilitated their migration from peripheral tissues to lymph nodes, and reprogrammed their transcription to determine their immunoregulatory properties.^45^ Additionally, CCR7 serves as a marker for M1 macrophages, which exerts anti-tumor effects.^46^ CCL21/CCR7 axis not only enhances the directional movement of immune cells, but also participates in the antitumor response of immune cells. In this study, we systematically administered VEGF-C to orthotopic HCC mouse models, which promoted intratumoral lymphangiogenesis to provide pathways for CCR7^+^ monocytes, CCR7^+^ MΦs, and CCR7^+^ T cells to enter the tumor. Furthermore, in the context of immunotherapy, VEGF-C exerted anti-tumor effects through the infiltration of CCR7^+^ immune cells, enhancing the therapeutic efficacy of anti-PD-1 on HCC models. Similarly, in melanoma, tumors with elevated VEGF-C levels were associated with increased intratumoral lymphatic vessels, attracting a greater influx of CCR7^+^ immune cells.^47,48^ In brain tumors, VEGF-C promoted the formation of lymphatic vessels and enhanced the effectiveness of anti-PD-1/CTLA-4 combination immunotherapy through the CCL21/CCR7 axis.^49,50^ Our pre-clinical research findings were in accord with recent studies, indicating that CCR7 in stromal cells enhanced the efficacy of anti-PD-1 immunotherapy combined with VEGF-C, providing an improved therapeutic strategy for HCC.

In this research, one unanticipated finding was that only blocking the CCR7 signal on HCCLM3 already showed significant tumor inhibition in HCC subcutaneous mouse models without sorafenib treatment. Even though the combination with sorafenib treatment was more effective, we revealed the dramatic impact of intervention on the CCR7 signal in vivo experiments. This result provides strong evidence for the importance of the CCR7 axis in HCC TME. In addition, what is unexpected is that TLSs were found in tumor sites after VEGF-C administration. Further histochemical staining confirmed this result by the presence of key immune cells, including B cells, T cells and DC cells, and their organized spatial distribution inside. Previous studies have reported that B cells and T cells within TLSs interacted closely, which was associated with a “hot” TME.^51,52^ TLSs themselves have been proven to be associated with the response to immunotherapy.^53,54^ Our findings have extended the knowledge of VEGF-C function, which is not limited to induce lymphatic angiogenesis. Based on our experimental observations, VEGF-C showed multiple mechanisms to enhance anti-tumor immunity in vivo, such as providing an inflow channel for immune cells and constructing TLSs locally.

HCC is a highly vascularized tumor.^55^ Currently, the commonly used anti-angiogenic agent in the clinical treatment of HCC is Bevacizumab, which primarily binds to VEGF-A to exert its anti-angiogenic effects.^56–58^ Although VEGF-C is also a member of the VEGF family, its primary role is not to promote angiogenesis but to facilitate lymphangiogenesis.^48,59^ Lymphatic vessels play an active role in the tumor immune microenvironment, acting as a bridge for immune communication between the tumor and host, thereby promoting immune cell infiltration.^60^ Moreover, VEGF-C is a secreted protein, and the detection of VEGF-C levels in blood samples can provide a more comprehensive reflection of its biological functions and clinical significance in vivo. In melanoma, serum VEGF-C levels have been found to be significantly associated with the efficacy of immunotherapy.^47^ In the orthotopic HCC mouse models used in this study, the concentration of VEGF-C in the serum significantly increased after VEGF-C administration. Nevertheless, due to the homogenization and the limited number of the animal models, the predictive value of serum VEGF-C in HCC treatment could not be fully elucidated.

Although we demonstrated that blocking CCR7 signal on tumor cells enhanced the sorafenib sensitivity of HCC, which was mediated by inhibiting the EMT process and leaded to the tumor suppression, the mechanisms behind these changes remain unclear. When we focused on the high infiltration of CCR7^+^ immune cells in tumor mesenchyme, we should give enough thought to the complicated TME of HCC. No matter whether on HCC clinical specimens and in tumor-bearing mouse models, we still cannot rule out the influence of chemokine axes other than CCR7. The potential cross-talking of different chemokine axis was warranted further investigation because of their significant influence on TME.^61^ In our orthotopic HCC animal models, systemic administration of VEGF-C promoted the intratumoral lymphangiogenesis and enhanced the efficacy of anti-PD-1 immunotherapy. In addition, the roles and underlying mechanisms of CCR7 and VEGF-C in HCC should be further validated by using the KO mice of CCR7 and VEGF-C, including investigating the effects of CCR7 and VEGF-C on tumor growth, metastasis, and the response to sorafenib and anti-PD-1 immunotherapy.

In summary, our study reveals that the CCR7 signal may favor a pro- or anti-tumor function depending on where it is expressed in HCC. CCR7 expression can act as a biomarker for predicting response to targeted or immune checkpoint inhibitor therapies in HCC patients. The CCR7 signal needs to be measured categorically and treated individually in patients. The precision regulation of CCR7 signal, for example blocking in tumor cells and boosting in immune cells, is expected to a promising therapeutic strategy for HCC.

## Materials and Methods

### Ethics statements

All animals were purchased from the Shanghai Institute of Material Medicine of Chinese Academy of Science. All animal experimental procedures were carried out in strict compliance with the National Institutes of Health Guide for the Care and Use of Laboratory Animals, and were approved by the Animal Care and Use Committee of Basic Medical School, Fudan University (Approval No. 20140226-060, 20190221-106, 20230301-167).

All procedures in the study of HCC patient samples were approved by the Ethics Committee of Fudan University (Approval No. 2019-011) and the Ethics Committee of Zhongshan Hospital (Approval No. B2024-584). Informed consent was obtained from each enrolled patient. The study complies with all relevant ethical regulations.

### Preparation, sequencing and bioinformatics analyses of HCC patient samples

The samples of spatial transcriptomics were harvested from the junction of cancer tissue and adjacent-carcinoma tissue. Samples were treated with a frozen embedding agent (Invitrogen) and stored at −80 °C. The spatial transcriptomics was performed using the Visium Spatial Gene Expression platform (10X Genomics) by Shanghai Crystal Energy Biochip Co., Ltd. The Pmeabilisation step was about 15 min. The sample was quantified and the quality was tested using an Agilent 2100 Bioanalyzer.

The detailed progress of sample preparation of single-cell sequencing, the process of data quality and analysis, were same as our previous study.^62^ The spot-filtering parameters of spatial sequencing were 200 < feature of RNA < 4000. Data analysis was done using the R Software (version 4.2.1). Genes with *p* < 0.05, fold change > 2, and CT > 0.25 were considered relevant. Principal components analysis (PCA) was performed for dimension reduction, followed by clustering using the Louvain algorithm. The Harmony package was applied to remove batch effects across different samples. Clustering results were visualized using the UMAP algorithms. All codes of the bioinformatics analysis have been uploaded on figureshare (10.6084/m9.figshare.24486565).

### Human HCC tissue microarray

In our retrospective analyses, the study of tumor CCR7 expression was based on the HCC tissue microarray (TMA) with 240 patients. The detailed information about the specimens referred to the published paper from the partners of our research.^24,63^ The study of peritumor VEGF-C expression was based on another HCC TMA with 382 patients, as previously described.^24,63^ All specimens were pathologically reassessed independently by two gastroenterology pathologists blinded to the data.

The expression levels of CCR7 and VEGF-C of tumor and peritumor tissues were detected by immunohistochemical staining. The stained section was observed by the microscope (EVOS M7000 system), and the camera settings were the same when shooting each photo. The two pathologists independently read the film, and the two pathologists were unaware of the patient’s clinical outcome. The intensity scoring criteria of immunohistochemical staining was as follows: 0 = negative; 1 = weak; 2 = moderate; 3 = strong. 0, 1 were defined as low expression, 2, 3 were defined as high expression. If the opinions of two pathologists conflicted, the third one would conduct a re-evaluation. The majority opinion would serve as the final determination.

### Immunohistochemistry

According to our previous research,^64^ immunohistochemistry (IHC) was performed using a two-step protocol (Novolink Polymer Detection System, UK). Horseradish peroxidase (HRP)-conjugated secondary antibodies and 3, 3′-diaminobenzidine (DAB) solution (Sigma-Aldrich, USA) were used in DAB staining. Fluorescein isothiocyanate secondary antibodies and 4′,6-diamidino-2-phenylindole (DAPI, Sigma, USA) were used in immunofluorescence staining. Images were captured using the microscope (EVOS M7000 system) and quantitatively analyzed by using Image J.

The multiplex immunofluorescence assay (Zen-Bioscience, China) was performed according to the manufacturer’s instructions. The immunofluorescence staining was analyzed using a laser-scanning confocal microscope (magnification 400×, TCS-SP5, Leica Camera AG, Germany). Based on the H&E staining and immunofluorescence staining, the area occupied by the TLS and the total area of the tumor were quantitatively analyzed using Image J and obtained their ratio, as previously described.^11^

The information about antibodies were included in the Supplementary Table 6. Negative controls without primary antibodies were performed in all assays.

### Cell lines and treatment

Human and mouse HCC cell lines were obtained from ATCC or Cell Bank of Shanghai Institutes of Biological Sciences, Chinese Academy of Sciences (details in Supplementary Table 7). The stepwise metastatic human HCC cell lines MHCC97L and SMMC-7721 were established in Liver Cancer Institute, Fudan University. Cells were cultured in DMEM or MEM with high glucose (Gibco, USA) supplemented with 10% fetal bovine serum (FBS, Gibco, USA). All cells were incubated at 37˚C in a humidified atmosphere containing 5% CO_2_.

In the sorafenib (Bayer, Germany) treatment groups, HCCLM3 cells were incubated with 10 µM sorafenib for 48 h before proceeding with the subsequent experiments and analysis. In the CCL21 (PeproTech, USA) treatment groups, HCCLM3 and Hep3B cells were treated with 100 ng/mL CCL21 for 48 h before proceeding with the subsequent experiments and analysis.

### Western blotting assay

According to our previous research,^64^ cells were treated as described and harvested at the indicated time to prepare into protein samples. The samples were resolved by polyacrylamide gel electrophoresis and analyzed by immunoblotting with primary antibodies. GAPDH was used as a loading control. After incubation with HRP-conjugated secondary antibodies (KPL, USA), signals were visualized by ECL system (Thermo Fisher Scientific Inc., USA) and quantified by Gel-Pro analyzer (Media Cybernetics Inc., USA). Experiments were performed in triplicate. The information about antibodies were included in the Supplementary Table 6.

### Construction of CCR7 knockout HCC cells

The lentiviral vector for CCR7 gene knockout (KO), based on the CRISPR/Cas9 gene-editing system, was constructed by JiKai Gene Technology (Shanghai, China) and transfected into HCCLM3 cells with original higher CCR7 expression. The target sequence of sgRNAs after screening and the structure diagram of Cas9 plasmid were listed in Supplementary Table 8 and Supplementary Fig. 3. Single-clone sequencing confirmed the insertion of the target sgRNA after transfection on DH5α cells. The KO lentivirus was produced by co-transfection of sgRNA recombinant plasmids and packaging plasmids into HEK293T cells. The stable HCCLM3-CCR7-KO strain was successfully established after transfection. The KO efficiency was evaluated by Western blotting. HCCLM3-Control transfected with control vector, containing a random sequence that did not target any genes, was used as a negative control.

### Construction of CCR7 overexpression HCC cells

For CCR7 overexpression (OE), the lentiviral vector was constructed by JiKai Gene Technology and transfected into Hep3B cells with an initial lower CCR7 expression. Single-clone sequencing confirmed the donor plasmid containing the CCR7 gene. The sequence of full-length CCR7 was listed in Supplementary Table 9. The Hep3B-CCR7-OE stable strain was successfully established after transfection with the OE lentiviral vector. The OE efficiency was evaluated by Western blotting. Hep3B-Control, transfected with control vector, was used as control.

### Enzyme-linked immunosorbent assay

Cell suspension (2 ml volume) containing 1 × 10^6^ mLECs was added to each 3.5 cm culture dish. During the cell culture, mLECs were treated daily with or without 100 ng/mL of VEGF-C (Acro Biosystems, China). After 72 h of incubation, the cell culture medium was collected, centrifuged at 1000 g for 20 min, and the supernatant was used for subsequent detection.

Mouse CCL21 ELISA plates (catalog No.BE13009M1) were purchased from Bioagrio Biotechnology Co., Ltd. (Shanghai, China), and used according to the manufacturer’s instructions. The optical density values were measured at 450 nm.

### Cell migration and invasion trans-well assays

According to our previous research,^64^ cell migration assay was performed using 24-well trans-well with 8.0 μm pore polycarbonate membrane insert (Corning, USA). For HCC cells (HCCLM3 and Hep3B), 1×10^5^ cells were suspended in 100 μL high glucose DMEM (Gibco, USA) supplemented with 1% FBS (Gibco, USA), and added into the upper chamber. The lower chamber was filled with 600 μL high-glucose DMEM supplemented with 10% FBS. After incubation for 24 h, the cells on the upper surface of the membrane were removed. The migrated cells on the lower surface were fixed with 4% paraformaldehyde, stained with 0.1% crystal violet for 15 min at room temperature. Cell invasion assay was performed in a similar manner, except the cells were seeded onto a Matrigel (BD Biosciences, USA) coated filter. Images were captured using the microscope (EVOS M7000 system) and quantitatively analyzed by using Image J.

For RAW264.7 cells, 1 × 10^6^ cells were suspended in 200 μL of high-glucose DMEM (Gibco, USA), and added into the upper chamber. The lower chamber was filled with 300 μL high glucose DMEM supplemented with 10% FBS and 300 μL of supernatant from mLECs treated with VEGF-C. After incubation for 48 h, cells on the upper surface of the membrane were removed, fixed with paraformaldehyde, and stained with crystal violet. Images were captured using the microscope (EVOS M7000 system). Images were captured using the microscope (EVOS M7000 system) and quantitatively analyzed by using Image J.

### CCK-8 analysis

Cell proliferation was quantitatively evaluated using the CCK-8 kit (Dojindo, Japan). The experimental operation was carried out in strict accordance with the reagent instructions. The optical density (OD) values were measured at 450 nm.

### Flow cytometry

Flow cytometry was used to detect cell apoptosis by FITC-Annexin V Apoptosis Detection System (BD Bioscience) according to the manufacturer’s instructions.

### Tumor xenotransplantation mouse model

HCCLM3-CCR7-KO or HCCLM3-Control tumor cells were harvested and suspended in serum-free medium. 5 × 10^6^ tumor cells of each type were injected into one nude mouse (BALB/c background) at the right flank subcutaneously. For treatment, tumor-bearing mice were randomly divided into 4 groups at 7^th^ day after operation. The mice in the Control group and the CCR7-KO group were gavaged daily with 200 µL of a 5% castor oil and 5% ethanol aqueous solution. The mice in the Control+Sorafenib group and the CCR7-KO+Sorafenib group were gavaged daily with 200 µL of a 5% castor oil and 5% ethanol aqueous solution containing Sorafenib (30 mg/kg). The mice were sacrificed on the 28^th^ day after operation.

### Orthotopic tumor mouse model

Hepa1-6 or Hepa1-6-luc-GFP cells were harvested and suspended in PBS with Matrigel (356234, Corning). 1×10^5^ tumor cells were injected into the liver of C57BL/6 mouse to establish orthotopic tumor model. The tumor-bearing mice were randomly divided into 2 or 4 groups and given: PBS or 0.1 mg/kg/day VEGF-C^65^ (H4225, Acro Biosystems), and isotype or anti-PD-1 (200 μg/mouse, twice/week, A2122, A2123, Selleck). For live imaging, the tumor-bearing mice were injected into D-Luciferin (150 mg/ml, 200 μl/mouse) before imaging. The live imaging was performed every 7 days by using IVIS Spectrum In Vivo Imaging system (PerkinElmer), and quantitatively analyzed by using Living Image software (PerkinElmer).

### Preparation, transcriptome sequencing and analyses of mouse samples

The tumor tissues were collected from the orthotopic HCC mouse models (*n* = 5). Total RNA extraction, RNA purification, reverse transcription, library preparation and transcriptome sequencing were conducted according to the manufacturer’s instructions (illumine, CA) at Majorbio Bio-pharm Biotechnology Co., Ltd. (Shanghai, China). The expression of each transcript was calculated by using the transcripts per million reads (TPM) method, Transcripts with |log2FC | >= 1 and *p* value <= 0.05 were considered to be differentially expressed genes. Gene Ontology (GO), Kyoto Encyclopedia of Genes and Genomes (KEGG) and Gene Set Enrichment Analysis (GSEA) enrichment analysis were used to identify the terms and pathways in which differentially expressed genes were significantly enriched, and they were performed by Goatools and KOBAS, respectively.

### The tube formation

The mLECs were incubated with 100 ng/ml VEGF-C (H4225, Acro Biosystems) for 48 h. For tube formation, 96-well plates were coated with Matrigel (356234; Corning) and polymerized at 37°C for 30 min. mLECs (3 × 10^5^ cells/well) were seeded in 96-well plates and incubated for 4 h at 37 °C in a humidified atmosphere containing 5% CO_2_. Images were captured using the microscope (EVOS M7000 system) and quantitatively analyzed by using Image J.

### Statistical analysis

Experimental data were presented as the mean ± SD of at least three independent experiments performed in duplicate. Statistical analyses were reported in the Figure Legends. Kaplan-Meier analysis with log-rank test was used to assess the overall survival. Unpaired two-tailed Student’s *t* test was used to determine the differences between two groups, and Tukey-Kramer multiple comparison test was applied for post hoc comparisons. The Pearson correlation test was used to assess the correlation between different molecules. *p* < 0.05 was considered significant. All analyses were performed using SPSS 27 software (SPSS, USA).

## Supplementary information

Supplementary Materials-for SIGTRANS-16557R1

Supplementary TMA Data 1-for SIGTRANS-16557R1

Supplementary TMA Data 2-for SIGTRANS-16557R1

Supplementary materials for original images of western blot

06-16-Authorship form with signatures

## Data Availability

Spatial transcriptomics and single cell RNA sequence data from Figs. 1d, e and 3i-l are available from the National Genomics Data Center (OMIX001489, https://ngdc.cncb.ac.cn/omix/release/OMIX001489). RNA sequence data from the orthotopic HCC mouse models have been deposited at the Genome Sequence Archive at the National Genomics Data Center (CRA026302, https://ngdc.cncb.ac.cn/gsa/search?searchTerm=CRA026302). The clinical information from HCC tissue microarrays are available in Supplementary TMA data 1 and Supplementary TMA data 2 in the Supplementary Materials. All original and uncropped films of Western blots with labeling of molecular weight, sample names and corresponding figures were provided in the Supplementary Materials.
