# Supplementary material for: Differential expression pattern of CC chemokine receptor 7 guides precision treatment of hepatocellular carcinoma
Source: Signal Transduct Target Ther. 2025 Jul 21;10:229. doi: 10.1038/s41392-025-02308-6 (PMC12277428; doi:10.1038/s41392-025-02308-6)
Supplement: Supplementary file 1 — Supplementary Materials-for SIGTRANS-16557R1 [file 41392_2025_2308_MOESM1_ESM.docx]

Supplementary Materials for

**Differential expression pattern of CC chemokine receptor 7 guides precision treatment of hepatocellular carcinoma**

**Running title:** The Effect of CCL21/CCR7 axis in Tumor Microenvironment

Jie Qin^1†^, Qianyi Gong^1†^, Cheng Zhou^2†^, Jietian Xu^1,3†^, Yifei Cheng^2†^, Weiyue Xu^1^, Di Zhu^1^, Yiming Liu^1^, Yuye Zhang^1^, Yanru Wang^1^, Lingling Gao^1^, Lanfang Li^1^, Wulei Hou^1^, Qian Li^1^, Binbin Liu^2^, Yazhen Zhu^4^, Zuoyun Wang^1^, Jieyi Shi^2*^, Shuangjian Qiu^2*^, Chunmin Liang^1,2,3*^

* Correspondence to: cmliang@fudan.edu.cn (Chunmin Liang);

qiu.shuangjian@zs-hospital.sh.cn (Shuangjian Qiu);

shi.jieyi@zs-hospital.sh.cn (Jieyi Shi);

† These authors have contributed equally to this work and share first authorship

**This file includes:** Figures S1 to S18

Table S1 to S9

**Other Supplementary Materials for this manuscript include the following:**

All original and uncropped films of western blots (separate file)

**Supplemental figures**


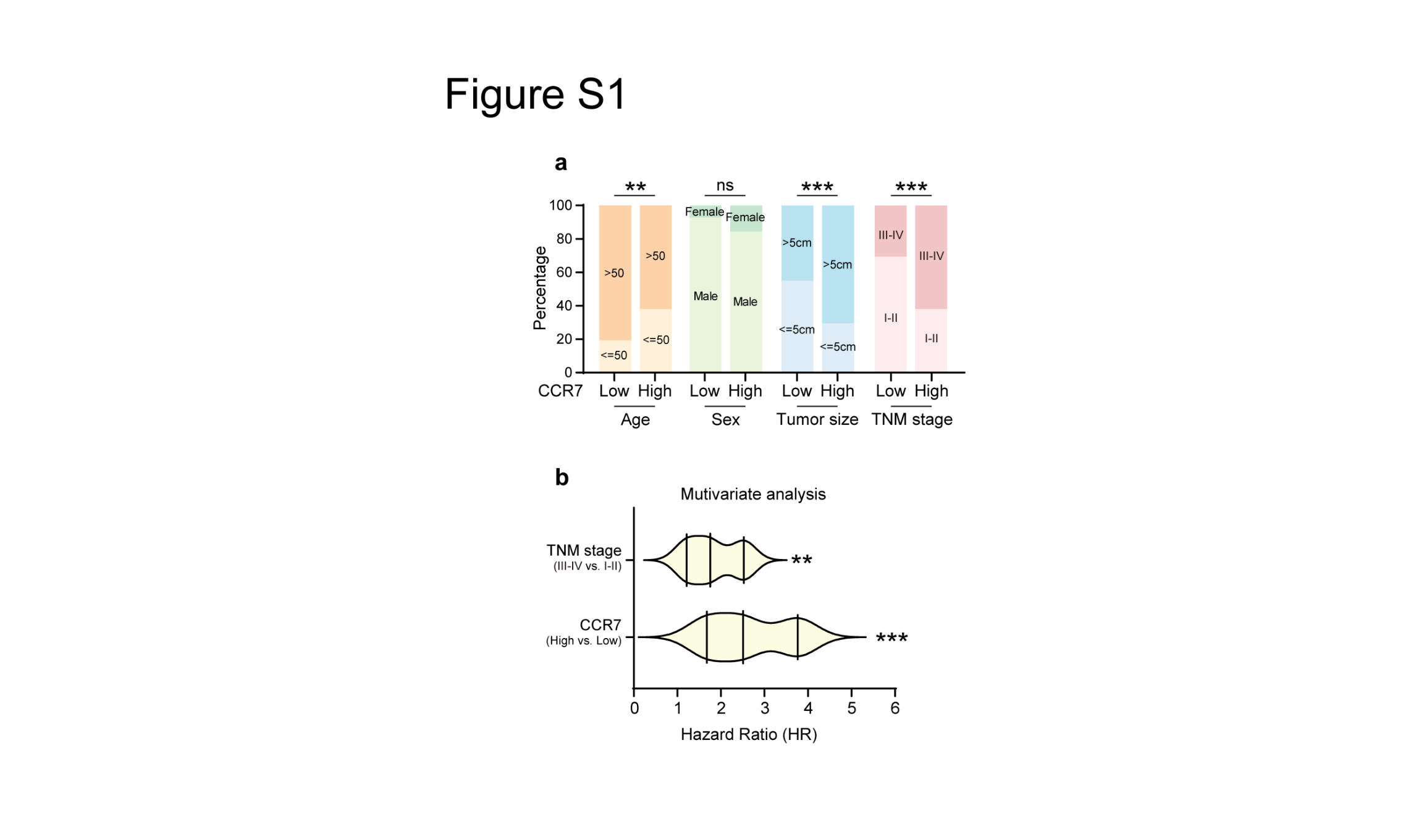


**Fig. S1 The CCR7 expression on the HCC TMA.**

**a** Correlations between CCR7 expression and clinical characteristics of patients with HCC from TMA. **b** Multivariate analysis of factors associated with overall survival of HCC patients from TMA (n = 240). Data in Fig. S1 were conducted in the same batch, n=240.


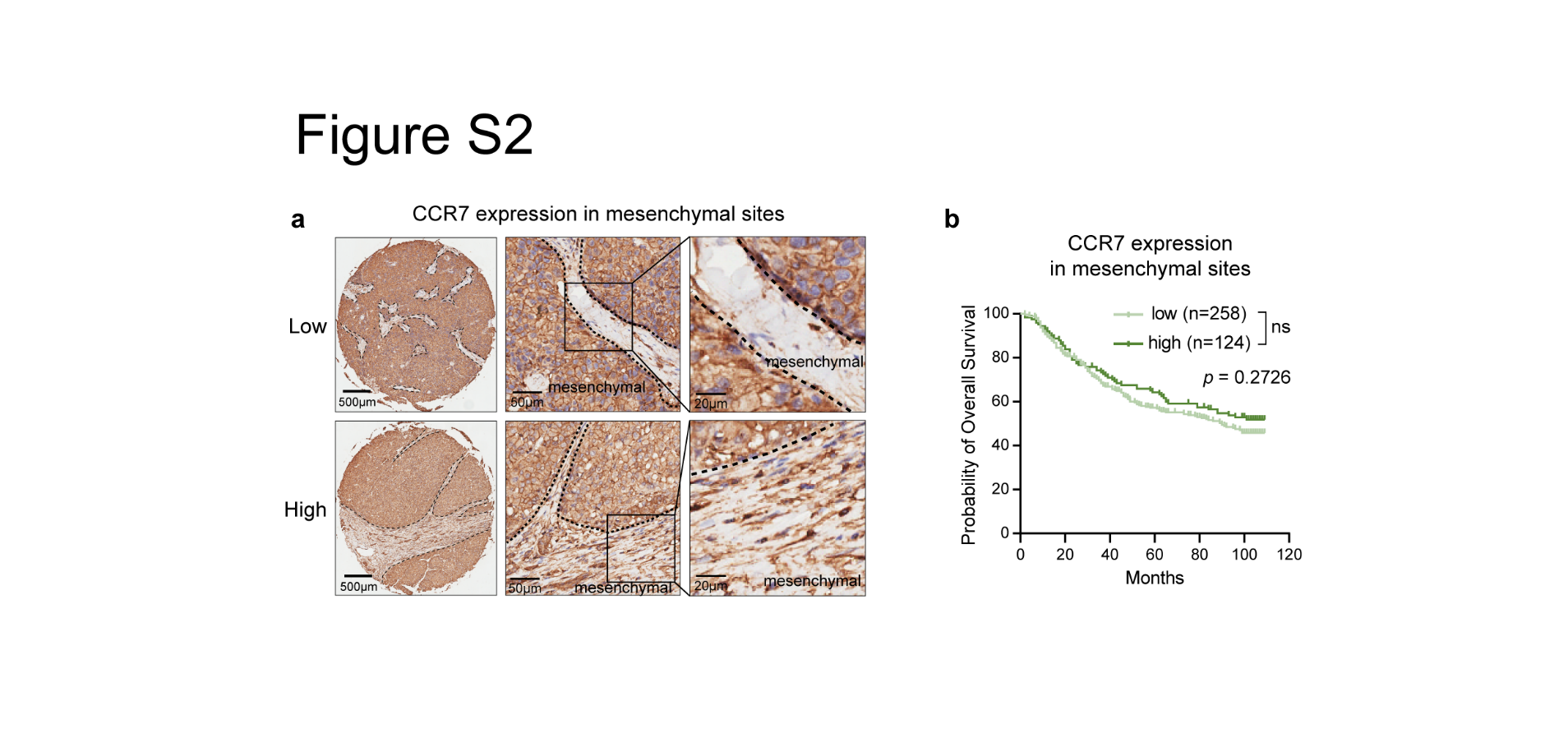


**Fig. S2 The CCR7 expression in mesenchymal sites analyzed from HCC TMA.**

**a** The representative image of low (top) and high (bottom) CCR7 expression in mesenchymal sites (outlined with dashed lines) by IHC staining. Scale bar (left): 500μm. Scale bar (middle): 50μm. Scale bar (right): 20μm. **b** Kaplan-Meier analyses of OS based on CCR7 in mesenchymal sites from HCC TMA, n=382. *p* value by two-sided log-rank test. *p*<0.05 is considered statistically significant.


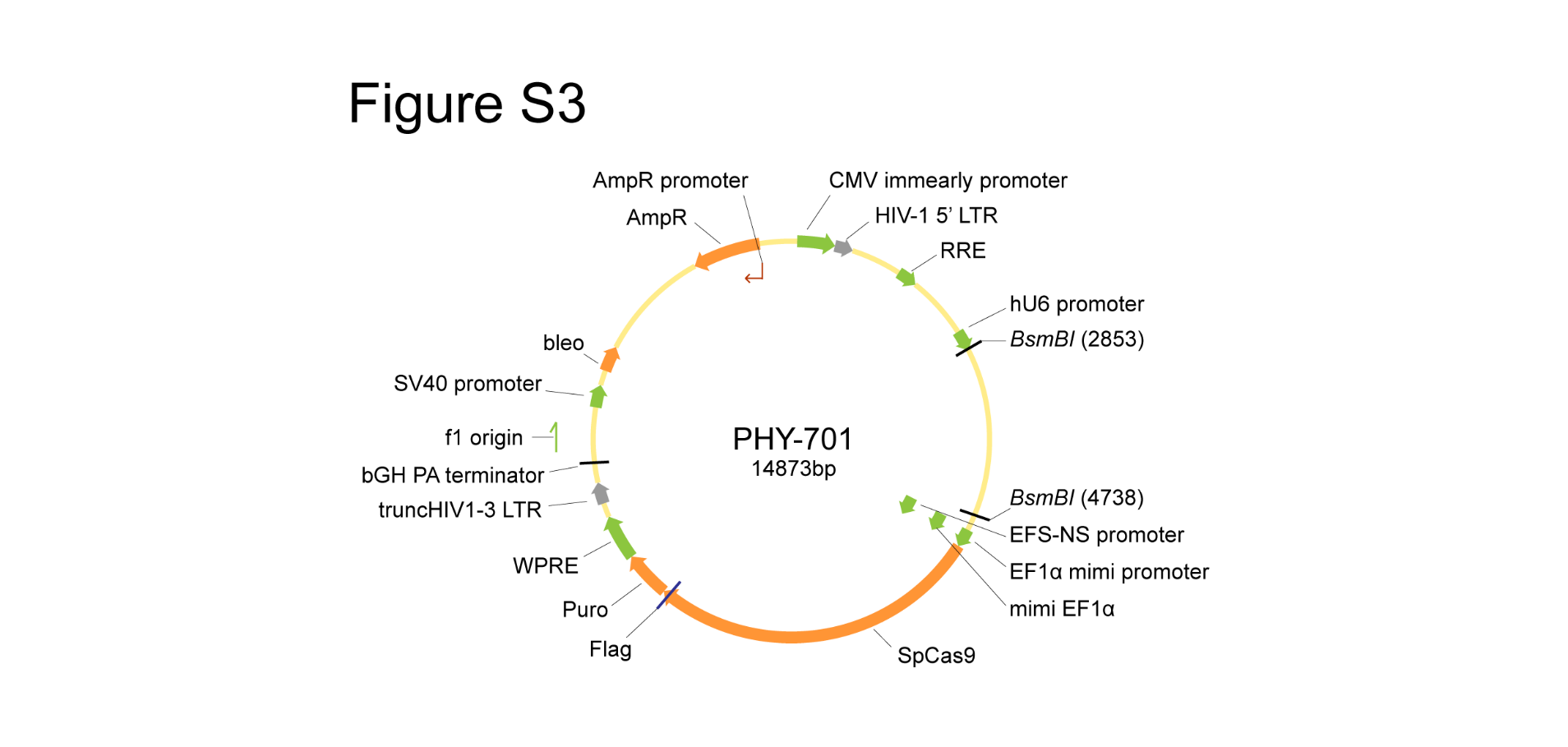


**Fig. S3 The structure diagram of Cas9 plasmid to establish CCR7 knockout HCC cells.**

The plasmid for CCR7 knockout based on CRISPR/Cas9 gene-editing system was used to transfect into HCCLM3 cells with original higher CCR7 expression.


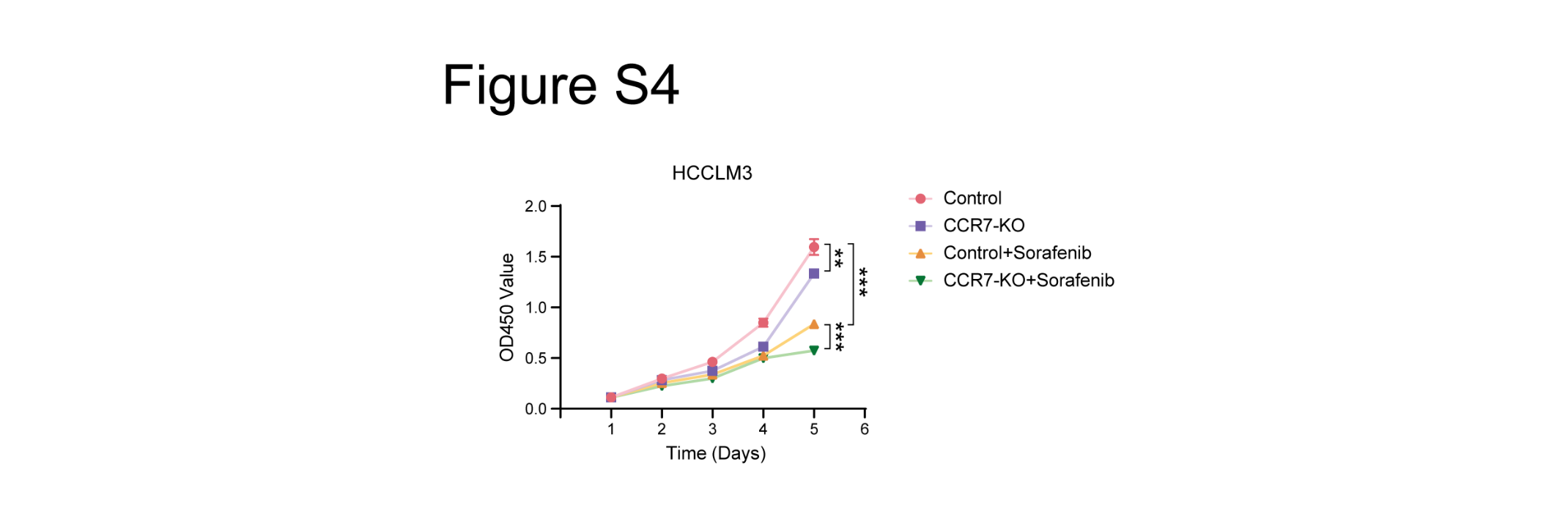


**Fig. S4 The effect of sorafenib treatment on cell proliferation in HCCLM3.**

After treating HCCLM3-Control and HCCLM3-CCR7-KO cells with 10µM sorafenib for 1 day, 2 days, 3 days, 4 days, 5 days, respectively. The cell proliferation was detected by CCK-8. ***p*<0.01, ****p*<0.001 by unpaired two-tailed Student’s *t* test. Data are represented as mean ± SD.


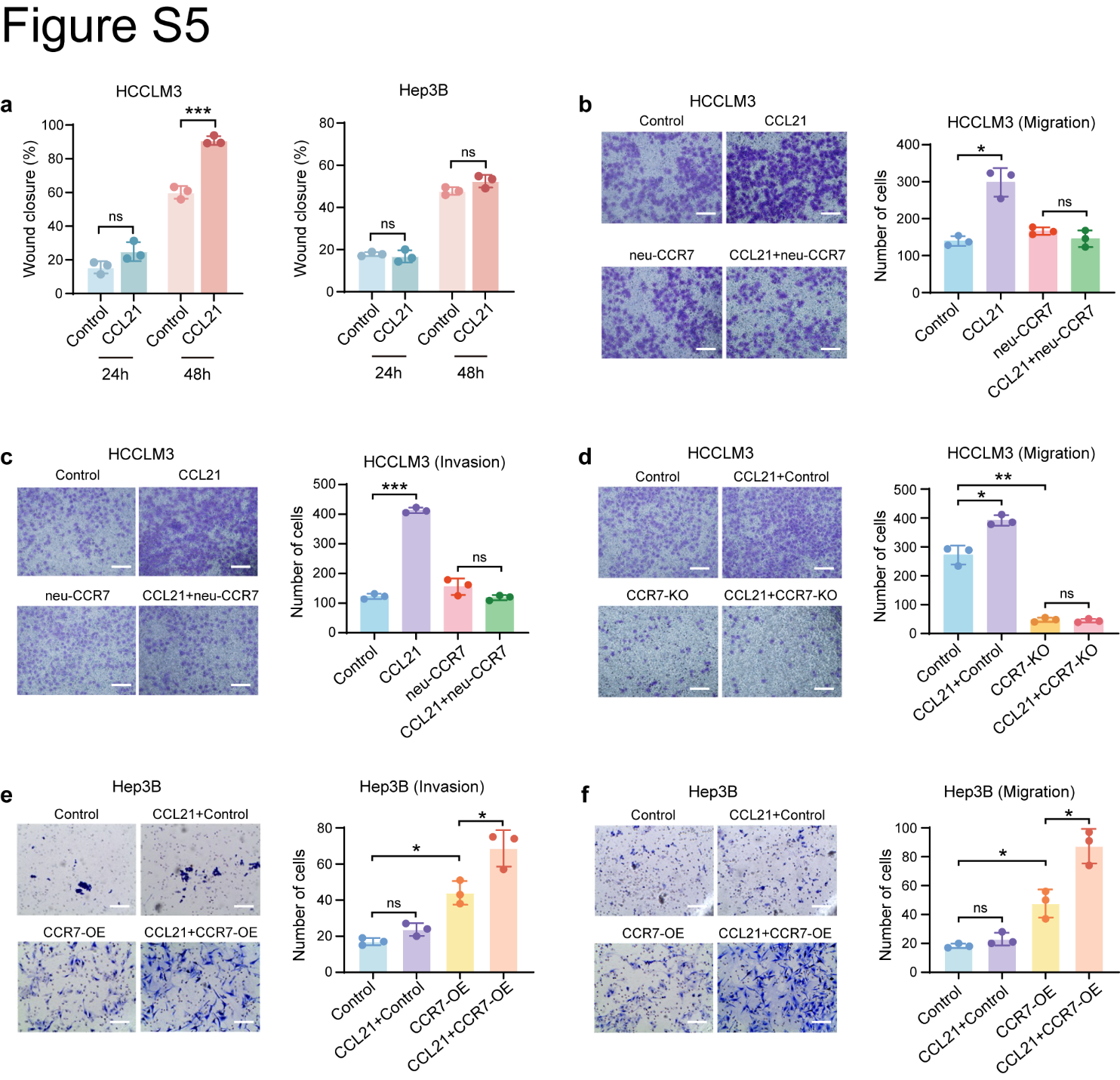


**Fig. S5 CCL21/CCR7 activation induced EMT in HCC cell lines.**

**a** The wound healing ability of HCCLM3 (left) and Hep3B (right) was detected after CCL21 stimulation. **b** The migration of HCCLM3 was detected by trans-well assay after blockage using the CCR7-specific neutralizing antibody (neu-CCR7) (left). Scale bar: 100μm. Quantitative analysis by using Image J (right). **c** The invasion of HCCLM3 was detected by trans-well assay after using neu-CCR7 (left). Scale bar: 100μm. Quantitative analysis by using Image J (right). **d** The migration ability of HCCLM3-KO was detected by trans-well assay after CCL21 stimulation (left). Scale bar: 100μm. Quantitative analysis by using Image J (right). **e** The invasion ability of Hep3B was detected by trans-well after CCL21 stimulation (left). Scale bar: 100μm. Quantitative analysis by using Image J (right). **f** The migration ability of Hep3B was detected by trans-well after CCL21 stimulation (left). Scale bar: 100μm. Quantitative analysis by using Image J (right). **p*<0.05, ***p*<0.01, ****p*<0.001 by unpaired two-tailed Student’s *t* test. Data are represented as mean ± SD with three independent replicates.


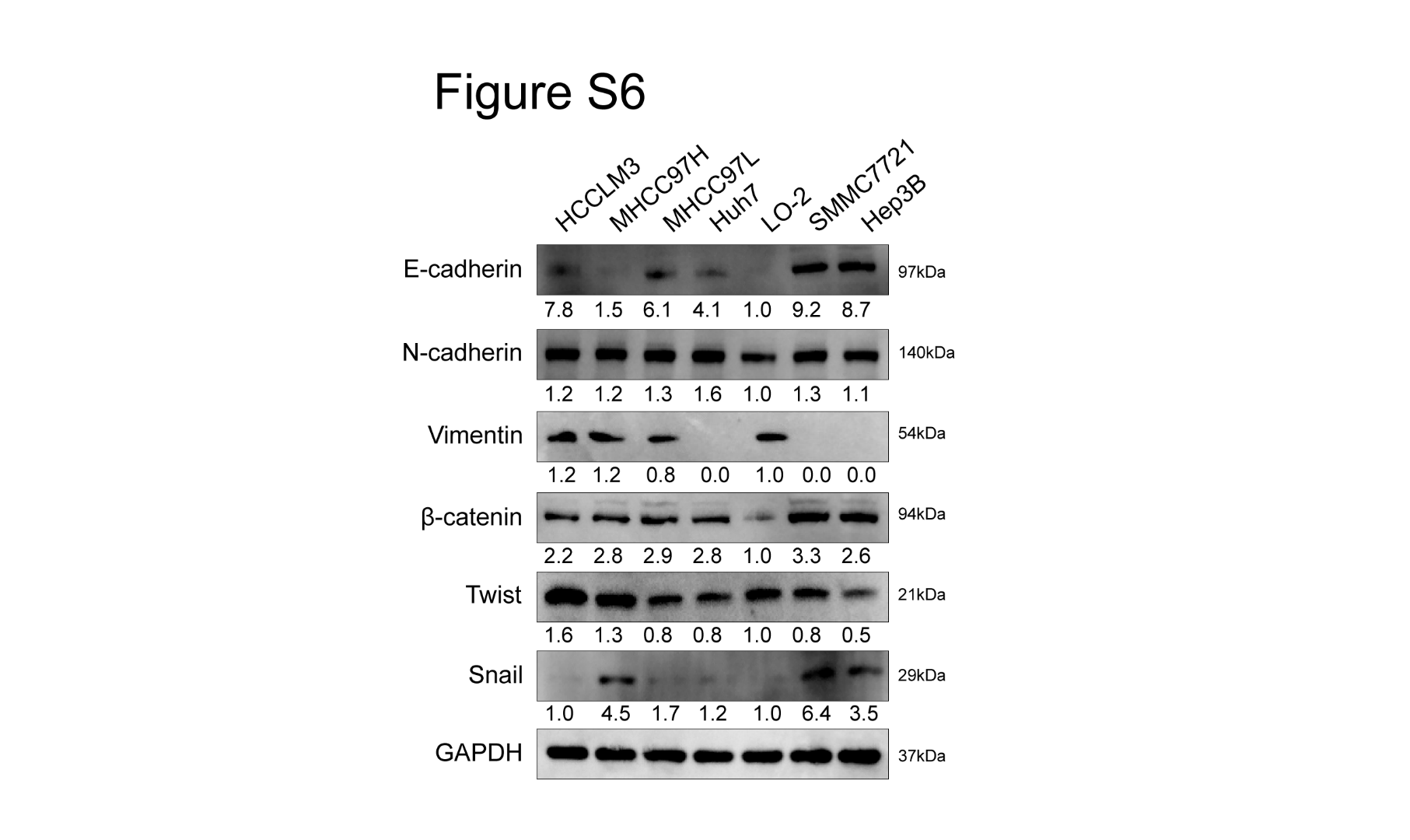


**Fig. S6 The expression of EMT-related markers was detected by western blot.**

The expression of EMT-related markers (E-cadherin, N-cadherin, Vimentin, β-catenin, Twist and Snail) in 6 human HCC tumor cell lines (HCCLM3, MHCC97H, MHCC97L, Huh7, SMMC7721 and Hep3B) and 1 normal human hepatocyte line (LO2) was detected by western blot.


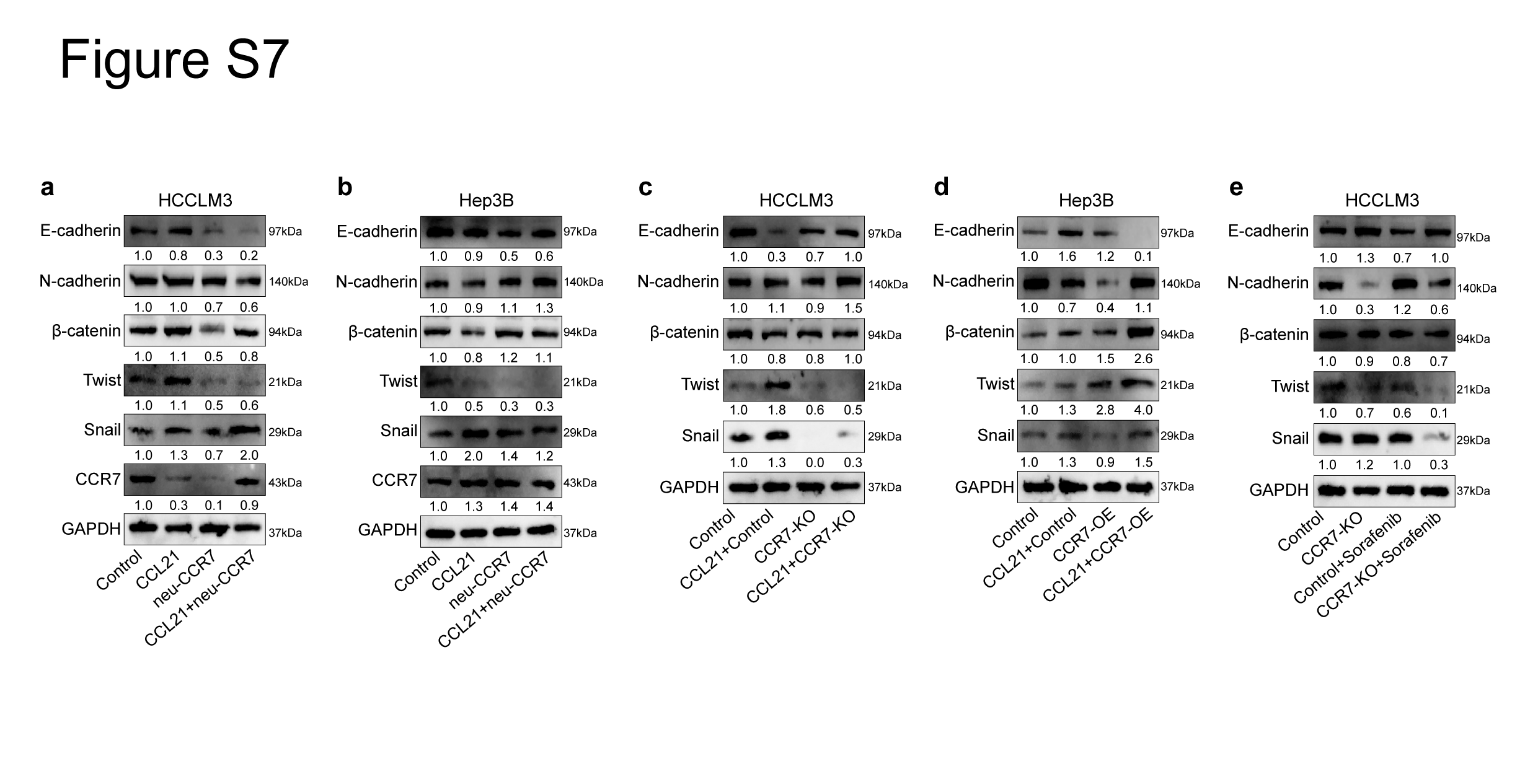


**Fig. S7 The expression of EMT-related markers on HCC cell lines using western blot.**

**a**-**b** The expression of EMT-related marker in HCCLM3 (**a**) and Hep3B (**b**) was detected after CCL21 stimulation, and blockage using the CCR7-specific neutralizing antibody (neu-CCR7). **c**-**d** The expression of EMT-related marker in HCCLM3-CCR7-KO (**c**) and Hep3B-CCR7-OE (**d**) was detected after CCL21 stimulation. **e** After treating HCCLM3-Control and HCCLM3-CCR7-KO cells with 10µM sorafenib for 48 hours, the cells were collected and protein samples were prepared. Western blot analysis was performed to detect the expression of EMT-related markers.


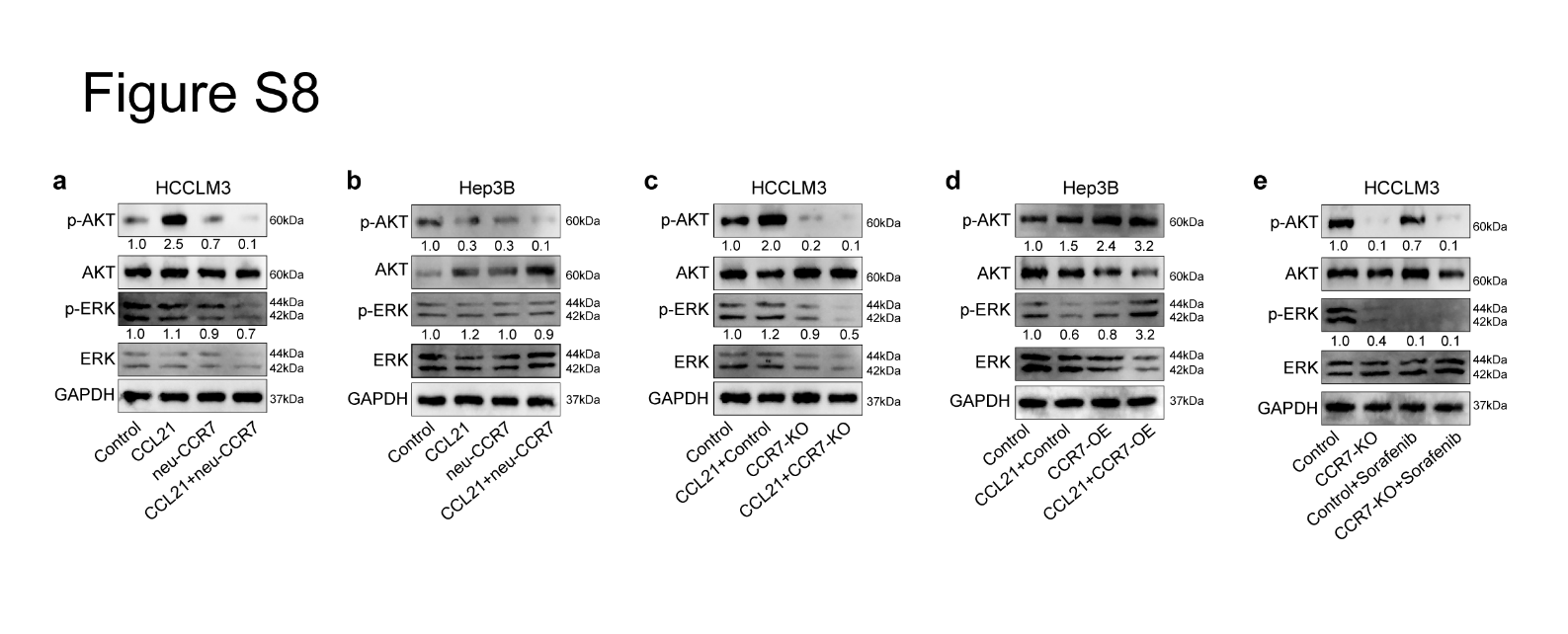


**Fig. S8 The activation of signal pathway in HCCLM3 and Hep3B using Western Blot.**

**a**-**b** The signal pathways including AKT and ERK in HCCLM3 (**a**) and Hep3B (**b**) were detected after CCL21 stimulation, and blockage using the CCR7-specific neutralizing antibody (neu-CCR7). **c**-**d** The signal pathways including AKT and ERK in HCCLM3-CCR7-KO (**c**) and Hep3B-CCR7-OE (**d**) was detected after CCL21 stimulation. **e** After treating HCCLM3-Control and HCCLM3-CCR7-KO cells with 10µM sorafenib for 48 hours, the cells were collected and protein samples were prepared. Western blot analysis was performed to detect the signal pathways including AKT and ERK.


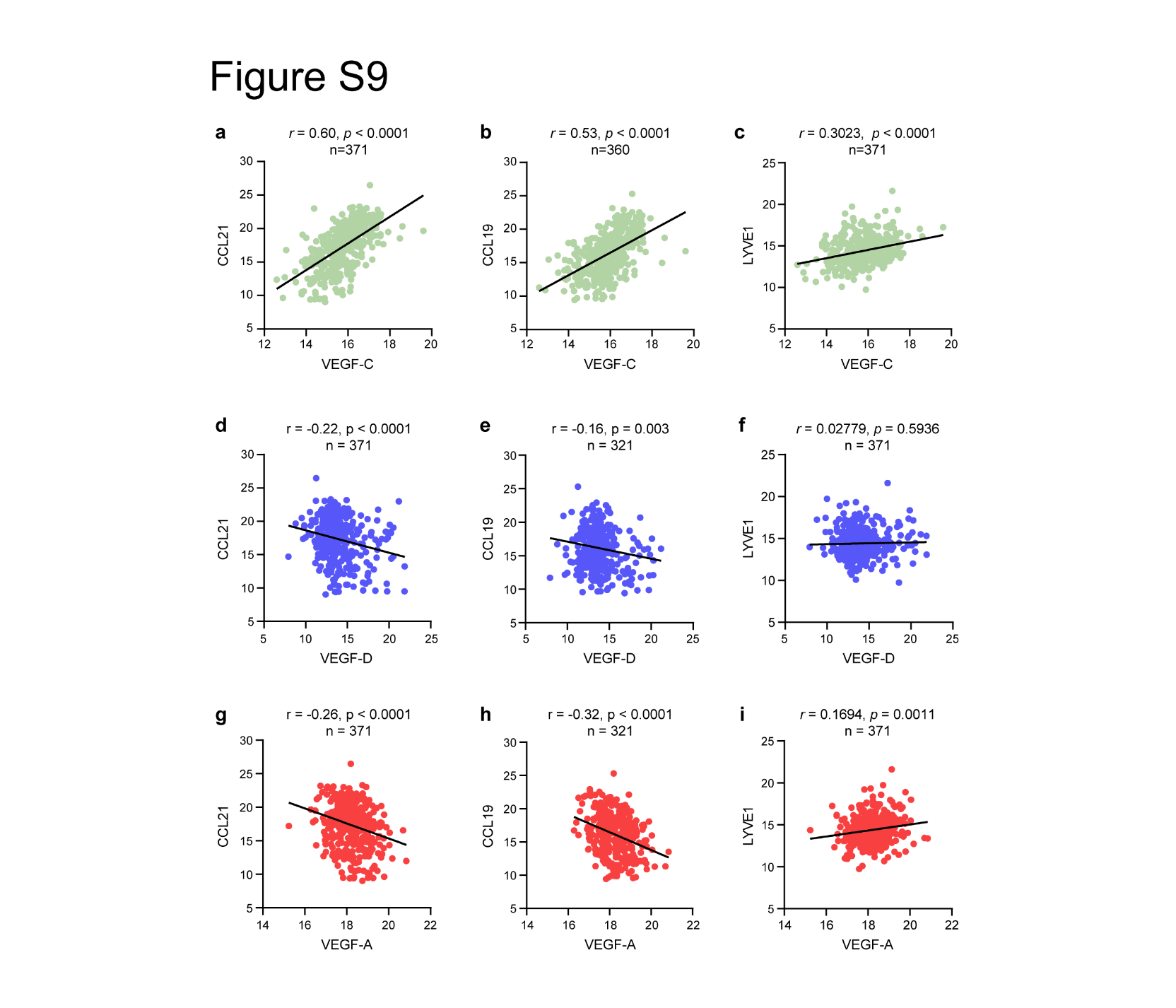


**Fig. S9 The analysis on HCC patients from TCGA.**

**a** The correlation between VEGF-C and CCL21 from TCGA (n=371). **b** The correlation between VEGF-C and CCL19 from TCGA (n=360). **c** The correlation between VEGF-C and LYVE1 from TCGA (n=371). **d** The correlation between VEGF-D and CCL21 from TCGA (n=371). **e** The correlation between VEGF-D and CCL19 from TCGA (n=321). **f** The correlation between VEGF-D and LYVE1 from TCGA (n=371). **g** The correlation between VEGF-A and CCL21 from TCGA (n=371). **h** The correlation between VEGF-A and CCL19 from TCGA (n=321). **i** The correlation between VEGF-A and LYVE1 from TCGA (n=371). *r* value and *p* value by Pearson correlation test in Fig. S9. *p*<0.05 is considered statistically significant.


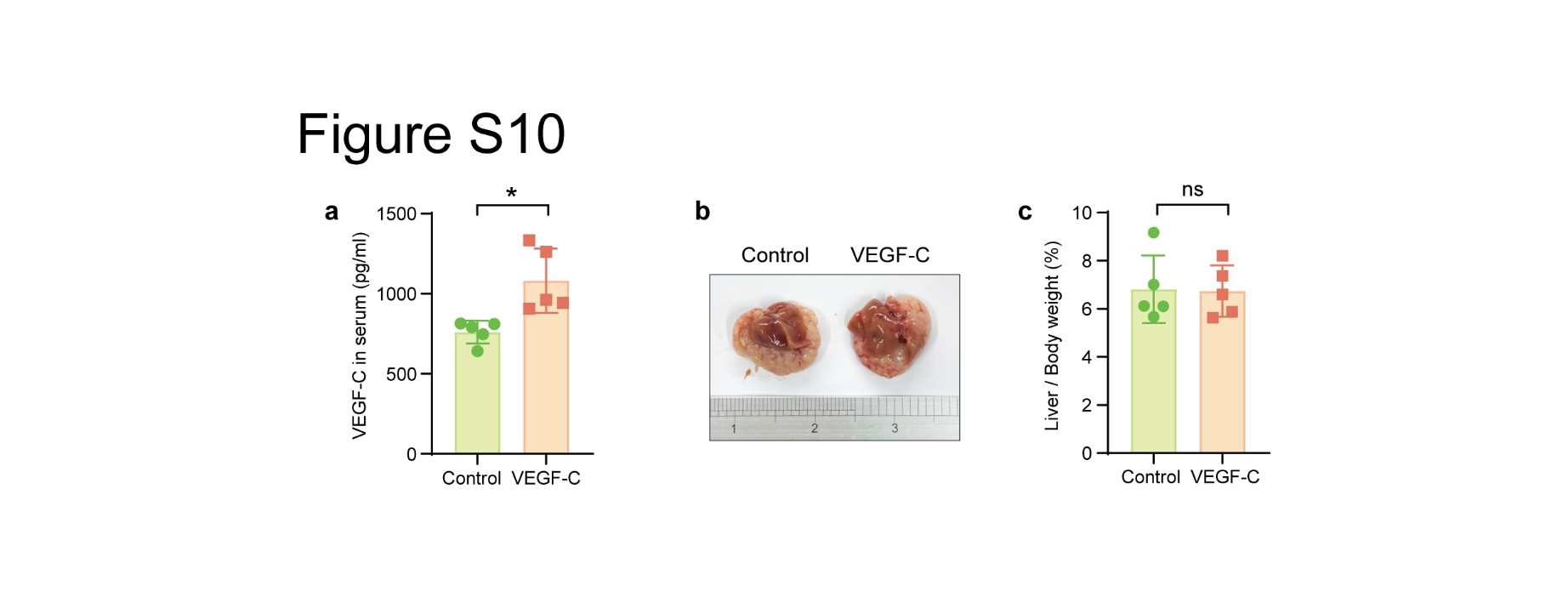


**Fig. S10 The effect of systemic VEGF-C administration in the orthotopic HCC mouse model.**

**a** The concentration of VEGF-C in the serum of mice treated with VEGF-C was detected by ELISA. **b** Representative images of tumor-bearing liver from the orthotopic HCC mouse model. **c** The ratio of the tumor-bearing liver weight to body weight in mice was analyzed. n=5. *ns*>0.05 by unpaired two-tailed Student’s *t* test. Data are represented as mean ± SD.

**
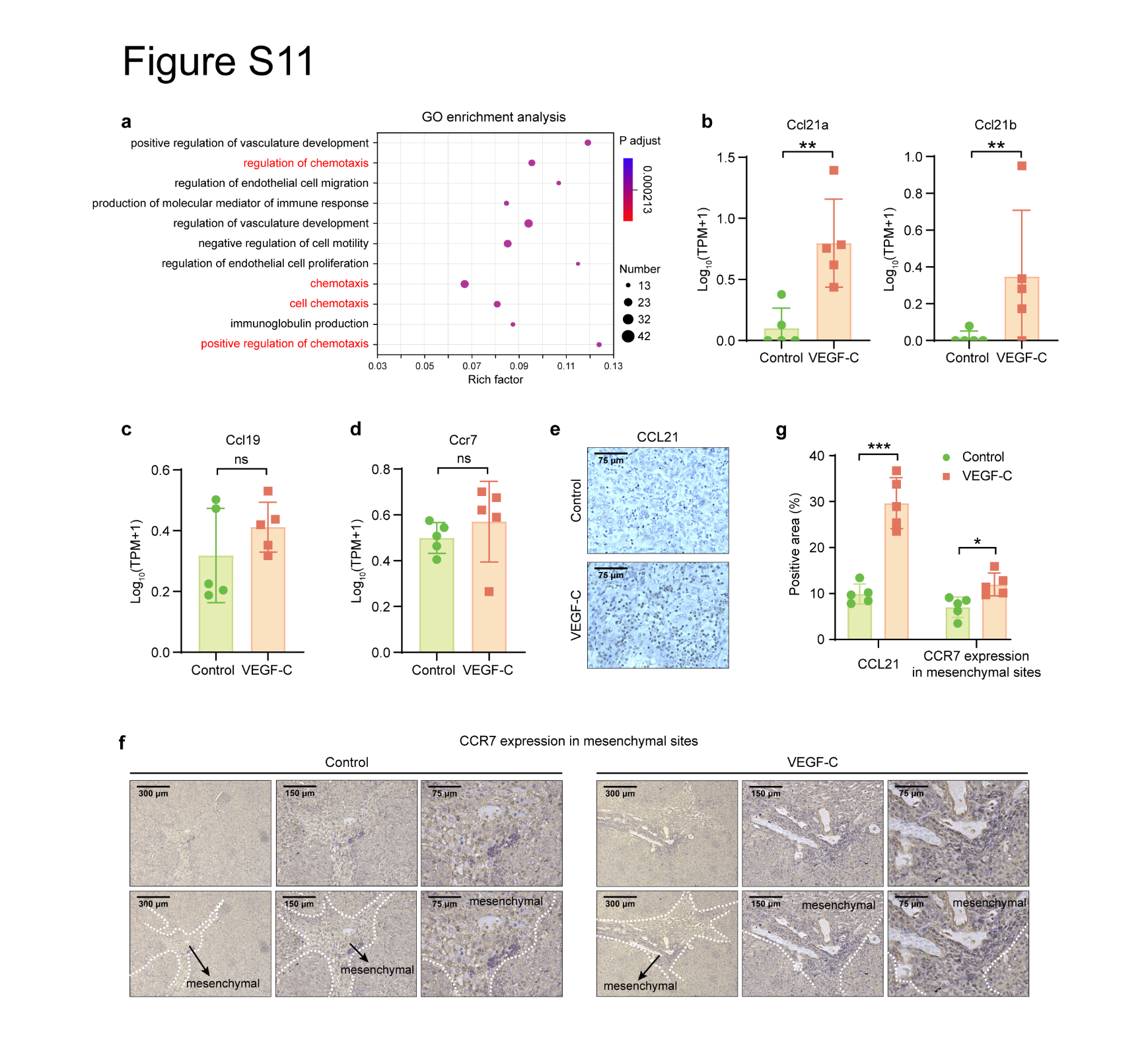
**

**Fig. S11 VEGF-C promoted chemokine *in vivo*.**

**a** The GO enrichment analysis of differential expression genes in tumor from the orthotopic HCC mouse model. **b** The expression of CCL21 genes (Ccl21a (left) and Ccl21b (right)) in tumor from the orthotopic HCC mouse model. **c** The expression of Ccl19 genes in tumor from the orthotopic HCC mouse model. **d** The expression of Ccr7 in tumor from the orthotopic HCC mouse model. **e** The representative images of CCL21 expression in tumor from the orthotopic HCC mouse model. **f** The representative images of CCR7 expression in tumor from the orthotopic HCC mouse model. **g** Quantitative analysis of (**e**) and (**f**) by Image J. *ns*>0.05, **p*<0.05, ***p*<0.01, ****p*<0.001 by unpaired two-tailed Student’s *t* test. Data are represented as mean ± SD.


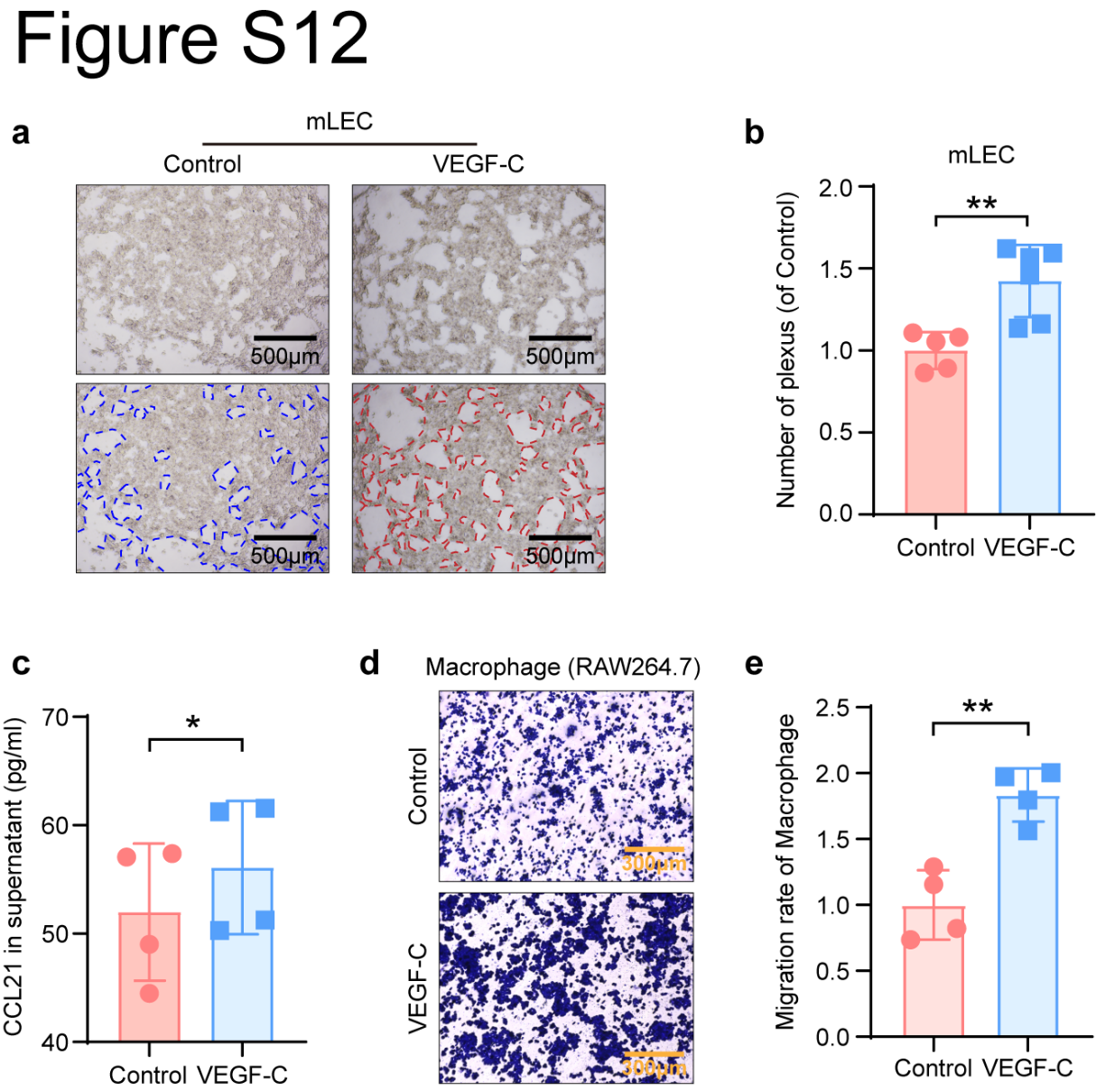


**Fig. S12 VEGF-C promoted chemokine *in vitro*.**

**a** The tube formation of mLECs was observed after the incubation of VEGF-C *in vitro*. **b** Quantitative analysis of (**a**) by Image J. **c** The CCL21 concentration in the supernatant of mLECs culture was detected by ELISA. **d** The migration ability of RAW264.7 was detected by trans-well assay after the incubation of the supernatant of mLECs treated with VEGF-C. **e** Quantitative analysis of (**d**) by Image J. *ns*>0.05, **p*<0.05, ***p*<0.01, ****p*<0.001 by unpaired two-tailed Student’s *t* test. Data are represented as mean ± SD.

~~
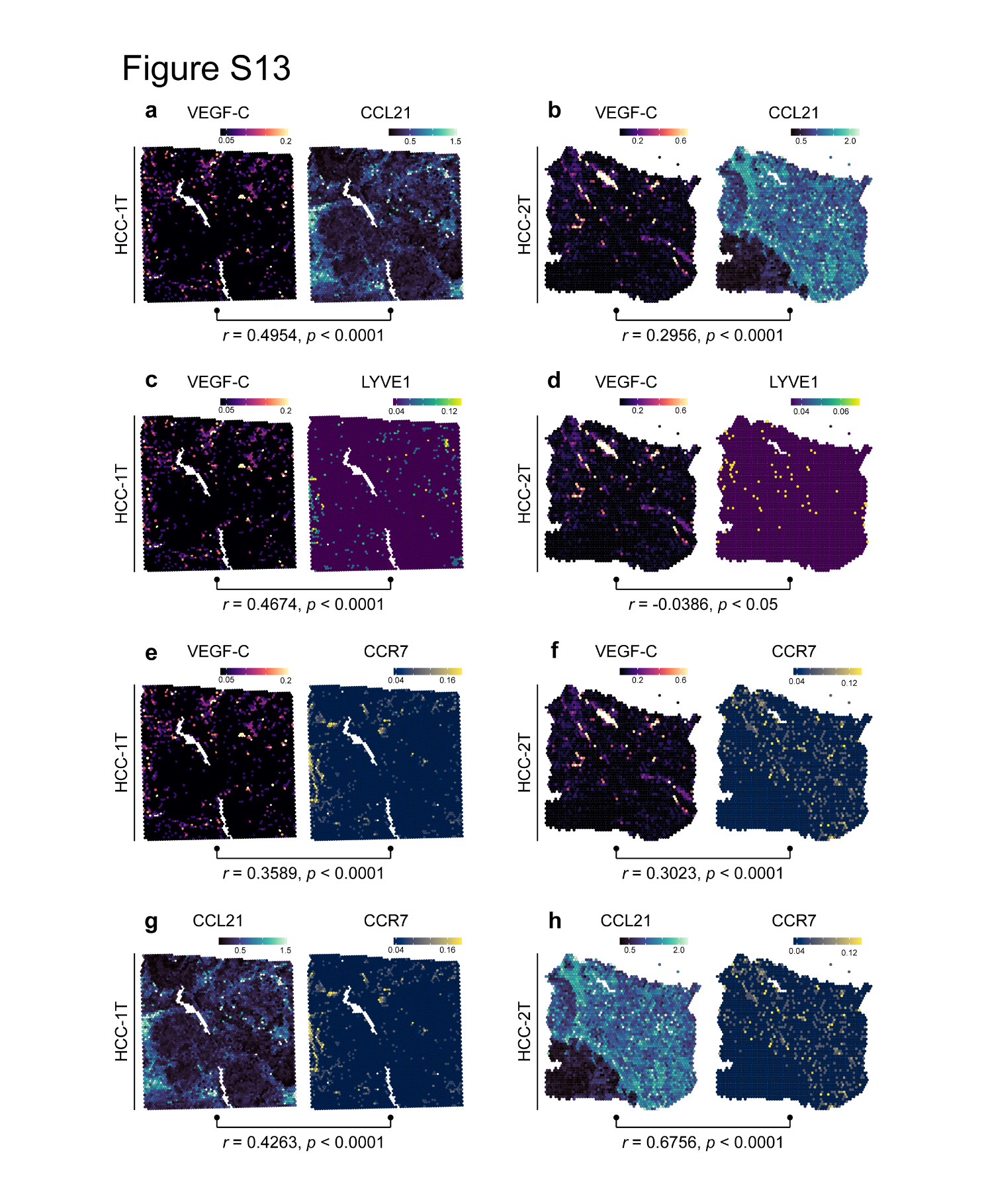
~~

**Fig. S13 Spatial transcriptomic analysis of clinical HCC tissue slides (HCC-1T and HCC-2T).**

**a**-**b** Spatial distribution of VEGF-C and CCL21 expression. **c**-**d** Spatial distribution of VEGF-C and LYVE1 expression. **e**-**f** Spatial distribution of VEGF-C and CCR7 expression. **g**-**h** Spatial distribution of CCL21 and CCR7 expression. The correlation was analyzed using Pearson analysis. *p*<0.05 is considered statistically significant.


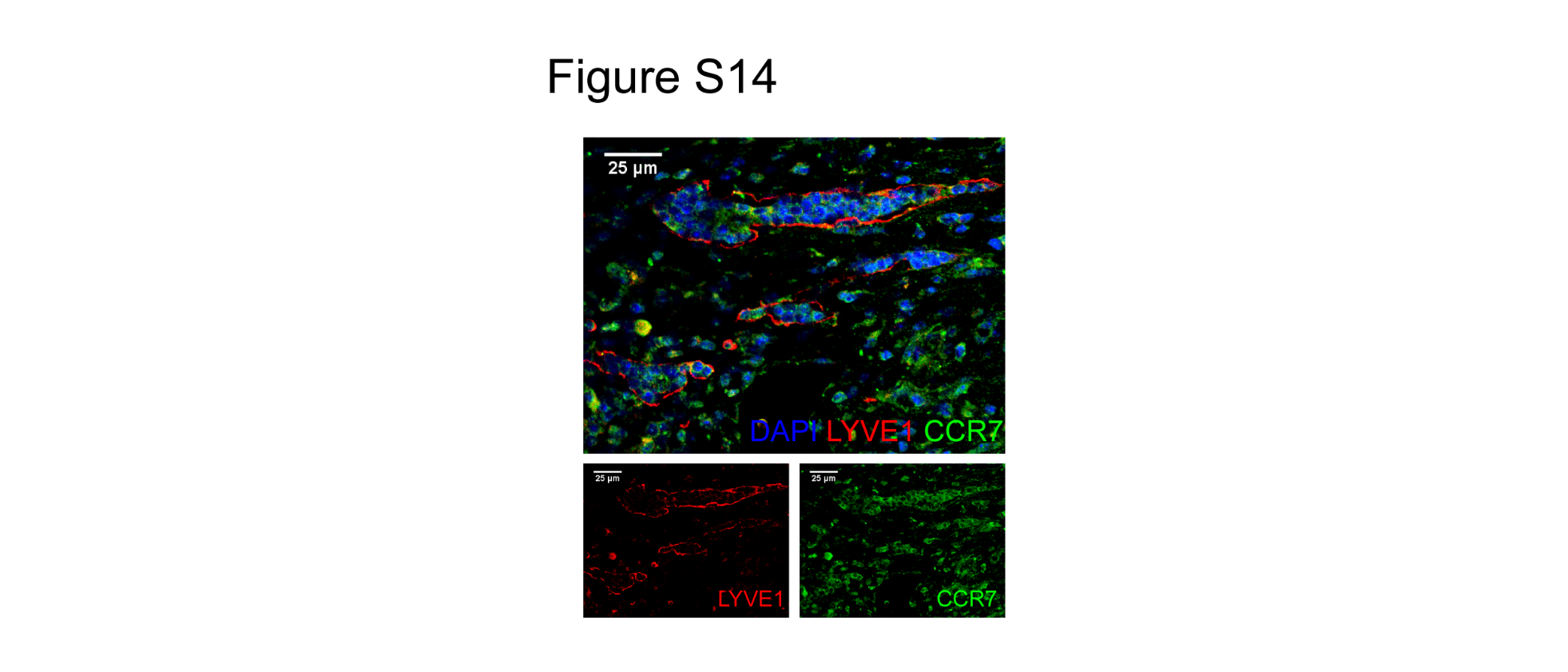


**Fig. S14 Immunofluorescence staining of LYVE1 and CCR7 in tumor tissues from orthotopic HCC mouse model.**

CCR7⁺ cells migrated into the tumor through the lymphatic vessels after VEGF-C administration.


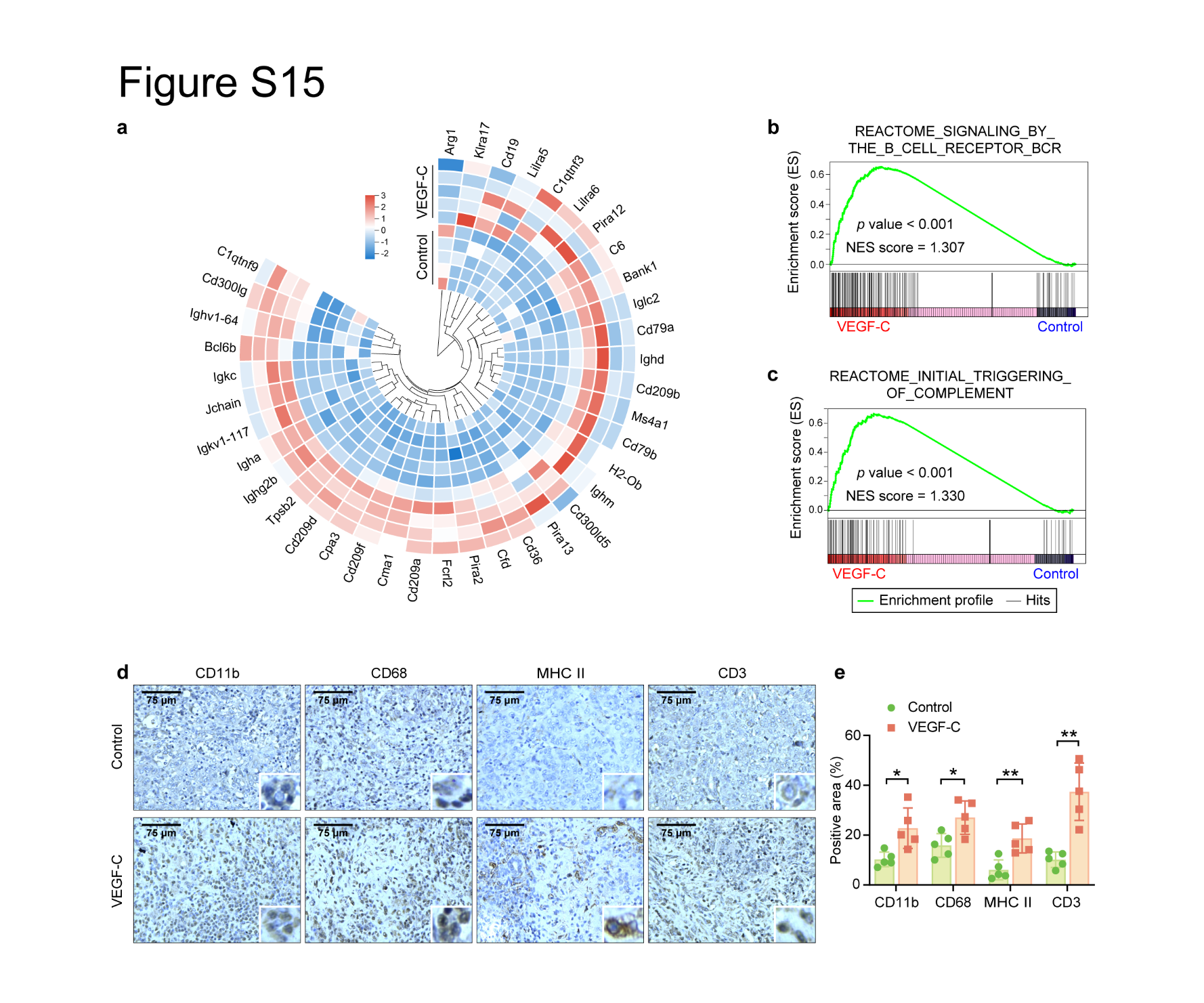


**Fig. S15 VEGF-C promoted the infiltration of immune cells in the orthotopic tumor mouse model.**

**a** The gene expression heatmap of immune-related factors from transcriptome sequencing in the orthotopic HCC mouse model. **b**-**c** GSEA analysis on gene pathways, including SIGNALING_BY_THE_B_CELL_RECEPTOR_BCR (**b**) and INITIAL_TRIGGERING_OF_ COMPLEMENT (**c**). **d** The representative images of CD11b, CD68, MHC II, and CD3 expression by IHC staining in tumor sites. **e** Quantitative analysis of (**d**) by Image J. **p*<0.05, ***p*<0.01 by unpaired two-tailed Student’s *t* test. Data are represented as mean ± SD.


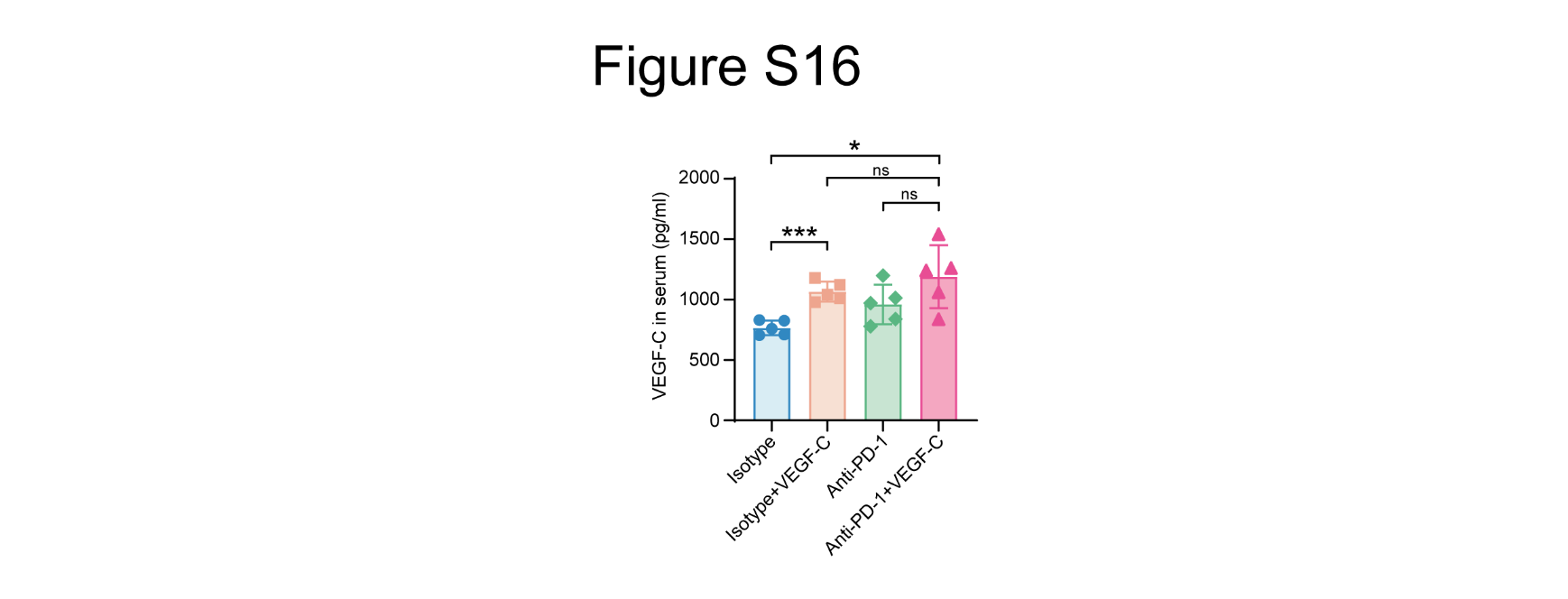


**Fig. S16 The concentration of VEGF-C in the serum from mice treated with VEGF-C and Anti-PD-1.**

The VEGF-C concentration was detected by ELISA in the Isotype, Isotype + VEGF-C, Anti-PD-1, Anti-PD-1+VEGF-C groups. **p*<0.05, ***p*<0.01, ****p*<0.001 by unpaired two-tailed Student’s *t* test. Data are represented as mean ± SD.


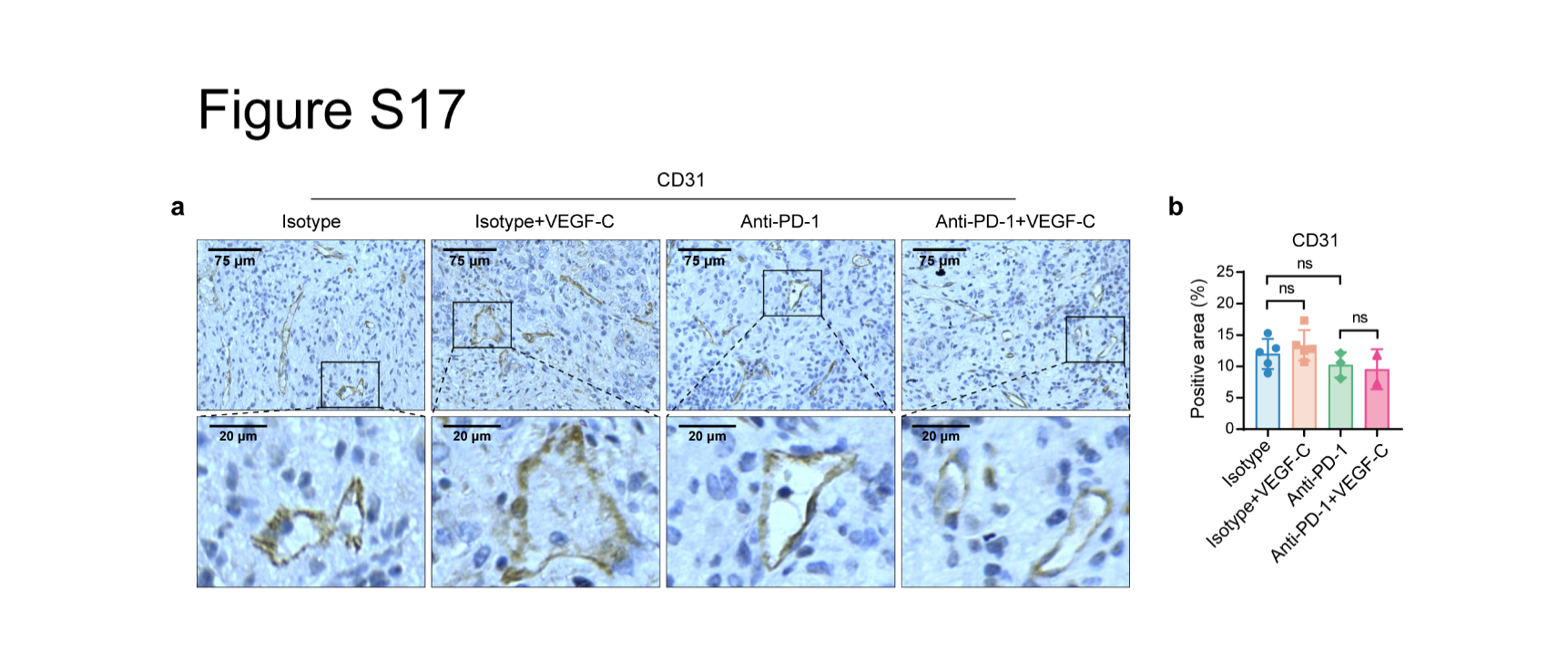


**Fig. S17 VEGF-C did not promote the formation of blood vessels in the orthotopic tumor mouse model.**

**a** The representative images of CD31 expression in tumor. **b** Quantitative analysis of CD31 by Image J. *ns*>0.05 by unpaired two-tailed Student’s *t* test. Data are represented as mean ± SD.

**
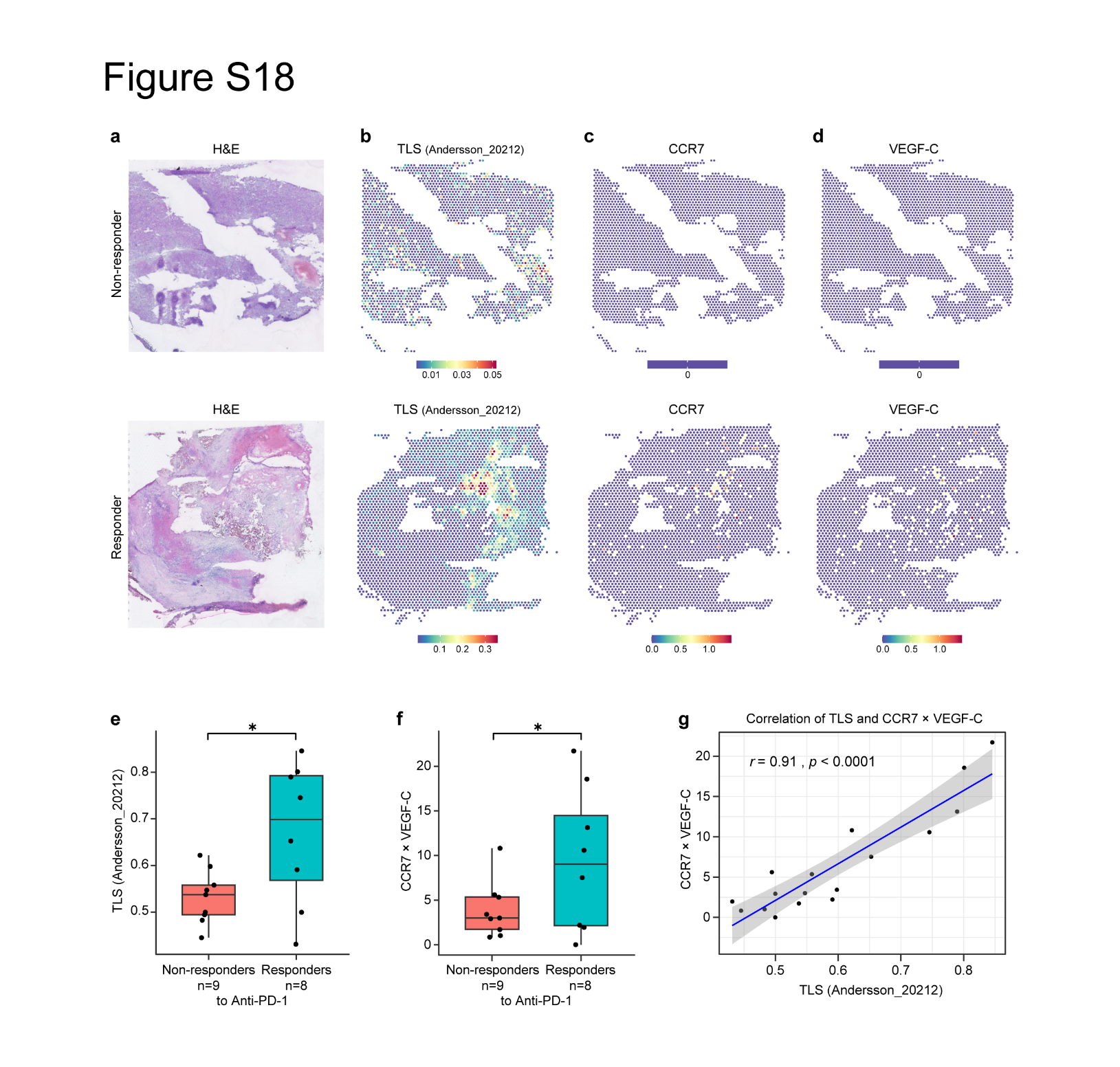
**

**Fig. S18 The relationships among tertiary lymphoid structures (TLSs), CCR7, VEGF-C in tumor tissues from clinical HCC patients treated with immunotherapy.**

**a**-**d** Top line: Non-responder to Anti-PD-1 immunotherapy. Bottom line: Responder to Anti-PD-1 immunotherapy. **a** Hematoxylin and eosin (H&E) staining of clinical HCC tissue slides. **b** Spatial distribution of TLS-related genes. **c** Spatial distribution of CCR7 expression. **d** Spatial distribution of VEGF-C expression. **e** The expression of TLS-related genes in tumor tissues from Non-responders and Responders to Anti-PD-1. **f** The expression of CCR7 × VEGF-C (a combined measure of CCR7 and VEGF-C expression) in tumor tissues from Non-responders and Responders to Anti-PD-1. **g** The correlation between TLSs and CCR7 × VEGF-C expression. The correlation was analyzed using Pearson analysis. *p*<0.05 is considered statistically significant.

**Supplementary Tables**

Table S1. **Correlations between CCR7 expression on tumor tissues and clinical characteristics in HCC patients (*n* = 240)**

| Characteristics | | CCR7 intensity | | *p* value |
| --- | --- | --- | --- | --- |
|  |  | Low | High |  |
| Age | <= 50 | 19 | 54 | 0.002 |
|  | > 50 | 79 | 88 |  |
| Sex | Male | 91 | 120 | 0.051 |
|  | Female | 7 | 22 |  |
| Tumor size | <= 5cm | 54 | 42 | < 0.001 |
|  | > 5cm | 44 | 100 |  |
| TNM stage | Ⅰ-Ⅱ | 68 | 54 | < 0.001 |
|  | Ⅲ-Ⅳ | 30 | 88 |  |

Table S2. **Univariate analyses of factors associated with OS (*n =*240)**

| Variables | | Univariate |
| --- | --- | --- |
|  |  | *p* value |
| Age | > 50 vs. <= 50 | 0.514 |
| Sex | Female vs. Male | 0.549 |
| Tumor size | > 5cm *vs*. <= 5cm | **0.002** |
| CCR7 expression | High *vs*. Low | **<0.001** |
| TNM stage | Ⅲ-Ⅳ *vs*. Ⅰ-Ⅱ | **<0.001** |

**Table S3. The correlation of Clinical characteristics and peritumor VEGF-C expression on HCC patients (n=382)**

| Variables | | Peritumor VEGF-C expression  No. (%) | | *p* value |
| --- | --- | --- | --- | --- |
|  |  | Low (n=178) | High (n=204) |  |
| Age (years) | <= 50 | 73 (41.0) | 75 (36.8) | 0.402 |
|  | > 50 | 105 (59.0) | 129 (63.2) |  |
| Gender | Male | 147 (82.6) | 173 (84.8) | 0.580 |
|  | Female | 31 (17.4) | 31 (15.2) |  |
| History of cirrhosis | No | 31 (17.4) | 50 (24.5) | 0.103 |
|  | Yes | 147 (82.6) | 154 (75.5) |  |
| Tumor size (cm) | <= 5 | 106 (59.6) | 127 (62.3) | 0.601 |
|  | > 5 | 72 (40.4) | 77 (37.7) |  |
| Tumor number | Single | 150 (84.3) | 176 (86.3) | 0.664 |
|  | Multiple | 28 (15.7) | 28 (13.7) |  |
| BCLC stage | 0-A | 69 (38.8) | 99 (48.5) | 0.063 |
|  | B-C | 109 (61.2) | 105 (51.5) |  |
| TNM stage | I-II | 95 (53.4) | 134 (65.7) | **0.016** |
|  | III-IV | 83 (46.6) | 70 (34.3) |  |
| Recurrence | No | 108 (60.7) | 110 (53.9) | 0.214 |
|  | Yes | 70 (39.3) | 94 (46.1) |  |

**Table S4. Univariate analysis of factors associated with OS (*n =* 382)**

| Variables | | Univariate analysis | | |
| --- | --- | --- | --- | --- |
|  |  | HR | 95%CI | *p* value |
| Age (years) | <= 50 vs. > 50 | 1.056 | 0.787-1.416 | 0.718 |
| Gender | male vs. female | 0.755 | 0.503-1.135 | 0.176 |
| History of cirrhosis | yes *vs*. no | 1.335 | 0.965-1.846 | 0.081 |
| Tumor size (cm) | <= 5 *vs*. > 5 | 1.872 | 1.411-2.485 | **0.000*** |
| Tumor number | multiple vs. single | 1.822 | 1.281-2.591 | **0.000*** |
| BCLC stage | 0-A vs B-C | 1.923 | 1.427-2.590 | **0.000*** |
| TNM stage | Ⅰ-Ⅱ *vs*. Ⅲ-Ⅳ | 1.906 | 1.437-2.530 | **0.000*** |
| Recurrence | yes *vs*. no | 4.367 | 3.208-5.944 | **0.000*** |
| Peritumor VEGF-C | low *vs*. high | 0.753 | 0.568-0.999 | **0.049*** |

**Table S5. Multivariate analysis of factors associated with OS (*n =* 382)**

| Variables | | Multivariate analysis | | |
| --- | --- | --- | --- | --- |
|  |  | HR | 95%CI | *p* value |
| Tumor number | multiple vs. single | 1.575 | 1.019-2.432 | **0.041*** |
| Recurrence | yes *vs*. no | 4.436 | 3.246-6.063 | **0.000*** |
| Peritumor VEGF-C | low *vs*. high | 0.662 | 0.495-0.884 | **0.005*** |

**Table S6. The primary and secondary antibodies**

| Name | Supplier | Cat No. | Clone No. |
| --- | --- | --- | --- |
| anti-β-catenin | abcam | ab16051 | N/A |
| anti-CCR7 | abcam | ab32527 | Y59 |
| anti-CXCR4 | abcam | ab124824 | UMB2 |
| anti-N-cadherin | CST | 4061S | N/A |
| anti-E-cadherin | abcam | ab76055 | M168 |
| anti-Twist | abcam | ab50887 | Twist2C1a |
| anti-Snail | CST | 3895S | L70G2 |
| anti-GAPDH | CST | 5174S | D16H11 |
| anti-p-Akt | CST | 4060S | D9E |
| anti-Akt | CST | 4685S | 11E7 |
| anti-p-Erk | CST | 4370S | D13.14.4E |
| anti-Erk | CST | 9102S | N/A |
| anti-p-p38MAPK | CST | 4511T | D3F9 |
| anti-p38MAPK | CST | 8690T | D13E1 |
| anti-p-P65 | abcam | ab86299 | N/A |
| anti-P65 | abcam | ab16502 | N/A |
| anti-cleaved-Caspase-9 | CST | 7237T | D2D4 |
| anti-cleaved-Caspase-3 | CST | 9661S | Asp175 |
| anti-cleaved-PARP | CST | 9541S | Asp214 |
| anti-Vimentin | abcam | ab92547 | EPR3776 |
| anti-VEGF-C | proteintech | 22601-1-AP | N/A |
| anti-CD31 | proteintech | 28083-1-AP | N/A |
| anti-LYVE1 | abcam | ab14917 | N/A |
| anti-CD11b | abcam | ab133357 | N/A |
| anti-CD68 | proteintech | 25747-1-AP | N/A |
| anti-MHC Ⅱ | proteintech | 17617-1-Ig | M5/114.15.2 |
| anti-CD3 | proteintech | 17617-1-AP | N/A |
| anti-CD20 | abcam | ab64088 | SP32 |
| anti-CD21 | abcam | ab227662 | SP186 |
| anti-CCL21 | abcam | ab231116 | N/A |
| HRP-conjugated Affinipure Goat Anti-Rabbit IgG(H+L) | proteintech | SA00001-2 | N/A |
| HRP-conjugated Affinipure Goat Anti-Mouse IgG(H+L) | proteintech | SA00001-1 | N/A |
| FITC Goat Anti-Rat IgG (H+L) | ABclonal | AS019 | N/A |
| CoraLite594 – conjugated Goat Anti-Rabbit IgG(H+L) | proteintech | SA00013-4 | N/A |

**Table S7. Cell lines**

| Species | Category | Name | Supplier | Cat No. |
| --- | --- | --- | --- | --- |
| Homo sapiens | Normal hepatocyte line | LO-2 | Liver Cancer Institute, Zhongshan Hospital, Fudan University, Shanghai, China | N/A |
|  | HCC cell line | Hep3B | ATCC | HB-8064 |
|  |  | HepG2 | ATCC | HB-8065 |
|  |  | HCCLM3 | Cell Bank, Chinese Academy of Science, China | SCSP-5093 |
|  |  | MHCC97H | Cell Bank, Chinese Academy of Science, China | SCSP-5092 |
|  |  | MHCC97L | Liver Cancer Institute, Zhongshan Hospital, Fudan University, Shanghai, China | N/A |
|  |  | SMMC-7721 | Liver Cancer Institute, Zhongshan Hospital, Fudan University, Shanghai, China | N/A |
|  |  | Huh7 | Cell Bank, Chinese Academy of Science, China | SCSP-526 |
| Mus musculus | Mouse hepatoma cell line | Hepa1-6 | ATCC | CRL-1830 |
|  | Mouse lymphatic endothelial cell line | mLEC | HZBIO (Shanghai Huzhen Industrial Co., Ltd) | HZM-5023 |
|  | Mouse macrophage cell line | RAW264.7 | ATCC | TIB-71 |

**Table S8. CCR7-KO-related sequences**

|  | Sequence |
| --- | --- |
| sgRNA | 5’-GGCCACCTGGATGGTGATAA-3’ |
| PHY-Sg-00107-sgRNA | forward: 5’-CACCGGGCCACCTGGATGGTGATAA-3’  reverse: 5’-AAACTTATCACCATCCAGGTGGCC-3’ |
| Sequencing primer  U6- F | 5’-TGCAGGGGAAAGAATAGTAGAC-3’ |
| Sequencing result  CCR7-sgRNA-U6-Promoter | 5’-TGTTTTAAAATGGACTATCATATGCTTACCGTAACTTGAAAGTATTTCGATTTCTT  GGCTTTATATATCTTGTGGAAAGGACGAAACACCGGCCACCTGGATGGTGATAAGT  TTTAGAGCTAGAAATAGCAAGTTAAAATAAGGCTAGTCCGTTATCAACTTGAAAAA  GTGGCACCGAGTCGGTGCTTTTTTGAATTCGCTAGCTAGGT-3’ |

**Table S9. CCR7-OE-related sequences**

|  | Sequence |
| --- | --- |
| Full-length CCR7 | 5’-ATGGACCTGGGGAAACCAATGAAAAGCGTGCTGGTGGTGGCTCTCCTTGTCATTTTCCAGGTATGCCTGTGTCAAGATGAGGTCACGGACGATTACATCGGAGACAACACCACAGTGGACTACACTTTGTTCGAGTCTTTGTGCTCCAAGAAGGACGTGCGGAACTTTAAAGCCTGGTTCCTCCCTATCATGTACTCCATCATTTGTTTCGTGGGCCTACTGGGCAATGGGCTGGTCGTGTTGACCTATATCTATTTCAAGAGGCTCAAGACCATGACCGATACCTACCTGCTCAACCTGGCGGTGGCAGACATCCTCTTCCTCCTGACCCTTCCCTTCTGGGCCTACAGCGCGGCCAAGTCCTGGGTCTTCGGTGTCCACTTTTGCAAGCTCATCTTTGCCATCTACAAGATGAGCTTCTTCAGTGGCATGCTCCTACTTCTTTGCATCAGCATTGACCGCTACGTGGCCATCGTCCAGGCTGTCTCAGCTCACCGCCACCGTGCCCGCGTCCTTCTCATCAGCAAGCTGTCCTGTGTGGGCATCTGGATACTAGCCACAGTGCTCTCCATCCCAGAGCTCCTGTACAGTGACCTCCAGAGGAGCAGCAGTGAGCAAGCGATGCGATGCTCTCTCATCACAGAGCATGTGGAGGCCTTTATCACCATCCAGGTGGCCCAGATGGTGATCGGCTTTCTGGTCCCCCTGCTGGCCATGAGCTTCTGTTACCTTGTCATCATCCGCACCCTGCTCCAGGCACGCAACTTTGAGCGCAACAAGGCCATCAAGGTGATCATCGCTGTGGTCGTGGTCTTCATAGTCTTCCAGCTGCCCTACAATGGGGTGGTCCTGGCCCAGACGGTGGCCAACTTCAACATCACCAGTAGCACCTGTGAGCTCAGTAAGCAACTCAACATCGCCTACGACGTCACCTACAGCCTGGCCTGCGTCCGCTGCTGCGTCAACCCTTTCTTGTACGCCTTCATCGGCGTCAAGTTCCGCAACGATCTCTTCAAGCTCTTCAAGGACCTGGGCTGCCTCAGCCAGGAGCAGCTCCGGCAGTGGTCTTCCTGTCGGCACATCCGGCGCTCCTCCATGAGTGTGGAGGCCGAGACCACCACCACCTTCTCCCCATAG-3’ |
