# Supplementary material for: Differential expression pattern of CC chemokine receptor 7 guides precision treatment of hepatocellular carcinoma
Source: Signal Transduct Target Ther. 2025 Jul 21;10:229. doi: 10.1038/s41392-025-02308-6 (PMC12277428; doi:10.1038/s41392-025-02308-6)
Supplement: Supplementary file 2 — Supplementary TMA Data 1-for SIGTRANS-16557R1 [file 41392_2025_2308_MOESM2_ESM.docx]

Supplementary Materials for

**Differential expression pattern of CC chemokine receptor 7 guides precision treatment of hepatocellular carcinoma**

**Running title:** The Effect of CCL21/CCR7 axis in Tumor Microenvironment

Jie Qin^1†^, Qianyi Gong^1†^, Cheng Zhou^2†^, Jietian Xu^1,3†^, Yifei Cheng^2†^, Weiyue Xu^1^, Di Zhu^1^, Yiming Liu^1^, Yuye Zhang^1^, Yanru Wang^1^, Lingling Gao^1^, Lanfang Li^1^, Wulei Hou^1^, Qian Li^1^, Binbin Liu^2^, Yazhen Zhu^4^, Zuoyun Wang^1^, Jieyi Shi^2*^, Shuangjian Qiu^2*^, Chunmin Liang^1,2,3*^

* Correspondence to: cmliang@fudan.edu.cn (Chunmin Liang);

qiu.shuangjian@zs-hospital.sh.cn (Shuangjian Qiu);

shi.jieyi@zs-hospital.sh.cn (Jieyi Shi);

† These authors have contributed equally to this work and share first authorship

**This file includes:**  TMA Data S1

**TMA Data S1. 240 HCC patients from Zhongshan Hospital Cohort**

| No. | Age (years) | Gender | Tumor size (cm) | TNM stage | CCR7 expression on tumor cells | OS-Time (Months) | OS-Status |
| --- | --- | --- | --- | --- | --- | --- | --- |
| 1 | 47 | Male | > 5 | Ⅲ-Ⅳ | Low | 60 | Censored |
| 2 | 65 | Male | > 5 | Ⅲ-Ⅳ | High | 11 | Die |
| 3 | 38 | Male | <= 5 | Ⅲ-Ⅳ | High | 39 | Die |
| 4 | 49 | Male | <= 5 | Ⅲ-Ⅳ | Low | 11 | Censored |
| 5 | 71 | Male | > 5 | Ⅲ-Ⅳ | Low | 14 | Die |
| 6 | 70 | Male | > 5 | Ⅲ-Ⅳ | High | 13 | Censored |
| 7 | 55 | Male | > 5 | Ⅲ-Ⅳ | Low | 49 | Die |
| 8 | 58 | Male | > 5 | Ⅲ-Ⅳ | High | 60 | Censored |
| 9 | 62 | Male | <= 5 | Ⅰ-Ⅱ | Low | 60 | Censored |
| 10 | 50 | Female | > 5 | Ⅰ-Ⅱ | High | 11 | Die |
| 11 | 56 | Male | > 5 | Ⅲ-Ⅳ | High | 4 | Die |
| 12 | 56 | Male | > 5 | Ⅰ-Ⅱ | Low | 60 | Censored |
| 13 | 39 | Male | <= 5 | Ⅰ-Ⅱ | High | 60 | Censored |
| 14 | 57 | Male | <= 5 | Ⅲ-Ⅳ | High | 43 | Die |
| 15 | 56 | Male | <= 5 | Ⅰ-Ⅱ | Low | 32 | Die |
| 16 | 51 | Male | > 5 | Ⅰ-Ⅱ | Low | 29 | Censored |
| 17 | 50 | Male | > 5 | Ⅰ-Ⅱ | High | 60 | Censored |
| 18 | 57 | Female | <= 5 | Ⅰ-Ⅱ | High | 2 | Die |
| 19 | 52 | Male | <= 5 | Ⅰ-Ⅱ | High | 60 | Censored |
| 20 | 57 | Male | <= 5 | Ⅰ-Ⅱ | Low | 29 | Die |
| 21 | 52 | Male | > 5 | Ⅲ-Ⅳ | High | 35 | Die |
| 22 | 43 | Male | <= 5 | Ⅰ-Ⅱ | Low | 60 | Censored |
| 23 | 48 | Female | > 5 | Ⅲ-Ⅳ | High | 25 | Die |
| 24 | 46 | Male | > 5 | Ⅲ-Ⅳ | High | 16 | Die |
| 25 | 52 | Male | > 5 | Ⅲ-Ⅳ | Low | 60 | Censored |
| 26 | 52 | Male | > 5 | Ⅰ-Ⅱ | High | 16 | Die |
| 27 | 48 | Male | > 5 | Ⅲ-Ⅳ | High | 8 | Die |
| 28 | 56 | Male | > 5 | Ⅲ-Ⅳ | High | 6 | Die |
| 29 | 55 | Male | <= 5 | Ⅰ-Ⅱ | High | 48 | Censored |
| 30 | 54 | Male | <= 5 | Ⅰ-Ⅱ | Low | 17 | Die |
| 31 | 57 | Male | > 5 | Ⅲ-Ⅳ | High | 11 | Die |
| 32 | 43 | Male | > 5 | Ⅲ-Ⅳ | High | 4 | Die |
| 33 | 61 | Male | <= 5 | Ⅰ-Ⅱ | High | 60 | Censored |
| 34 | 68 | Male | > 5 | Ⅲ-Ⅳ | High | 1 | Die |
| 35 | 42 | Male | <= 5 | Ⅰ-Ⅱ | High | 3 | Die |
| 36 | 54 | Male | <= 5 | Ⅰ-Ⅱ | Low | 60 | Censored |
| 37 | 48 | Male | > 5 | Ⅰ-Ⅱ | Low | 60 | Censored |
| 38 | 51 | Male | > 5 | Ⅲ-Ⅳ | High | 17 | Die |
| 39 | 37 | Male | > 5 | Ⅲ-Ⅳ | High | 6 | Die |
| 40 | 52 | Male | > 5 | Ⅲ-Ⅳ | High | 30 | Die |
| 41 | 57 | Male | > 5 | Ⅲ-Ⅳ | High | 32 | Censored |
| 42 | 52 | Male | > 5 | Ⅲ-Ⅳ | High | 55 | Die |
| 43 | 54 | Male | <= 5 | Ⅰ-Ⅱ | High | 54 | Die |
| 44 | 51 | Male | <= 5 | Ⅰ-Ⅱ | Low | 5 | Die |
| 45 | 54 | Female | <= 5 | Ⅰ-Ⅱ | Low | 16 | Censored |
| 46 | 65 | Male | > 5 | Ⅲ-Ⅳ | High | 4 | Die |
| 47 | 67 | Female | <= 5 | Ⅰ-Ⅱ | High | 54 | Die |
| 48 | 40 | Male | > 5 | Ⅲ-Ⅳ | High | 50 | Die |
| 49 | 59 | Male | > 5 | Ⅲ-Ⅳ | High | 50 | Censored |
| 50 | 40 | Male | > 5 | Ⅲ-Ⅳ | High | 5 | Die |
| 51 | 63 | Male | > 5 | Ⅰ-Ⅱ | Low | 60 | Censored |
| 52 | 56 | Male | <= 5 | Ⅰ-Ⅱ | High | 5 | Die |
| 53 | 57 | Male | > 5 | Ⅲ-Ⅳ | Low | 19 | Die |
| 54 | 70 | Female | <= 5 | Ⅲ-Ⅳ | High | 46 | Censored |
| 55 | 38 | Male | > 5 | Ⅲ-Ⅳ | High | 16 | Die |
| 56 | 65 | Male | > 5 | Ⅲ-Ⅳ | High | 35 | Die |
| 57 | 47 | Male | <= 5 | Ⅰ-Ⅱ | Low | 60 | Censored |
| 58 | 56 | Male | <= 5 | Ⅰ-Ⅱ | Low | 60 | Censored |
| 59 | 50 | Male | > 5 | Ⅲ-Ⅳ | High | 2 | Die |
| 60 | 56 | Male | > 5 | Ⅲ-Ⅳ | High | 37 | Die |
| 61 | 71 | Female | <= 5 | Ⅰ-Ⅱ | Low | 60 | Censored |
| 62 | 46 | Male | > 5 | Ⅰ-Ⅱ | High | 19 | Die |
| 63 | 55 | Male | > 5 | Ⅰ-Ⅱ | Low | 60 | Censored |
| 64 | 73 | Male | > 5 | Ⅲ-Ⅳ | Low | 32 | Die |
| 65 | 62 | Male | <= 5 | Ⅰ-Ⅱ | Low | 60 | Censored |
| 66 | 53 | Male | > 5 | Ⅰ-Ⅱ | High | 38 | Die |
| 67 | 63 | Male | <= 5 | Ⅰ-Ⅱ | Low | 12 | Die |
| 68 | 72 | Male | <= 5 | Ⅰ-Ⅱ | High | 37 | Censored |
| 69 | 47 | Female | > 5 | Ⅰ-Ⅱ | High | 37 | Die |
| 70 | 53 | Male | > 5 | Ⅲ-Ⅳ | High | 20 | Die |
| 71 | 47 | Male | > 5 | Ⅲ-Ⅳ | Low | 60 | Censored |
| 72 | 65 | Male | > 5 | Ⅲ-Ⅳ | High | 12 | Censored |
| 73 | 49 | Male | <= 5 | Ⅲ-Ⅳ | Low | 11 | Die |
| 74 | 71 | Male | > 5 | Ⅲ-Ⅳ | Low | 14 | Censored |
| 75 | 55 | Male | > 5 | Ⅲ-Ⅳ | Low | 49 | Die |
| 76 | 58 | Male | > 5 | Ⅲ-Ⅳ | High | 60 | Die |
| 77 | 62 | Male | <= 5 | Ⅰ-Ⅱ | Low | 60 | Censored |
| 78 | 50 | Female | > 5 | Ⅰ-Ⅱ | High | 11 | Censored |
| 79 | 56 | Male | > 5 | Ⅲ-Ⅳ | High | 4 | Die |
| 80 | 56 | Male | > 5 | Ⅰ-Ⅱ | Low | 60 | Die |
| 81 | 39 | Male | <= 5 | Ⅰ-Ⅱ | High | 11 | Censored |
| 82 | 56 | Male | <= 5 | Ⅰ-Ⅱ | Low | 32 | Die |
| 83 | 51 | Male | > 5 | Ⅰ-Ⅱ | Low | 29 | Censored |
| 84 | 50 | Male | > 5 | Ⅰ-Ⅱ | High | 60 | Censored |
| 85 | 57 | Female | <= 5 | Ⅰ-Ⅱ | High | 2 | Die |
| 86 | 52 | Male | <= 5 | Ⅰ-Ⅱ | High | 58 | Die |
| 87 | 57 | Male | <= 5 | Ⅰ-Ⅱ | Low | 29 | Censored |
| 88 | 52 | Male | > 5 | Ⅲ-Ⅳ | High | 35 | Die |
| 89 | 43 | Male | <= 5 | Ⅰ-Ⅱ | Low | 60 | Censored |
| 90 | 48 | Female | > 5 | Ⅲ-Ⅳ | High | 25 | Die |
| 91 | 46 | Male | > 5 | Ⅲ-Ⅳ | High | 16 | Censored |
| 92 | 52 | Male | > 5 | Ⅲ-Ⅳ | Low | 60 | Censored |
| 93 | 56 | Male | > 5 | Ⅲ-Ⅳ | High | 6 | Die |
| 94 | 55 | Male | <= 5 | Ⅰ-Ⅱ | High | 15 | Censored |
| 95 | 54 | Male | <= 5 | Ⅰ-Ⅱ | Low | 17 | Die |
| 96 | 57 | Male | > 5 | Ⅲ-Ⅳ | High | 11 | Die |
| 97 | 43 | Male | > 5 | Ⅲ-Ⅳ | High | 4 | Die |
| 98 | 61 | Male | <= 5 | Ⅰ-Ⅱ | High | 60 | Censored |
| 99 | 68 | Male | > 5 | Ⅲ-Ⅳ | High | 1 | Die |
| 100 | 54 | Male | <= 5 | Ⅰ-Ⅱ | Low | 55 | Die |
| 101 | 48 | Male | > 5 | Ⅰ-Ⅱ | Low | 60 | Censored |
| 102 | 51 | Male | > 5 | Ⅲ-Ⅳ | High | 17 | Die |
| 103 | 37 | Male | > 5 | Ⅲ-Ⅳ | High | 6 | Die |
| 104 | 54 | Male | <= 5 | Ⅰ-Ⅱ | High | 54 | Die |
| 105 | 51 | Male | <= 5 | Ⅰ-Ⅱ | Low | 5 | Die |
| 106 | 54 | Female | <= 5 | Ⅰ-Ⅱ | Low | 16 | Censored |
| 107 | 65 | Male | > 5 | Ⅲ-Ⅳ | High | 4 | Die |
| 108 | 40 | Male | > 5 | Ⅲ-Ⅳ | High | 5 | Die |
| 109 | 63 | Male | > 5 | Ⅰ-Ⅱ | Low | 60 | Censored |
| 110 | 56 | Male | <= 5 | Ⅰ-Ⅱ | High | 5 | Die |
| 111 | 57 | Male | > 5 | Ⅲ-Ⅳ | Low | 19 | Die |
| 112 | 70 | Female | <= 5 | Ⅲ-Ⅳ | High | 46 | Censored |
| 113 | 38 | Male | > 5 | Ⅲ-Ⅳ | High | 16 | Die |
| 114 | 65 | Male | > 5 | Ⅲ-Ⅳ | High | 35 | Censored |
| 115 | 47 | Male | <= 5 | Ⅰ-Ⅱ | Low | 57 | Censored |
| 116 | 56 | Male | <= 5 | Ⅰ-Ⅱ | Low | 50 | Censored |
| 117 | 50 | Male | > 5 | Ⅲ-Ⅳ | High | 2 | Die |
| 118 | 56 | Male | > 5 | Ⅲ-Ⅳ | High | 37 | Censored |
| 119 | 71 | Female | <= 5 | Ⅰ-Ⅱ | Low | 60 | Censored |
| 120 | 46 | Male | > 5 | Ⅰ-Ⅱ | High | 18 | Die |
| 121 | 55 | Male | > 5 | Ⅰ-Ⅱ | Low | 45 | Censored |
| 122 | 73 | Male | > 5 | Ⅲ-Ⅳ | Low | 28 | Die |
| 123 | 62 | Male | <= 5 | Ⅰ-Ⅱ | Low | 50 | Censored |
| 124 | 53 | Male | > 5 | Ⅰ-Ⅱ | High | 32 | Die |
| 125 | 63 | Male | <= 5 | Ⅰ-Ⅱ | Low | 15 | Die |
| 126 | 72 | Male | <= 5 | Ⅰ-Ⅱ | High | 30 | Censored |
| 127 | 47 | Female | > 5 | Ⅰ-Ⅱ | High | 34 | Die |
| 128 | 53 | Male | > 5 | Ⅲ-Ⅳ | High | 24 | Die |
| 129 | 47 | Male | > 5 | Ⅲ-Ⅳ | Low | 60 | Censored |
| 130 | 65 | Male | > 5 | Ⅲ-Ⅳ | High | 10 | Die |
| 131 | 38 | Male | <= 5 | Ⅲ-Ⅳ | High | 33 | Censored |
| 132 | 49 | Male | <= 5 | Ⅲ-Ⅳ | Low | 18 | Die |
| 133 | 71 | Male | > 5 | Ⅲ-Ⅳ | Low | 15 | Die |
| 134 | 70 | Male | > 5 | Ⅲ-Ⅳ | High | 17 | Die |
| 135 | 55 | Male | > 5 | Ⅲ-Ⅳ | Low | 44 | Censored |
| 136 | 58 | Male | > 5 | Ⅲ-Ⅳ | High | 43 | Censored |
| 137 | 62 | Male | <= 5 | Ⅰ-Ⅱ | Low | 57 | Censored |
| 138 | 50 | Female | > 5 | Ⅰ-Ⅱ | High | 13 | Die |
| 139 | 56 | Male | > 5 | Ⅲ-Ⅳ | High | 6 | Die |
| 140 | 56 | Male | > 5 | Ⅰ-Ⅱ | Low | 55 | Censored |
| 141 | 39 | Male | <= 5 | Ⅰ-Ⅱ | High | 58 | Censored |
| 142 | 57 | Male | <= 5 | Ⅲ-Ⅳ | High | 41 | Die |
| 143 | 56 | Male | <= 5 | Ⅰ-Ⅱ | Low | 35 | Censored |
| 144 | 51 | Male | > 5 | Ⅰ-Ⅱ | Low | 32 | Die |
| 145 | 50 | Male | > 5 | Ⅰ-Ⅱ | High | 51 | Censored |
| 146 | 57 | Female | <= 5 | Ⅰ-Ⅱ | High | 2 | Die |
| 147 | 52 | Male | <= 5 | Ⅰ-Ⅱ | High | 52 | Censored |
| 148 | 57 | Male | <= 5 | Ⅰ-Ⅱ | Low | 22 | Die |
| 149 | 52 | Male | > 5 | Ⅲ-Ⅳ | High | 33 | Die |
| 150 | 70 | Female | <= 5 | Ⅲ-Ⅳ | High | 44 | Die |
| 151 | 38 | Male | > 5 | Ⅲ-Ⅳ | High | 18 | Die |
| 152 | 65 | Male | > 5 | Ⅲ-Ⅳ | High | 33 | Die |
| 153 | 50 | Male | > 5 | Ⅲ-Ⅳ | High | 4 | Die |
| 154 | 56 | Male | > 5 | Ⅲ-Ⅳ | High | 37 | Die |
| 155 | 71 | Female | <= 5 | Ⅰ-Ⅱ | Low | 50 | Censored |
| 156 | 46 | Male | > 5 | Ⅰ-Ⅱ | High | 22 | Die |
| 157 | 55 | Male | > 5 | Ⅰ-Ⅱ | Low | 47 | Censored |
| 158 | 73 | Male | > 5 | Ⅲ-Ⅳ | Low | 32 | Die |
| 159 | 62 | Male | <= 5 | Ⅰ-Ⅱ | Low | 45 | Censored |
| 160 | 53 | Male | > 5 | Ⅰ-Ⅱ | High | 38 | Die |
| 161 | 63 | Male | <= 5 | Ⅰ-Ⅱ | Low | 11 | Die |
| 162 | 72 | Male | <= 5 | Ⅰ-Ⅱ | High | 36 | Die |
| 163 | 47 | Female | > 5 | Ⅰ-Ⅱ | High | 33 | Die |
| 164 | 53 | Male | > 5 | Ⅲ-Ⅳ | High | 26 | Die |
| 165 | 47 | Male | > 5 | Ⅲ-Ⅳ | Low | 56 | Censored |
| 166 | 65 | Male | > 5 | Ⅲ-Ⅳ | High | 11 | Die |
| 167 | 38 | Male | <= 5 | Ⅲ-Ⅳ | High | 37 | Die |
| 168 | 49 | Male | <= 5 | Ⅲ-Ⅳ | Low | 11 | Die |
| 169 | 71 | Male | > 5 | Ⅲ-Ⅳ | Low | 16 | Die |
| 170 | 70 | Male | > 5 | Ⅲ-Ⅳ | High | 16 | Die |
| 171 | 55 | Male | > 5 | Ⅲ-Ⅳ | Low | 44 | Censored |
| 172 | 58 | Male | > 5 | Ⅲ-Ⅳ | High | 52 | Censored |
| 173 | 62 | Male | <= 5 | Ⅰ-Ⅱ | Low | 60 | Censored |
| 174 | 50 | Female | > 5 | Ⅰ-Ⅱ | High | 13 | Die |
| 175 | 56 | Male | > 5 | Ⅲ-Ⅳ | High | 8 | Die |
| 176 | 56 | Male | > 5 | Ⅰ-Ⅱ | Low | 57 | Censored |
| 177 | 39 | Male | <= 5 | Ⅰ-Ⅱ | High | 55 | Censored |
| 178 | 57 | Male | <= 5 | Ⅲ-Ⅳ | High | 43 | Die |
| 179 | 56 | Male | <= 5 | Ⅰ-Ⅱ | Low | 34 | Censored |
| 180 | 51 | Male | > 5 | Ⅰ-Ⅱ | Low | 29 | Die |
| 181 | 50 | Male | > 5 | Ⅰ-Ⅱ | High | 48 | Censored |
| 182 | 57 | Female | <= 5 | Ⅰ-Ⅱ | High | 2 | Die |
| 183 | 52 | Male | <= 5 | Ⅰ-Ⅱ | High | 58 | Censored |
| 184 | 57 | Male | <= 5 | Ⅰ-Ⅱ | Low | 30 | Die |
| 185 | 52 | Male | > 5 | Ⅲ-Ⅳ | High | 33 | Die |
| 186 | 43 | Male | <= 5 | Ⅰ-Ⅱ | Low | 47 | Censored |
| 187 | 46 | Male | > 5 | Ⅲ-Ⅳ | High | 16 | Censored |
| 188 | 52 | Male | > 5 | Ⅲ-Ⅳ | Low | 53 | Censored |
| 189 | 52 | Male | > 5 | Ⅰ-Ⅱ | High | 16 | Die |
| 190 | 48 | Male | > 5 | Ⅲ-Ⅳ | High | 8 | Censored |
| 191 | 56 | Male | > 5 | Ⅲ-Ⅳ | High | 6 | Die |
| 192 | 55 | Male | <= 5 | Ⅰ-Ⅱ | High | 33 | Die |
| 193 | 54 | Male | <= 5 | Ⅰ-Ⅱ | Low | 22 | Censored |
| 194 | 52 | Male | > 5 | Ⅲ-Ⅳ | High | 46 | Die |
| 195 | 54 | Male | <= 5 | Ⅰ-Ⅱ | High | 42 | Censored |
| 196 | 51 | Male | <= 5 | Ⅰ-Ⅱ | Low | 5 | Die |
| 197 | 54 | Female | <= 5 | Ⅰ-Ⅱ | Low | 16 | Censored |
| 198 | 65 | Male | > 5 | Ⅲ-Ⅳ | High | 4 | Die |
| 199 | 67 | Female | <= 5 | Ⅰ-Ⅱ | High | 54 | Die |
| 200 | 40 | Male | > 5 | Ⅲ-Ⅳ | High | 56 | Censored |
| 201 | 59 | Male | > 5 | Ⅲ-Ⅳ | High | 47 | Censored |
| 202 | 40 | Male | > 5 | Ⅲ-Ⅳ | High | 5 | Die |
| 203 | 63 | Male | > 5 | Ⅰ-Ⅱ | Low | 60 | Censored |
| 204 | 56 | Male | <= 5 | Ⅰ-Ⅱ | High | 5 | Die |
| 205 | 57 | Male | > 5 | Ⅲ-Ⅳ | Low | 22 | Censored |
| 206 | 70 | Female | <= 5 | Ⅲ-Ⅳ | High | 40 | Censored |
| 207 | 38 | Male | > 5 | Ⅲ-Ⅳ | High | 10 | Censored |
| 208 | 47 | Male | <= 5 | Ⅰ-Ⅱ | Low | 60 | Censored |
| 209 | 56 | Male | <= 5 | Ⅰ-Ⅱ | Low | 43 | Censored |
| 210 | 50 | Male | > 5 | Ⅲ-Ⅳ | High | 2 | Die |
| 211 | 56 | Male | > 5 | Ⅲ-Ⅳ | High | 31 | Censored |
| 212 | 71 | Female | <= 5 | Ⅰ-Ⅱ | Low | 60 | Censored |
| 213 | 46 | Male | > 5 | Ⅰ-Ⅱ | High | 19 | Die |
| 214 | 55 | Male | > 5 | Ⅰ-Ⅱ | Low | 44 | Censored |
| 215 | 73 | Male | > 5 | Ⅲ-Ⅳ | Low | 35 | Censored |
| 216 | 62 | Male | <= 5 | Ⅰ-Ⅱ | Low | 60 | Censored |
| 217 | 63 | Male | <= 5 | Ⅰ-Ⅱ | Low | 12 | Die |
| 218 | 47 | Female | > 5 | Ⅰ-Ⅱ | High | 38 | Censored |
| 219 | 53 | Male | > 5 | Ⅲ-Ⅳ | High | 20 | Die |
| 220 | 47 | Male | > 5 | Ⅲ-Ⅳ | Low | 60 | Censored |
| 221 | 65 | Male | > 5 | Ⅲ-Ⅳ | High | 11 | Die |
| 222 | 38 | Male | <= 5 | Ⅲ-Ⅳ | High | 39 | Die |
| 223 | 49 | Male | <= 5 | Ⅲ-Ⅳ | Low | 11 | Die |
| 224 | 71 | Male | > 5 | Ⅲ-Ⅳ | Low | 18 | Censored |
| 225 | 70 | Male | > 5 | Ⅲ-Ⅳ | High | 13 | Die |
| 226 | 55 | Male | > 5 | Ⅲ-Ⅳ | Low | 49 | Die |
| 227 | 62 | Male | <= 5 | Ⅰ-Ⅱ | Low | 60 | Censored |
| 228 | 50 | Female | > 5 | Ⅰ-Ⅱ | High | 11 | Die |
| 229 | 56 | Male | > 5 | Ⅲ-Ⅳ | High | 4 | Die |
| 230 | 56 | Male | > 5 | Ⅰ-Ⅱ | Low | 60 | Censored |
| 231 | 39 | Male | <= 5 | Ⅰ-Ⅱ | High | 58 | Die |
| 232 | 57 | Male | <= 5 | Ⅲ-Ⅳ | High | 43 | Censored |
| 233 | 56 | Male | <= 5 | Ⅰ-Ⅱ | Low | 32 | Die |
| 234 | 51 | Male | > 5 | Ⅰ-Ⅱ | Low | 33 | Censored |
| 235 | 57 | Male | <= 5 | Ⅰ-Ⅱ | Low | 29 | Die |
| 236 | 43 | Male | <= 5 | Ⅰ-Ⅱ | Low | 60 | Censored |
| 237 | 46 | Male | > 5 | Ⅲ-Ⅳ | High | 16 | Die |
| 238 | 56 | Male | <= 5 | Ⅰ-Ⅱ | Low | 53 | Censored |
| 239 | 46 | Male | > 5 | Ⅰ-Ⅱ | High | 19 | Die |
| 240 | 50 | Female | > 5 | Ⅰ-Ⅱ | High | 11 | Die |
