# Supplementary material for: Differential expression pattern of CC chemokine receptor 7 guides precision treatment of hepatocellular carcinoma
Source: Signal Transduct Target Ther. 2025 Jul 21;10:229. doi: 10.1038/s41392-025-02308-6 (PMC12277428; doi:10.1038/s41392-025-02308-6)
Supplement: Supplementary file 3 — Supplementary TMA Data 2-for SIGTRANS-16557R1 [file 41392_2025_2308_MOESM3_ESM.docx]

Supplementary Materials for

**Differential expression pattern of CC chemokine receptor 7 guides precision treatment of hepatocellular carcinoma**

**Running title:** The Effect of CCL21/CCR7 axis in Tumor Microenvironment

Jie Qin^1†^, Qianyi Gong^1†^, Cheng Zhou^2†^, Jietian Xu^1,3†^, Yifei Cheng^2†^, Weiyue Xu^1^, Di Zhu^1^, Yiming Liu^1^, Yuye Zhang^1^, Yanru Wang^1^, Lingling Gao^1^, Lanfang Li^1^, Wulei Hou^1^, Qian Li^1^, Binbin Liu^2^, Yazhen Zhu^4^, Zuoyun Wang^1^, Jieyi Shi^2*^, Shuangjian Qiu^2*^, Chunmin Liang^1,2,3*^

* Correspondence to: cmliang@fudan.edu.cn (Chunmin Liang);

qiu.shuangjian@zs-hospital.sh.cn (Shuangjian Qiu);

shi.jieyi@zs-hospital.sh.cn (Jieyi Shi);

† These authors have contributed equally to this work and share first authorship

**This file includes:** TMA Data S2

**TMA Data S2. 382 HCC patients from Zhongshan Hospital Cohort**

| Number | Age (years) | Gender | Cirrhosis | Tumor size (cm) | Tumor number | BCLC stage | TNM stage | Recurrence | Expression of VEGF-C in peritumor | | | Expression of VEGF-C in tumor | | | Expression of CCR7 in mesenchymal sites | OS.Time (Months) | OS. Status |
| --- | --- | --- | --- | --- | --- | --- | --- | --- | --- | --- | --- | --- | --- | --- | --- | --- | --- |
| 1 | 36 | Male | Yes | > 5 | Multiple | B-C | Ⅲ-Ⅳ | No | 0 | Negative | Low | 0 | Negative | Low | Low | 9.0 | Die |
| 2 | 44 | Male | Yes | <= 5 | Single | B-C | Ⅲ-Ⅳ | No | 0 | Negative | Low | 0 | Negative | Low | Low | 109.0 | Censored |
| 3 | 62 | Male | No | > 5 | Single | B-C | Ⅲ-Ⅳ | No | 0 | Negative | Low | 0 | Negative | Low | Low | 109.0 | Censored |
| 4 | 43 | Male | Yes | > 5 | Single | B-C | Ⅲ-Ⅳ | No | 0 | Negative | Low | 0 | Negative | Low | High | 39.0 | Censored |
| 5 | 47 | Male | Yes | > 5 | Multiple | B-C | Ⅲ-Ⅳ | No | 0 | Negative | Low | 1 | Weak | Low | Low | 15.0 | Censored |
| 6 | 59 | Female | Yes | > 5 | Single | B-C | Ⅰ-Ⅱ | Yes | 0 | Negative | Low | 0 | Negative | Low | Low | 27.0 | Die |
| 7 | 36 | Male | Yes | <= 5 | Single | B-C | Ⅲ-Ⅳ | No | 1 | Weak | Low | 0 | Negative | Low | Low | 109.0 | Censored |
| 8 | 57 | Male | No | > 5 | Multiple | B-C | Ⅲ-Ⅳ | No | 1 | Weak | Low | 1 | Weak | Low | Low | 99.0 | Die |
| 9 | 56 | Male | Yes | > 5 | Single | B-C | Ⅰ-Ⅱ | Yes | 1 | Weak | Low | 0 | Negative | Low | Low | 30.0 | Die |
| 10 | 59 | Male | Yes | > 5 | Multiple | B-C | Ⅲ-Ⅳ | Yes | 1 | Weak | Low | 0 | Negative | Low | High | 22.0 | Die |
| 11 | 51 | Male | Yes | <= 5 | Single | 0-A | Ⅰ-Ⅱ | No | 1 | Weak | Low | 0 | Negative | Low | Low | 109.0 | Censored |
| 12 | 72 | Male | No | <= 5 | Single | 0-A | Ⅰ-Ⅱ | No | 2 | Moderate | High | 2 | Moderate | High | Low | 2.0 | Die |
| 13 | 63 | Male | Yes | <= 5 | Single | 0-A | Ⅰ-Ⅱ | No | 1 | Weak | Low | 0 | Negative | Low | Low | 109.0 | Censored |
| 14 | 57 | Female | Yes | <= 5 | Single | 0-A | Ⅰ-Ⅱ | No | 1 | Weak | Low | 0 | Negative | Low | High | 109.0 | Censored |
| 15 | 57 | Male | No | > 5 | Multiple | B-C | Ⅲ-Ⅳ | No | 1 | Weak | Low | 0 | Negative | Low | Low | 11.0 | Die |
| 16 | 62 | Male | Yes | <= 5 | Single | 0-A | Ⅰ-Ⅱ | Yes | 2 | Moderate | High | 1 | Weak | Low | Low | 109.0 | Censored |
| 17 | 51 | Male | Yes | > 5 | Single | B-C | Ⅲ-Ⅳ | Yes | 2 | Moderate | High | 0 | Negative | Low | High | 25.0 | Die |
| 18 | 37 | Male | No | <= 5 | Single | B-C | Ⅲ-Ⅳ | No | 1 | Weak | Low | 0 | Negative | Low | High | 109.0 | Censored |
| 19 | 56 | Male | Yes | > 5 | Multiple | B-C | Ⅲ-Ⅳ | Yes | 3 | Strong | High | 1 | Weak | Low | High | 41.0 | Die |
| 20 | 55 | Female | No | <= 5 | Single | 0-A | Ⅰ-Ⅱ | No | 1 | Weak | Low | 0 | Negative | Low | Low | 109.0 | Censored |
| 21 | 58 | Male | Yes | <= 5 | Single | 0-A | Ⅰ-Ⅱ | No | 2 | Moderate | High | 2 | Moderate | High | High | 85.0 | Censored |
| 22 | 41 | Male | No | <= 5 | Single | 0-A | Ⅰ-Ⅱ | Yes | 2 | Moderate | High | 0 | Negative | Low | Low | 9.0 | Die |
| 23 | 53 | Male | Yes | > 5 | Multiple | B-C | Ⅲ-Ⅳ | Yes | 1 | Weak | Low | 1 | Weak | Low | Low | 31.0 | Die |
| 24 | 36 | Male | Yes | <= 5 | Single | 0-A | Ⅰ-Ⅱ | No | 0 | Negative | Low | 0 | Negative | Low | Low | 108.0 | Censored |
| 25 | 52 | Male | Yes | <= 5 | Multiple | B-C | Ⅲ-Ⅳ | Yes | 1 | Weak | Low | 1 | Weak | Low | Low | 32.0 | Die |
| 26 | 61 | Male | Yes | <= 5 | Single | B-C | Ⅲ-Ⅳ | Yes | 1 | Weak | Low | 0 | Negative | Low | Low | 45.0 | Die |
| 27 | 42 | Male | Yes | <= 5 | Single | B-C | Ⅲ-Ⅳ | No | 1 | Weak | Low | 0 | Negative | Low | High | 108.0 | Censored |
| 28 | 55 | Male | Yes | > 5 | Single | B-C | Ⅲ-Ⅳ | Yes | 0 | Negative | Low | 0 | Negative | Low | Low | 84.0 | Die |
| 29 | 53 | Male | No | <= 5 | Multiple | 0-A | Ⅲ-Ⅳ | No | 2 | Moderate | High | 0 | Negative | Low | High | 38.0 | Die |
| 30 | 68 | Male | No | > 5 | Multiple | B-C | Ⅲ-Ⅳ | No | 3 | Strong | High | 0 | Negative | Low | High | 93.0 | Die |
| 31 | 52 | Male | Yes | > 5 | Single | B-C | Ⅰ-Ⅱ | No | 0 | Negative | Low | 0 | Negative | Low | Low | 108.0 | Censored |
| 32 | 42 | Male | Yes | > 5 | Single | B-C | Ⅰ-Ⅱ | No | 3 | Strong | High | 0 | Negative | Low | Low | 108.0 | Censored |
| 33 | 30 | Male | No | > 5 | Single | B-C | Ⅰ-Ⅱ | No | 3 | Strong | High | 0 | Negative | Low | Low | 15.0 | Die |
| 34 | 48 | Male | No | <= 5 | Single | 0-A | Ⅰ-Ⅱ | No | 3 | Strong | High | 1 | Weak | Low | High | 108.0 | Censored |
| 35 | 44 | Male | Yes | <= 5 | Single | 0-A | Ⅰ-Ⅱ | Yes | 1 | Weak | Low | 0 | Negative | Low | Low | 33.0 | Die |
| 36 | 59 | Male | Yes | > 5 | Single | B-C | Ⅲ-Ⅳ | No | 1 | Weak | Low | 0 | Negative | Low | Low | 8.0 | Die |
| 37 | 65 | Female | Yes | <= 5 | Single | B-C | Ⅲ-Ⅳ | Yes | 1 | Weak | Low | 0 | Negative | Low | High | 11.0 | Die |
| 38 | 57 | Male | Yes | > 5 | Multiple | B-C | Ⅲ-Ⅳ | No | 0 | Negative | Low | 0 | Negative | Low | Low | 108.0 | Censored |
| 39 | 41 | Male | Yes | > 5 | Single | B-C | Ⅰ-Ⅱ | No | 1 | Weak | Low | 0 | Negative | Low | High | 20.0 | Die |
| 40 | 43 | Male | Yes | > 5 | Single | B-C | Ⅲ-Ⅳ | Yes | 3 | Strong | High | 2 | Moderate | High | Low | 7.0 | Die |
| 41 | 75 | Male | Yes | > 5 | Single | B-C | Ⅰ-Ⅱ | No | 1 | Weak | Low | 0 | Negative | Low | Low | 89.0 | Die |
| 42 | 53 | Male | Yes | <= 5 | Single | 0-A | Ⅰ-Ⅱ | No | 3 | Strong | High | 0 | Negative | Low | High | 108.0 | Censored |
| 43 | 57 | Male | Yes | <= 5 | Single | 0-A | Ⅰ-Ⅱ | No | 3 | Strong | High | 1 | Weak | Low | High | 108.0 | Censored |
| 44 | 68 | Male | No | <= 5 | Single | B-C | Ⅲ-Ⅳ | No | 3 | Strong | High | 1 | Weak | Low | Low | 108.0 | Censored |
| 45 | 58 | Male | Yes | > 5 | Single | B-C | Ⅰ-Ⅱ | Yes | 3 | Strong | High | 0 | Negative | Low | High | 65.0 | Die |
| 46 | 55 | Female | Yes | <= 5 | Single | 0-A | Ⅰ-Ⅱ | No | 0 | Negative | Low | 0 | Negative | Low | Low | 108.0 | Censored |
| 47 | 73 | Female | Yes | <= 5 | Single | 0-A | Ⅰ-Ⅱ | No | 2 | Moderate | High | 1 | Weak | Low | Low | 52.0 | Die |
| 48 | 60 | Male | Yes | <= 5 | Single | 0-A | Ⅰ-Ⅱ | Yes | 1 | Weak | Low | 0 | Negative | Low | Low | 25.0 | Die |
| 49 | 37 | Female | Yes | <= 5 | Single | 0-A | Ⅰ-Ⅱ | No | 3 | Strong | High | 0 | Negative | Low | Low | 108.0 | Censored |
| 50 | 49 | Female | Yes | <= 5 | Single | B-C | Ⅲ-Ⅳ | No | 3 | Strong | High | 2 | Moderate | High | Low | 108.0 | Censored |
| 51 | 55 | Male | Yes | > 5 | Single | B-C | Ⅰ-Ⅱ | No | 2 | Moderate | High | 1 | Weak | Low | Low | 66.0 | Censored |
| 52 | 39 | Male | Yes | > 5 | Single | B-C | Ⅰ-Ⅱ | No | 3 | Strong | High | 0 | Negative | Low | High | 84.0 | Censored |
| 53 | 75 | Female | Yes | > 5 | Multiple | B-C | Ⅲ-Ⅳ | No | 2 | Moderate | High | 1 | Weak | Low | Low | 107.0 | Censored |
| 54 | 29 | Female | Yes | > 5 | Single | B-C | Ⅰ-Ⅱ | No | 3 | Strong | High | 1 | Weak | Low | Low | 107.0 | Censored |
| 55 | 40 | Male | Yes | <= 5 | Single | B-C | Ⅲ-Ⅳ | No | 1 | Weak | Low | 1 | Weak | Low | Low | 107.0 | Censored |
| 56 | 41 | Male | Yes | <= 5 | Single | 0-A | Ⅰ-Ⅱ | No | 1 | Weak | Low | 0 | Negative | Low | Low | 107.0 | Censored |
| 57 | 71 | Female | No | <= 5 | Single | 0-A | Ⅰ-Ⅱ | Yes | 1 | Weak | Low | 1 | Weak | Low | Low | 59.0 | Censored |
| 58 | 50 | Male | Yes | > 5 | Single | B-C | Ⅲ-Ⅳ | No | 1 | Weak | Low | 0 | Negative | Low | Low | 107.0 | Censored |
| 59 | 41 | Male | Yes | > 5 | Multiple | B-C | Ⅲ-Ⅳ | No | 1 | Weak | Low | 0 | Negative | Low | Low | 107.0 | Censored |
| 60 | 62 | Female | No | <= 5 | Single | 0-A | Ⅰ-Ⅱ | No | 2 | Moderate | High | 1 | Weak | Low | Low | 107.0 | Censored |
| 61 | 55 | Male | Yes | <= 5 | Single | B-C | Ⅲ-Ⅳ | No | 2 | Moderate | High | 1 | Weak | Low | Low | 107.0 | Censored |
| 62 | 71 | Male | No | > 5 | Multiple | B-C | Ⅲ-Ⅳ | Yes | 2 | Moderate | High | 3 | Strong | High | Low | 107.0 | Censored |
| 63 | 52 | Male | Yes | <= 5 | Single | B-C | Ⅲ-Ⅳ | No | 2 | Moderate | High | 1 | Weak | Low | High | 59.0 | Die |
| 64 | 56 | Male | Yes | <= 5 | Single | 0-A | Ⅰ-Ⅱ | Yes | 3 | Strong | High | 2 | Moderate | High | High | 107.0 | Censored |
| 65 | 57 | Male | Yes | <= 5 | Single | 0-A | Ⅰ-Ⅱ | No | 3 | Strong | High | 0 | Negative | Low | High | 107.0 | Censored |
| 66 | 43 | Male | Yes | <= 5 | Single | B-C | Ⅲ-Ⅳ | No | 0 | Negative | Low | 0 | Negative | Low | Low | 107.0 | Censored |
| 67 | 56 | Male | Yes | <= 5 | Single | 0-A | Ⅰ-Ⅱ | No | 0 | Negative | Low | 0 | Negative | Low | Low | 107.0 | Censored |
| 68 | 49 | Male | Yes | <= 5 | Multiple | B-C | Ⅲ-Ⅳ | No | 0 | Negative | Low | 0 | Negative | Low | Low | 16.0 | Die |
| 69 | 70 | Male | Yes | > 5 | Single | B-C | Ⅰ-Ⅱ | No | 0 | Negative | Low | 0 | Negative | Low | Low | 30.0 | Die |
| 70 | 59 | Male | Yes | > 5 | Multiple | B-C | Ⅲ-Ⅳ | No | 0 | Negative | Low | 0 | Negative | Low | Low | 10.0 | Die |
| 71 | 36 | Male | Yes | <= 5 | Single | 0-A | Ⅰ-Ⅱ | Yes | 2 | Moderate | High | 1 | Weak | Low | Low | 107.0 | Censored |
| 72 | 62 | Female | Yes | <= 5 | Single | 0-A | Ⅰ-Ⅱ | Yes | 1 | Weak | Low | 0 | Negative | Low | Low | 29.0 | Die |
| 73 | 74 | Male | No | > 5 | Single | B-C | Ⅲ-Ⅳ | No | 1 | Weak | Low | 0 | Negative | Low | Low | 10.0 | Die |
| 74 | 63 | Male | Yes | > 5 | Single | B-C | Ⅲ-Ⅳ | No | 1 | Weak | Low | 0 | Negative | Low | Low | 106.0 | Censored |
| 75 | 55 | Female | Yes | > 5 | Multiple | B-C | Ⅲ-Ⅳ | No | 1 | Weak | Low | 0 | Negative | Low | Low | 9.0 | Die |
| 76 | 44 | Male | Yes | <= 5 | Single | B-C | Ⅲ-Ⅳ | No | 1 | Weak | Low | 0 | Negative | Low | Low | 9.0 | Die |
| 77 | 73 | Male | Yes | > 5 | Single | B-C | Ⅰ-Ⅱ | Yes | 1 | Weak | Low | 0 | Negative | Low | Low | 106.0 | Censored |
| 78 | 50 | Male | No | <= 5 | Single | 0-A | Ⅰ-Ⅱ | No | 1 | Weak | Low | 0 | Negative | Low | Low | 25.0 | Die |
| 79 | 37 | Male | Yes | > 5 | Multiple | B-C | Ⅲ-Ⅳ | Yes | 1 | Weak | Low | 1 | Weak | Low | High | 2.0 | Die |
| 80 | 41 | Male | Yes | > 5 | Single | B-C | Ⅰ-Ⅱ | No | 0 | Negative | Low | 0 | Negative | Low | High | 23.0 | Die |
| 81 | 52 | Male | Yes | <= 5 | Single | 0-A | Ⅰ-Ⅱ | Yes | 1 | Weak | Low | 0 | Negative | Low | High | 106.0 | Censored |
| 82 | 60 | Male | No | <= 5 | Single | B-C | Ⅲ-Ⅳ | No | 1 | Weak | Low | 0 | Negative | Low | High | 29.0 | Die |
| 83 | 70 | Male | Yes | > 5 | Single | B-C | Ⅰ-Ⅱ | Yes | 1 | Weak | Low | 0 | Negative | Low | Low | 20.0 | Die |
| 84 | 65 | Female | No | <= 5 | Single | 0-A | Ⅰ-Ⅱ | No | 1 | Weak | Low | 1 | Weak | Low | Low | 36.0 | Die |
| 85 | 43 | Male | Yes | <= 5 | Single | B-C | Ⅲ-Ⅳ | No | 0 | Negative | Low | 0 | Negative | Low | High | 34.0 | Die |
| 86 | 71 | Female | Yes | > 5 | Single | B-C | Ⅰ-Ⅱ | No | 1 | Weak | Low | 2 | Moderate | High | Low | 106.0 | Censored |
| 87 | 52 | Male | Yes | <= 5 | Single | B-C | Ⅲ-Ⅳ | Yes | 2 | Moderate | High | 2 | Moderate | High | Low | 106.0 | Censored |
| 88 | 53 | Male | Yes | <= 5 | Single | B-C | Ⅲ-Ⅳ | No | 1 | Weak | Low | 1 | Weak | Low | Low | 22.0 | Censored |
| 89 | 58 | Male | Yes | > 5 | Single | B-C | Ⅰ-Ⅱ | No | 1 | Weak | Low | 0 | Negative | Low | Low | 106.0 | Censored |
| 90 | 49 | Male | Yes | <= 5 | Single | 0-A | Ⅰ-Ⅱ | Yes | 1 | Weak | Low | 0 | Negative | Low | Low | 46.0 | Die |
| 91 | 51 | Male | Yes | > 5 | Single | B-C | Ⅰ-Ⅱ | No | 0 | Negative | Low | 1 | Weak | Low | Low | 106.0 | Censored |
| 92 | 49 | Female | Yes | <= 5 | Single | 0-A | Ⅰ-Ⅱ | No | 1 | Weak | Low | 0 | Negative | Low | High | 25.0 | Die |
| 93 | 55 | Female | Yes | <= 5 | Single | 0-A | Ⅰ-Ⅱ | Yes | 1 | Weak | Low | 0 | Negative | Low | High | 26.0 | Die |
| 94 | 42 | Male | Yes | > 5 | Single | B-C | Ⅰ-Ⅱ | No | 2 | Moderate | High | 1 | Weak | Low | High | 106.0 | Censored |
| 95 | 48 | Male | Yes | > 5 | Multiple | B-C | Ⅲ-Ⅳ | No | 3 | Strong | High | 2 | Moderate | High | Low | 10.0 | Die |
| 96 | 52 | Female | Yes | <= 5 | Single | 0-A | Ⅰ-Ⅱ | Yes | 1 | Weak | Low | 1 | Weak | Low | Low | 82.0 | Censored |
| 97 | 54 | Male | Yes | > 5 | Single | B-C | Ⅰ-Ⅱ | Yes | 1 | Weak | Low | 1 | Weak | Low | Low | 48.1 | Die |
| 98 | 37 | Male | Yes | <= 5 | Single | 0-A | Ⅰ-Ⅱ | Yes | 1 | Weak | Low | 1 | Weak | Low | Low | 11.0 | Die |
| 99 | 60 | Male | Yes | <= 5 | Single | 0-A | Ⅰ-Ⅱ | No | 1 | Weak | Low | 0 | Negative | Low | Low | 36.0 | Die |
| 100 | 44 | Male | No | <= 5 | Single | 0-A | Ⅰ-Ⅱ | No | 1 | Weak | Low | 1 | Weak | Low | Low | 11.0 | Die |
| 101 | 50 | Male | Yes | > 5 | Multiple | B-C | Ⅲ-Ⅳ | No | 1 | Weak | Low | 0 | Negative | Low | Low | 19.0 | Die |
| 102 | 52 | Female | No | <= 5 | Single | 0-A | Ⅰ-Ⅱ | Yes | 2 | Moderate | High | 0 | Negative | Low | Low | 34.0 | Die |
| 103 | 58 | Male | Yes | > 5 | Single | B-C | Ⅰ-Ⅱ | Yes | 2 | Moderate | High | 1 | Weak | Low | Low | 106.0 | Censored |
| 104 | 40 | Male | Yes | <= 5 | Single | 0-A | Ⅰ-Ⅱ | No | 2 | Moderate | High | 1 | Weak | Low | Low | 106.0 | Censored |
| 105 | 49 | Female | No | > 5 | Single | B-C | Ⅰ-Ⅱ | No | 0 | Negative | Low | 1 | Weak | Low | Low | 32.0 | Die |
| 106 | 58 | Female | Yes | <= 5 | Single | B-C | Ⅲ-Ⅳ | No | 1 | Weak | Low | 0 | Negative | Low | High | 106.0 | Censored |
| 107 | 60 | Female | Yes | <= 5 | Single | B-C | Ⅲ-Ⅳ | Yes | 2 | Moderate | High | 0 | Negative | Low | High | 22.0 | Die |
| 108 | 60 | Male | Yes | <= 5 | Single | 0-A | Ⅰ-Ⅱ | No | 2 | Moderate | High | 0 | Negative | Low | Low | 106.0 | Censored |
| 109 | 58 | Male | Yes | <= 5 | Single | 0-A | Ⅰ-Ⅱ | No | 2 | Moderate | High | 1 | Weak | Low | Low | 106.0 | Censored |
| 110 | 68 | Female | Yes | <= 5 | Single | 0-A | Ⅰ-Ⅱ | Yes | 2 | Moderate | High | 1 | Weak | Low | Low | 46.0 | Die |
| 111 | 45 | Male | Yes | > 5 | Single | B-C | Ⅲ-Ⅳ | No | 1 | Weak | Low | 0 | Negative | Low | Low | 34.0 | Die |
| 112 | 53 | Female | Yes | > 5 | Single | B-C | Ⅲ-Ⅳ | No | 0 | Negative | Low | 0 | Negative | Low | High | 22.0 | Die |
| 113 | 66 | Male | Yes | <= 5 | Single | 0-A | Ⅰ-Ⅱ | Yes | 2 | Moderate | High | 0 | Negative | Low | Low | 106.0 | Censored |
| 114 | 43 | Male | Yes | <= 5 | Single | 0-A | Ⅰ-Ⅱ | No | 2 | Moderate | High | 0 | Negative | Low | Low | 106.0 | Censored |
| 115 | 35 | Male | Yes | > 5 | Single | B-C | Ⅰ-Ⅱ | No | 1 | Weak | Low | 0 | Negative | Low | High | 106.0 | Censored |
| 116 | 56 | Male | Yes | <= 5 | Single | 0-A | Ⅰ-Ⅱ | No | 2 | Moderate | High | 1 | Weak | Low | High | 82.0 | Censored |
| 117 | 59 | Male | Yes | > 5 | Single | B-C | Ⅰ-Ⅱ | No | 2 | Moderate | High | 0 | Negative | Low | Low | 49.0 | Die |
| 118 | 60 | Male | Yes | > 5 | Single | B-C | Ⅰ-Ⅱ | Yes | 2 | Moderate | High | 1 | Weak | Low | Low | 16.0 | Die |
| 119 | 62 | Male | Yes | <= 5 | Single | 0-A | Ⅰ-Ⅱ | No | 3 | Strong | High | 1 | Weak | Low | High | 36.0 | Die |
| 120 | 37 | Male | Yes | <= 5 | Single | 0-A | Ⅰ-Ⅱ | No | 3 | Strong | High | 1 | Weak | Low | Low | 22.0 | Censored |
| 121 | 50 | Male | Yes | > 5 | Single | B-C | Ⅰ-Ⅱ | Yes | 2 | Moderate | High | 0 | Negative | Low | Low | 42.2 | Die |
| 122 | 52 | Male | No | <= 5 | Single | B-C | Ⅲ-Ⅳ | No | 1 | Weak | Low | 0 | Negative | Low | Low | 69.0 | Censored |
| 123 | 63 | Male | Yes | <= 5 | Single | B-C | Ⅲ-Ⅳ | No | 1 | Weak | Low | 0 | Negative | Low | High | 58.0 | Die |
| 124 | 62 | Male | No | <= 5 | Single | 0-A | Ⅰ-Ⅱ | Yes | 1 | Weak | Low | 0 | Negative | Low | Low | 105.0 | Censored |
| 125 | 43 | Male | Yes | > 5 | Single | B-C | Ⅰ-Ⅱ | Yes | 0 | Negative | Low | 0 | Negative | Low | Low | 57.0 | Censored |
| 126 | 70 | Male | Yes | > 5 | Single | B-C | Ⅲ-Ⅳ | Yes | 1 | Weak | Low | 0 | Negative | Low | Low | 92.0 | Die |
| 127 | 68 | Male | No | > 5 | Single | B-C | Ⅲ-Ⅳ | Yes | 0 | Negative | Low | 1 | Weak | Low | Low | 41.0 | Die |
| 128 | 51 | Female | Yes | <= 5 | Single | 0-A | Ⅰ-Ⅱ | No | 2 | Moderate | High | 1 | Weak | Low | Low | 105.0 | Censored |
| 129 | 38 | Male | Yes | > 5 | Single | B-C | Ⅲ-Ⅳ | Yes | 2 | Moderate | High | 1 | Weak | Low | Low | 20.0 | Die |
| 130 | 45 | Male | Yes | <= 5 | Single | B-C | Ⅲ-Ⅳ | Yes | 2 | Moderate | High | 1 | Weak | Low | High | 14.0 | Die |
| 131 | 59 | Female | No | > 5 | Single | B-C | Ⅰ-Ⅱ | Yes | 2 | Moderate | High | 2 | Moderate | High | Low | 41.0 | Die |
| 132 | 76 | Male | Yes | <= 5 | Single | 0-A | Ⅰ-Ⅱ | No | 0 | Negative | Low | 0 | Negative | Low | Low | 16.0 | Die |
| 133 | 35 | Male | Yes | <= 5 | Single | 0-A | Ⅰ-Ⅱ | No | 1 | Weak | Low | 0 | Negative | Low | Low | 105.0 | Censored |
| 134 | 50 | Male | Yes | <= 5 | Single | 0-A | Ⅰ-Ⅱ | No | 1 | Weak | Low | 0 | Negative | Low | Low | 105.0 | Censored |
| 135 | 38 | Female | Yes | <= 5 | Single | B-C | Ⅲ-Ⅳ | Yes | 0 | Negative | Low | 0 | Negative | Low | Low | 30.0 | Die |
| 136 | 35 | Male | Yes | > 5 | Single | B-C | Ⅲ-Ⅳ | No | 1 | Weak | Low | 0 | Negative | Low | Low | 105.0 | Censored |
| 137 | 56 | Male | Yes | > 5 | Single | B-C | Ⅲ-Ⅳ | No | 1 | Weak | Low | 1 | Weak | Low | Low | 18.0 | Die |
| 138 | 46 | Male | Yes | > 5 | Single | B-C | Ⅲ-Ⅳ | Yes | 1 | Weak | Low | 0 | Negative | Low | Low | 8.0 | Die |
| 139 | 49 | Male | Yes | <= 5 | Single | B-C | Ⅲ-Ⅳ | No | 1 | Weak | Low | 1 | Weak | Low | Low | 105.0 | Censored |
| 140 | 35 | Male | Yes | <= 5 | Single | 0-A | Ⅰ-Ⅱ | No | 1 | Weak | Low | 0 | Negative | Low | High | 105.0 | Censored |
| 141 | 36 | Male | Yes | > 5 | Single | B-C | Ⅰ-Ⅱ | No | 1 | Weak | Low | 1 | Weak | Low | Low | 14.0 | Die |
| 142 | 62 | Male | No | <= 5 | Single | B-C | Ⅲ-Ⅳ | Yes | 2 | Moderate | High | 1 | Weak | Low | Low | 52.0 | Die |
| 143 | 54 | Male | No | <= 5 | Multiple | B-C | Ⅲ-Ⅳ | No | 1 | Weak | Low | 0 | Negative | Low | Low | 105.0 | Censored |
| 144 | 44 | Female | Yes | > 5 | Single | B-C | Ⅲ-Ⅳ | No | 1 | Weak | Low | 0 | Negative | Low | Low | 16.0 | Die |
| 145 | 48 | Male | Yes | <= 5 | Single | B-C | Ⅲ-Ⅳ | Yes | 1 | Weak | Low | 0 | Negative | Low | High | 105.0 | Censored |
| 146 | 48 | Male | Yes | <= 5 | Single | 0-A | Ⅰ-Ⅱ | Yes | 1 | Weak | Low | 0 | Negative | Low | Low | 105.0 | Censored |
| 147 | 75 | Male | Yes | > 5 | Single | B-C | Ⅲ-Ⅳ | Yes | 1 | Weak | Low | 1 | Weak | Low | High | 9.0 | Die |
| 148 | 67 | Male | Yes | > 5 | Single | B-C | Ⅰ-Ⅱ | No | 1 | Weak | Low | 0 | Negative | Low | Low | 105.0 | Censored |
| 149 | 50 | Male | No | <= 5 | Single | 0-A | Ⅰ-Ⅱ | No | 1 | Weak | Low | 1 | Weak | Low | High | 105.0 | Censored |
| 150 | 57 | Male | No | > 5 | Multiple | B-C | Ⅲ-Ⅳ | Yes | 1 | Weak | Low | 0 | Negative | Low | Low | 32.0 | Die |
| 151 | 59 | Male | No | > 5 | Multiple | B-C | Ⅲ-Ⅳ | Yes | 2 | Moderate | High | 1 | Weak | Low | High | 64.0 | Die |
| 152 | 58 | Male | Yes | > 5 | Single | B-C | Ⅰ-Ⅱ | No | 2 | Moderate | High | 1 | Weak | Low | High | 34.0 | Die |
| 153 | 46 | Male | Yes | <= 5 | Single | 0-A | Ⅰ-Ⅱ | No | 2 | Moderate | High | 1 | Weak | Low | Low | 80.0 | Censored |
| 154 | 41 | Female | Yes | <= 5 | Multiple | 0-A | Ⅲ-Ⅳ | Yes | 1 | Weak | Low | 0 | Negative | Low | Low | 104.0 | Censored |
| 155 | 53 | Male | Yes | <= 5 | Single | 0-A | Ⅰ-Ⅱ | No | 1 | Weak | Low | 0 | Negative | Low | Low | 80.0 | Censored |
| 156 | 55 | Male | Yes | <= 5 | Single | 0-A | Ⅰ-Ⅱ | No | 1 | Weak | Low | 0 | Negative | Low | Low | 80.0 | Censored |
| 157 | 65 | Male | Yes | <= 5 | Single | 0-A | Ⅰ-Ⅱ | Yes | 1 | Weak | Low | 0 | Negative | Low | Low | 90.0 | Die |
| 158 | 52 | Female | No | <= 5 | Single | 0-A | Ⅰ-Ⅱ | No | 1 | Weak | Low | 0 | Negative | Low | High | 104.0 | Censored |
| 159 | 56 | Male | Yes | <= 5 | Multiple | B-C | Ⅲ-Ⅳ | No | 1 | Weak | Low | 0 | Negative | Low | Low | 57.0 | Censored |
| 160 | 59 | Male | Yes | <= 5 | Single | 0-A | Ⅰ-Ⅱ | Yes | 1 | Weak | Low | 0 | Negative | Low | Low | 37.0 | Die |
| 161 | 53 | Female | Yes | <= 5 | Single | 0-A | Ⅰ-Ⅱ | No | 1 | Weak | Low | 0 | Negative | Low | High | 104.0 | Censored |
| 162 | 43 | Male | Yes | <= 5 | Single | 0-A | Ⅰ-Ⅱ | No | 2 | Moderate | High | 0 | Negative | Low | Low | 104.0 | Censored |
| 163 | 56 | Male | Yes | > 5 | Single | B-C | Ⅲ-Ⅳ | Yes | 1 | Weak | Low | 1 | Weak | Low | Low | 6.5 | Die |
| 164 | 48 | Male | No | > 5 | Single | B-C | Ⅰ-Ⅱ | No | 2 | Moderate | High | 0 | Negative | Low | High | 104.0 | Censored |
| 165 | 54 | Female | No | > 5 | Single | B-C | Ⅰ-Ⅱ | No | 1 | Weak | Low | 0 | Negative | Low | Low | 104.0 | Censored |
| 166 | 49 | Male | No | <= 5 | Single | 0-A | Ⅰ-Ⅱ | Yes | 1 | Weak | Low | 0 | Negative | Low | High | 7.0 | Die |
| 167 | 78 | Male | Yes | > 5 | Multiple | B-C | Ⅲ-Ⅳ | No | 1 | Weak | Low | 0 | Negative | Low | Low | 78.0 | Die |
| 168 | 57 | Male | Yes | > 5 | Multiple | B-C | Ⅲ-Ⅳ | Yes | 1 | Weak | Low | 0 | Negative | Low | Low | 27.0 | Die |
| 169 | 32 | Female | Yes | <= 5 | Single | 0-A | Ⅰ-Ⅱ | No | 1 | Weak | Low | 0 | Negative | Low | Low | 104.0 | Censored |
| 170 | 45 | Male | Yes | <= 5 | Multiple | B-C | Ⅲ-Ⅳ | No | 1 | Weak | Low | 0 | Negative | Low | High | 104.0 | Censored |
| 171 | 59 | Male | No | > 5 | Single | B-C | Ⅲ-Ⅳ | No | 2 | Moderate | High | 0 | Negative | Low | High | 104.0 | Censored |
| 172 | 55 | Male | No | <= 5 | Single | 0-A | Ⅰ-Ⅱ | Yes | 0 | Negative | Low | 1 | Weak | Low | Low | 90.0 | Die |
| 173 | 61 | Male | No | <= 5 | Single | 0-A | Ⅰ-Ⅱ | Yes | 2 | Moderate | High | 1 | Weak | Low | Low | 49.0 | Die |
| 174 | 59 | Male | No | <= 5 | Single | 0-A | Ⅰ-Ⅱ | No | 2 | Moderate | High | 0 | Negative | Low | Low | 80.0 | Censored |
| 175 | 75 | Male | Yes | <= 5 | Single | B-C | Ⅲ-Ⅳ | Yes | 1 | Weak | Low | 0 | Negative | Low | Low | 47.0 | Die |
| 176 | 54 | Male | Yes | > 5 | Single | B-C | Ⅰ-Ⅱ | No | 1 | Weak | Low | 1 | Weak | Low | Low | 104.0 | Censored |
| 177 | 42 | Male | Yes | <= 5 | Single | 0-A | Ⅰ-Ⅱ | No | 1 | Weak | Low | 1 | Weak | Low | High | 104.0 | Censored |
| 178 | 38 | Male | No | <= 5 | Single | 0-A | Ⅰ-Ⅱ | Yes | 2 | Moderate | High | 1 | Weak | Low | High | 104.0 | Censored |
| 179 | 27 | Male | Yes | > 5 | Single | B-C | Ⅲ-Ⅳ | Yes | 1 | Weak | Low | 1 | Weak | Low | High | 52.0 | Die |
| 180 | 51 | Male | No | <= 5 | Single | 0-A | Ⅰ-Ⅱ | Yes | 3 | Strong | High | 1 | Weak | Low | Low | 62.0 | Die |
| 181 | 36 | Male | Yes | <= 5 | Single | 0-A | Ⅰ-Ⅱ | No | 3 | Strong | High | 1 | Weak | Low | High | 104.0 | Censored |
| 182 | 33 | Male | No | <= 5 | Single | B-C | Ⅲ-Ⅳ | No | 2 | Moderate | High | 2 | Moderate | High | High | 34.0 | Censored |
| 183 | 72 | Male | Yes | <= 5 | Single | 0-A | Ⅰ-Ⅱ | No | 3 | Strong | High | 1 | Weak | Low | High | 104.0 | Censored |
| 184 | 61 | Male | Yes | <= 5 | Single | 0-A | Ⅰ-Ⅱ | No | 1 | Weak | Low | 1 | Weak | Low | Low | 44.0 | Censored |
| 185 | 46 | Male | Yes | <= 5 | Single | B-C | Ⅲ-Ⅳ | Yes | 0 | Negative | Low | 1 | Weak | Low | Low | 79.0 | Die |
| 186 | 48 | Male | Yes | <= 5 | Multiple | B-C | Ⅲ-Ⅳ | No | 2 | Moderate | High | 2 | Moderate | High | High | 103.0 | Censored |
| 187 | 63 | Male | Yes | <= 5 | Single | 0-A | Ⅰ-Ⅱ | Yes | 2 | Moderate | High | 1 | Weak | Low | Low | 81.0 | Die |
| 188 | 75 | Male | Yes | > 5 | Single | B-C | Ⅲ-Ⅳ | Yes | 1 | Weak | Low | 1 | Weak | Low | Low | 103.0 | Censored |
| 189 | 52 | Male | Yes | <= 5 | Single | 0-A | Ⅰ-Ⅱ | No | 2 | Moderate | High | 1 | Weak | Low | High | 103.0 | Censored |
| 190 | 71 | Female | Yes | <= 5 | Single | 0-A | Ⅰ-Ⅱ | Yes | 1 | Weak | Low | 2 | Moderate | High | Low | 83.0 | Die |
| 191 | 59 | Male | Yes | > 5 | Single | B-C | Ⅲ-Ⅳ | Yes | 1 | Weak | Low | 1 | Weak | Low | Low | 8.0 | Die |
| 192 | 59 | Male | Yes | > 5 | Multiple | B-C | Ⅲ-Ⅳ | Yes | 2 | Moderate | High | 1 | Weak | Low | Low | 53.0 | Die |
| 193 | 56 | Female | Yes | > 5 | Single | B-C | Ⅰ-Ⅱ | Yes | 1 | Weak | Low | 1 | Weak | Low | Low | 12.0 | Die |
| 194 | 63 | Male | Yes | > 5 | Single | B-C | Ⅰ-Ⅱ | No | 3 | Strong | High | 0 | Negative | Low | High | 11.0 | Die |
| 195 | 51 | Male | Yes | > 5 | Single | B-C | Ⅲ-Ⅳ | No | 3 | Strong | High | 0 | Negative | Low | Low | 103.0 | Censored |
| 196 | 56 | Female | Yes | <= 5 | Single | 0-A | Ⅰ-Ⅱ | No | 2 | Moderate | High | 1 | Weak | Low | High | 103.0 | Censored |
| 197 | 51 | Male | Yes | <= 5 | Single | 0-A | Ⅰ-Ⅱ | Yes | 1 | Weak | Low | 2 | Moderate | High | Low | 35.0 | Die |
| 198 | 41 | Female | Yes | > 5 | Single | B-C | Ⅰ-Ⅱ | No | 2 | Moderate | High | 0 | Negative | Low | High | 103.0 | Censored |
| 199 | 71 | Male | Yes | <= 5 | Multiple | B-C | Ⅲ-Ⅳ | Yes | 2 | Moderate | High | 2 | Moderate | High | Low | 32.0 | Die |
| 200 | 50 | Male | No | <= 5 | Single | 0-A | Ⅰ-Ⅱ | No | 2 | Moderate | High | 1 | Weak | Low | Low | 103.0 | Censored |
| 201 | 42 | Male | Yes | <= 5 | Single | 0-A | Ⅰ-Ⅱ | No | 3 | Strong | High | 1 | Weak | Low | High | 79.0 | Censored |
| 202 | 53 | Male | No | <= 5 | Single | 0-A | Ⅰ-Ⅱ | Yes | 3 | Strong | High | 1 | Weak | Low | Low | 103.0 | Censored |
| 203 | 65 | Male | No | <= 5 | Single | B-C | Ⅲ-Ⅳ | Yes | 2 | Moderate | High | 1 | Weak | Low | Low | 19.0 | Die |
| 204 | 54 | Female | Yes | > 5 | Single | B-C | Ⅰ-Ⅱ | No | 2 | Moderate | High | 2 | Moderate | High | High | 103.0 | Censored |
| 205 | 41 | Male | Yes | > 5 | Single | B-C | Ⅲ-Ⅳ | Yes | 3 | Strong | High | 1 | Weak | Low | Low | 18.0 | Die |
| 206 | 52 | Male | Yes | <= 5 | Single | 0-A | Ⅰ-Ⅱ | No | 3 | Strong | High | 1 | Weak | Low | High | 103.0 | Censored |
| 207 | 58 | Male | No | > 5 | Single | B-C | Ⅲ-Ⅳ | Yes | 2 | Moderate | High | 1 | Weak | Low | High | 19.0 | Die |
| 208 | 32 | Male | Yes | <= 5 | Single | 0-A | Ⅰ-Ⅱ | No | 3 | Strong | High | 1 | Weak | Low | High | 103.0 | Censored |
| 209 | 55 | Male | No | <= 5 | Single | 0-A | Ⅰ-Ⅱ | No | 3 | Strong | High | 2 | Moderate | High | High | 103.0 | Censored |
| 210 | 52 | Male | Yes | <= 5 | Single | 0-A | Ⅰ-Ⅱ | No | 3 | Strong | High | 2 | Moderate | High | High | 103.0 | Censored |
| 211 | 52 | Male | Yes | <= 5 | Single | 0-A | Ⅰ-Ⅱ | No | 3 | Strong | High | 2 | Moderate | High | High | 96.0 | Die |
| 212 | 76 | Male | Yes | > 5 | Single | B-C | Ⅰ-Ⅱ | Yes | 3 | Strong | High | 2 | Moderate | High | Low | 79.0 | Censored |
| 213 | 55 | Male | Yes | > 5 | Single | B-C | Ⅲ-Ⅳ | Yes | 2 | Moderate | High | 0 | Negative | Low | Low | 65.0 | Die |
| 214 | 57 | Male | Yes | > 5 | Single | B-C | Ⅲ-Ⅳ | Yes | 2 | Moderate | High | 3 | Strong | High | High | 79.0 | Die |
| 215 | 49 | Male | Yes | <= 5 | Single | 0-A | Ⅰ-Ⅱ | No | 2 | Moderate | High | 1 | Weak | Low | High | 103.0 | Censored |
| 216 | 42 | Male | No | <= 5 | Single | 0-A | Ⅰ-Ⅱ | Yes | 3 | Strong | High | 2 | Moderate | High | High | 79.0 | Die |
| 217 | 50 | Male | Yes | > 5 | Single | B-C | Ⅰ-Ⅱ | No | 3 | Strong | High | 2 | Moderate | High | Low | 103.0 | Censored |
| 218 | 66 | Male | No | <= 5 | Single | 0-A | Ⅰ-Ⅱ | Yes | 3 | Strong | High | 1 | Weak | Low | High | 101.0 | Die |
| 219 | 60 | Male | Yes | <= 5 | Single | 0-A | Ⅰ-Ⅱ | No | 3 | Strong | High | 1 | Weak | Low | High | 102.0 | Censored |
| 220 | 55 | Male | Yes | <= 5 | Single | 0-A | Ⅰ-Ⅱ | No | 3 | Strong | High | 1 | Weak | Low | Low | 102.0 | Censored |
| 221 | 49 | Male | Yes | <= 5 | Single | 0-A | Ⅰ-Ⅱ | No | 1 | Weak | Low | 1 | Weak | Low | High | 102.0 | Censored |
| 222 | 28 | Male | No | <= 5 | Single | B-C | Ⅲ-Ⅳ | No | 2 | Moderate | High | 1 | Weak | Low | High | 42.0 | Die |
| 223 | 59 | Female | No | <= 5 | Multiple | 0-A | Ⅲ-Ⅳ | No | 3 | Strong | High | 1 | Weak | Low | Low | 78.0 | Censored |
| 224 | 53 | Male | Yes | <= 5 | Single | B-C | Ⅲ-Ⅳ | No | 3 | Strong | High | 1 | Weak | Low | Low | 102.0 | Censored |
| 225 | 51 | Male | Yes | > 5 | Multiple | B-C | Ⅲ-Ⅳ | No | 3 | Strong | High | 3 | Strong | High | High | 45.0 | Die |
| 226 | 55 | Male | No | <= 5 | Single | B-C | Ⅲ-Ⅳ | No | 2 | Moderate | High | 3 | Strong | High | Low | 102.0 | Censored |
| 227 | 65 | Male | Yes | <= 5 | Single | 0-A | Ⅰ-Ⅱ | Yes | 3 | Strong | High | 3 | Strong | High | Low | 27.0 | Die |
| 228 | 34 | Male | Yes | <= 5 | Single | 0-A | Ⅰ-Ⅱ | No | 2 | Moderate | High | 1 | Weak | Low | High | 102.0 | Censored |
| 229 | 51 | Female | No | <= 5 | Single | 0-A | Ⅰ-Ⅱ | Yes | 3 | Strong | High | 1 | Weak | Low | High | 84.0 | Die |
| 230 | 53 | Male | No | > 5 | Single | B-C | Ⅲ-Ⅳ | Yes | 0 | Negative | Low | 0 | Negative | Low | High | 8.0 | Die |
| 231 | 52 | Male | No | <= 5 | Single | B-C | Ⅲ-Ⅳ | Yes | 2 | Moderate | High | 2 | Moderate | High | High | 18.0 | Die |
| 232 | 32 | Male | Yes | > 5 | Single | B-C | Ⅲ-Ⅳ | Yes | 1 | Weak | Low | 0 | Negative | Low | Low | 29.0 | Die |
| 233 | 57 | Male | No | <= 5 | Single | 0-A | Ⅰ-Ⅱ | Yes | 1 | Weak | Low | 3 | Strong | High | Low | 48.8 | Die |
| 234 | 51 | Male | Yes | <= 5 | Single | B-C | Ⅲ-Ⅳ | No | 1 | Weak | Low | 1 | Weak | Low | Low | 102.0 | Censored |
| 235 | 58 | Male | Yes | > 5 | Single | B-C | Ⅰ-Ⅱ | No | 1 | Weak | Low | 0 | Negative | Low | High | 102.0 | Censored |
| 236 | 58 | Male | Yes | <= 5 | Single | 0-A | Ⅰ-Ⅱ | No | 1 | Weak | Low | 1 | Weak | Low | High | 32.0 | Censored |
| 237 | 73 | Female | Yes | <= 5 | Single | B-C | Ⅲ-Ⅳ | Yes | 1 | Weak | Low | 1 | Weak | Low | High | 88.0 | Die |
| 238 | 46 | Male | Yes | <= 5 | Single | B-C | Ⅲ-Ⅳ | Yes | 2 | Moderate | High | 1 | Weak | Low | Low | 73.0 | Die |
| 239 | 67 | Male | Yes | <= 5 | Single | 0-A | Ⅰ-Ⅱ | Yes | 2 | Moderate | High | 1 | Weak | Low | High | 18.0 | Die |
| 240 | 53 | Male | Yes | <= 5 | Single | B-C | Ⅲ-Ⅳ | No | 1 | Weak | Low | 0 | Negative | Low | High | 102.0 | Censored |
| 241 | 59 | Male | Yes | <= 5 | Single | 0-A | Ⅰ-Ⅱ | No | 1 | Weak | Low | 0 | Negative | Low | Low | 102.0 | Censored |
| 242 | 59 | Male | Yes | > 5 | Single | B-C | Ⅲ-Ⅳ | No | 1 | Weak | Low | 0 | Negative | Low | Low | 102.0 | Censored |
| 243 | 54 | Male | Yes | <= 5 | Single | 0-A | Ⅰ-Ⅱ | No | 1 | Weak | Low | 1 | Weak | Low | High | 102.0 | Censored |
| 244 | 52 | Female | Yes | <= 5 | Single | 0-A | Ⅰ-Ⅱ | No | 2 | Moderate | High | 1 | Weak | Low | High | 102.0 | Censored |
| 245 | 54 | Male | Yes | > 5 | Single | B-C | Ⅰ-Ⅱ | Yes | 1 | Weak | Low | 2 | Moderate | High | Low | 27.0 | Die |
| 246 | 57 | Male | Yes | > 5 | Multiple | B-C | Ⅲ-Ⅳ | No | 1 | Weak | Low | 0 | Negative | Low | High | 13.0 | Die |
| 247 | 46 | Male | No | <= 5 | Single | B-C | Ⅲ-Ⅳ | Yes | 1 | Weak | Low | 1 | Weak | Low | Low | 27.0 | Die |
| 248 | 41 | Male | Yes | <= 5 | Single | 0-A | Ⅰ-Ⅱ | Yes | 1 | Weak | Low | 0 | Negative | Low | Low | 56.0 | Die |
| 249 | 61 | Female | No | <= 5 | Single | 0-A | Ⅰ-Ⅱ | No | 2 | Moderate | High | 1 | Weak | Low | Low | 102.0 | Censored |
| 250 | 48 | Male | Yes | <= 5 | Single | 0-A | Ⅰ-Ⅱ | Yes | 1 | Weak | Low | 1 | Weak | Low | Low | 86.0 | Die |
| 251 | 50 | Male | Yes | <= 5 | Multiple | B-C | Ⅲ-Ⅳ | No | 1 | Weak | Low | 1 | Weak | Low | Low | 96.0 | Die |
| 252 | 39 | Male | Yes | <= 5 | Single | 0-A | Ⅰ-Ⅱ | Yes | 2 | Moderate | High | 1 | Weak | Low | Low | 10.0 | Die |
| 253 | 64 | Male | No | <= 5 | Single | 0-A | Ⅰ-Ⅱ | Yes | 2 | Moderate | High | 1 | Weak | Low | Low | 99.0 | Die |
| 254 | 52 | Male | Yes | <= 5 | Single | 0-A | Ⅰ-Ⅱ | Yes | 1 | Weak | Low | 1 | Weak | Low | Low | 73.0 | Die |
| 255 | 43 | Male | Yes | <= 5 | Single | 0-A | Ⅰ-Ⅱ | Yes | 2 | Moderate | High | 2 | Moderate | High | Low | 12.0 | Die |
| 256 | 60 | Male | Yes | > 5 | Single | B-C | Ⅰ-Ⅱ | No | 2 | Moderate | High | 0 | Negative | Low | Low | 18.0 | Die |
| 257 | 58 | Male | Yes | <= 5 | Multiple | 0-A | Ⅲ-Ⅳ | No | 3 | Strong | High | 2 | Moderate | High | Low | 101.0 | Censored |
| 258 | 35 | Male | No | > 5 | Single | B-C | Ⅰ-Ⅱ | No | 2 | Moderate | High | 2 | Moderate | High | High | 101.0 | Censored |
| 259 | 58 | Male | Yes | <= 5 | Single | 0-A | Ⅰ-Ⅱ | No | 2 | Moderate | High | 1 | Weak | Low | High | 101.0 | Censored |
| 260 | 41 | Female | Yes | > 5 | Single | B-C | Ⅰ-Ⅱ | No | 3 | Strong | High | 1 | Weak | Low | Low | 101.0 | Censored |
| 261 | 54 | Male | Yes | > 5 | Single | B-C | Ⅰ-Ⅱ | No | 3 | Strong | High | 1 | Weak | Low | Low | 44.0 | Censored |
| 262 | 51 | Male | No | <= 5 | Single | 0-A | Ⅰ-Ⅱ | No | 2 | Moderate | High | 1 | Weak | Low | Low | 31.0 | Die |
| 263 | 54 | Male | Yes | > 5 | Single | B-C | Ⅲ-Ⅳ | Yes | 2 | Moderate | High | 1 | Weak | Low | Low | 13.0 | Die |
| 264 | 40 | Male | Yes | > 5 | Single | B-C | Ⅰ-Ⅱ | No | 1 | Weak | Low | 1 | Weak | Low | High | 5.0 | Die |
| 265 | 43 | Male | No | <= 5 | Single | B-C | Ⅲ-Ⅳ | Yes | 2 | Moderate | High | 2 | Moderate | High | Low | 46.0 | Die |
| 266 | 66 | Male | Yes | > 5 | Single | B-C | Ⅰ-Ⅱ | No | 2 | Moderate | High | 1 | Weak | Low | High | 101.0 | Censored |
| 267 | 50 | Male | No | > 5 | Single | B-C | Ⅲ-Ⅳ | No | 1 | Weak | Low | 1 | Weak | Low | High | 13.0 | Die |
| 268 | 56 | Male | Yes | > 5 | Single | B-C | Ⅰ-Ⅱ | Yes | 3 | Strong | High | 3 | Strong | High | Low | 101.0 | Censored |
| 269 | 51 | Male | Yes | <= 5 | Single | 0-A | Ⅰ-Ⅱ | No | 1 | Weak | Low | 2 | Moderate | High | Low | 101.0 | Censored |
| 270 | 38 | Male | No | > 5 | Single | B-C | Ⅰ-Ⅱ | No | 3 | Strong | High | 1 | Weak | Low | High | 101.0 | Censored |
| 271 | 63 | Male | Yes | <= 5 | Single | 0-A | Ⅰ-Ⅱ | Yes | 2 | Moderate | High | 3 | Strong | High | Low | 36.0 | Die |
| 272 | 62 | Male | Yes | <= 5 | Single | B-C | Ⅲ-Ⅳ | Yes | 1 | Weak | Low | 2 | Moderate | High | Low | 4.0 | Die |
| 273 | 50 | Male | Yes | > 5 | Single | B-C | Ⅰ-Ⅱ | Yes | 3 | Strong | High | 1 | Weak | Low | Low | 75.0 | Die |
| 274 | 63 | Female | Yes | > 5 | Multiple | B-C | Ⅲ-Ⅳ | Yes | 3 | Strong | High | 2 | Moderate | High | Low | 4.0 | Die |
| 275 | 46 | Male | No | <= 5 | Single | 0-A | Ⅰ-Ⅱ | No | 2 | Moderate | High | 2 | Moderate | High | Low | 43.0 | Die |
| 276 | 40 | Female | No | > 5 | Single | B-C | Ⅲ-Ⅳ | No | 3 | Strong | High | 2 | Moderate | High | Low | 101.0 | Censored |
| 277 | 48 | Male | Yes | > 5 | Single | B-C | Ⅰ-Ⅱ | Yes | 2 | Moderate | High | 1 | Weak | Low | High | 7.0 | Die |
| 278 | 62 | Male | Yes | <= 5 | Single | 0-A | Ⅰ-Ⅱ | No | 3 | Strong | High | 3 | Strong | High | High | 101.0 | Censored |
| 279 | 53 | Female | Yes | <= 5 | Single | 0-A | Ⅰ-Ⅱ | No | 3 | Strong | High | 3 | Strong | High | Low | 101.0 | Censored |
| 280 | 18 | Male | Yes | > 5 | Single | B-C | Ⅲ-Ⅳ | No | 3 | Strong | High | 1 | Weak | Low | Low | 101.0 | Censored |
| 281 | 40 | Male | Yes | > 5 | Single | B-C | Ⅰ-Ⅱ | Yes | 3 | Strong | High | 1 | Weak | Low | Low | 10.0 | Die |
| 282 | 52 | Male | Yes | > 5 | Single | B-C | Ⅰ-Ⅱ | No | 2 | Moderate | High | 1 | Weak | Low | Low | 101.0 | Censored |
| 283 | 59 | Male | No | <= 5 | Single | 0-A | Ⅰ-Ⅱ | No | 0 | Negative | Low | 0 | Negative | Low | Low | 101.0 | Censored |
| 284 | 49 | Male | Yes | > 5 | Multiple | B-C | Ⅲ-Ⅳ | Yes | 2 | Moderate | High | 1 | Weak | Low | Low | 9.0 | Die |
| 285 | 42 | Male | Yes | <= 5 | Single | B-C | Ⅲ-Ⅳ | Yes | 1 | Weak | Low | 1 | Weak | Low | Low | 19.0 | Die |
| 286 | 58 | Male | No | <= 5 | Multiple | B-C | Ⅲ-Ⅳ | No | 2 | Moderate | High | 1 | Weak | Low | Low | 21.0 | Die |
| 287 | 50 | Male | Yes | <= 5 | Multiple | B-C | Ⅲ-Ⅳ | Yes | 3 | Strong | High | 3 | Strong | High | Low | 38.0 | Die |
| 288 | 55 | Male | Yes | > 5 | Single | B-C | Ⅲ-Ⅳ | No | 3 | Strong | High | 0 | Negative | Low | Low | 35.0 | Die |
| 289 | 48 | Male | Yes | > 5 | Single | B-C | Ⅰ-Ⅱ | No | 3 | Strong | High | 2 | Moderate | High | Low | 101.0 | Censored |
| 290 | 59 | Male | Yes | <= 5 | Single | 0-A | Ⅰ-Ⅱ | Yes | 3 | Strong | High | 2 | Moderate | High | High | 23.0 | Die |
| 291 | 63 | Male | Yes | <= 5 | Single | 0-A | Ⅰ-Ⅱ | No | 3 | Strong | High | 3 | Strong | High | Low | 100.0 | Censored |
| 292 | 74 | Male | Yes | > 5 | Single | B-C | Ⅰ-Ⅱ | No | 3 | Strong | High | 2 | Moderate | High | Low | 76.0 | Censored |
| 293 | 60 | Male | Yes | > 5 | Single | B-C | Ⅲ-Ⅳ | Yes | 3 | Strong | High | 3 | Strong | High | Low | 16.0 | Die |
| 294 | 76 | Male | Yes | <= 5 | Single | 0-A | Ⅰ-Ⅱ | No | 2 | Moderate | High | 1 | Weak | Low | Low | 100.0 | Censored |
| 295 | 71 | Male | Yes | <= 5 | Single | 0-A | Ⅰ-Ⅱ | No | 2 | Moderate | High | 2 | Moderate | High | Low | 100.0 | Censored |
| 296 | 48 | Male | Yes | <= 5 | Single | B-C | Ⅲ-Ⅳ | No | 1 | Weak | Low | 1 | Weak | Low | Low | 9.0 | Die |
| 297 | 38 | Male | Yes | <= 5 | Single | 0-A | Ⅰ-Ⅱ | No | 1 | Weak | Low | 1 | Weak | Low | Low | 100.0 | Censored |
| 298 | 45 | Male | Yes | <= 5 | Single | 0-A | Ⅰ-Ⅱ | No | 1 | Weak | Low | 3 | Strong | High | Low | 100.0 | Censored |
| 299 | 44 | Male | Yes | <= 5 | Single | 0-A | Ⅰ-Ⅱ | No | 3 | Strong | High | 3 | Strong | High | Low | 100.0 | Censored |
| 300 | 68 | Male | Yes | > 5 | Multiple | B-C | Ⅲ-Ⅳ | Yes | 2 | Moderate | High | 1 | Weak | Low | Low | 28.0 | Die |
| 301 | 58 | Male | No | > 5 | Single | B-C | Ⅰ-Ⅱ | Yes | 3 | Strong | High | 2 | Moderate | High | Low | 58.0 | Die |
| 302 | 53 | Male | Yes | <= 5 | Single | 0-A | Ⅰ-Ⅱ | Yes | 3 | Strong | High | 2 | Moderate | High | Low | 100.0 | Censored |
| 303 | 39 | Male | Yes | <= 5 | Multiple | 0-A | Ⅲ-Ⅳ | No | 3 | Strong | High | 2 | Moderate | High | Low | 37.0 | Die |
| 304 | 52 | Male | Yes | > 5 | Single | B-C | Ⅰ-Ⅱ | Yes | 2 | Moderate | High | 1 | Weak | Low | Low | 64.0 | Censored |
| 305 | 57 | Male | Yes | > 5 | Single | B-C | Ⅰ-Ⅱ | Yes | 3 | Strong | High | 3 | Strong | High | Low | 100.0 | Censored |
| 306 | 43 | Male | Yes | > 5 | Single | B-C | Ⅲ-Ⅳ | No | 2 | Moderate | High | 1 | Weak | Low | Low | 100.0 | Censored |
| 307 | 26 | Female | Yes | <= 5 | Single | 0-A | Ⅰ-Ⅱ | No | 1 | Weak | Low | 1 | Weak | Low | Low | 100.0 | Censored |
| 308 | 72 | Male | Yes | <= 5 | Single | 0-A | Ⅰ-Ⅱ | Yes | 1 | Weak | Low | 1 | Weak | Low | Low | 100.0 | Censored |
| 309 | 49 | Male | Yes | <= 5 | Single | 0-A | Ⅰ-Ⅱ | Yes | 2 | Moderate | High | 2 | Moderate | High | High | 17.2 | Die |
| 310 | 44 | Male | Yes | <= 5 | Single | 0-A | Ⅰ-Ⅱ | No | 1 | Weak | Low | 2 | Moderate | High | Low | 41.0 | Censored |
| 311 | 38 | Male | No | <= 5 | Single | 0-A | Ⅰ-Ⅱ | Yes | 1 | Weak | Low | 0 | Negative | Low | Low | 18.4 | Die |
| 312 | 45 | Female | Yes | > 5 | Multiple | B-C | Ⅲ-Ⅳ | Yes | 1 | Weak | Low | 1 | Weak | Low | Low | 46.0 | Die |
| 313 | 35 | Male | Yes | <= 5 | Multiple | 0-A | Ⅲ-Ⅳ | Yes | 2 | Moderate | High | 2 | Moderate | High | Low | 48.0 | Die |
| 314 | 42 | Male | Yes | > 5 | Single | B-C | Ⅰ-Ⅱ | Yes | 1 | Weak | Low | 2 | Moderate | High | Low | 7.0 | Die |
| 315 | 70 | Male | Yes | > 5 | Single | B-C | Ⅰ-Ⅱ | Yes | 2 | Moderate | High | 2 | Moderate | High | High | 63.0 | Die |
| 316 | 59 | Male | Yes | <= 5 | Single | 0-A | Ⅰ-Ⅱ | No | 2 | Moderate | High | 2 | Moderate | High | High | 100.0 | Censored |
| 317 | 43 | Male | Yes | <= 5 | Single | 0-A | Ⅰ-Ⅱ | Yes | 2 | Moderate | High | 2 | Moderate | High | Low | 29.0 | Die |
| 318 | 56 | Male | Yes | <= 5 | Single | B-C | Ⅲ-Ⅳ | No | 1 | Weak | Low | 3 | Strong | High | Low | 100.0 | Censored |
| 319 | 45 | Male | Yes | <= 5 | Single | 0-A | Ⅰ-Ⅱ | Yes | 1 | Weak | Low | 1 | Weak | Low | Low | 14.0 | Die |
| 320 | 51 | Female | Yes | <= 5 | Single | 0-A | Ⅰ-Ⅱ | Yes | 2 | Moderate | High | 1 | Weak | Low | Low | 100.0 | Censored |
| 321 | 59 | Male | Yes | > 5 | Single | B-C | Ⅲ-Ⅳ | Yes | 1 | Weak | Low | 1 | Weak | Low | Low | 9.0 | Die |
| 322 | 53 | Male | Yes | > 5 | Single | B-C | Ⅰ-Ⅱ | Yes | 0 | Negative | Low | 1 | Weak | Low | Low | 14.0 | Die |
| 323 | 55 | Male | No | <= 5 | Single | 0-A | Ⅰ-Ⅱ | Yes | 2 | Moderate | High | 1 | Weak | Low | Low | 30.0 | Die |
| 324 | 51 | Male | Yes | <= 5 | Multiple | 0-A | Ⅲ-Ⅳ | No | 2 | Moderate | High | 2 | Moderate | High | Low | 100.0 | Censored |
| 325 | 77 | Male | Yes | <= 5 | Single | 0-A | Ⅰ-Ⅱ | No | 1 | Weak | Low | 1 | Weak | Low | High | 2.0 | Die |
| 326 | 28 | Male | Yes | > 5 | Single | B-C | Ⅰ-Ⅱ | Yes | 1 | Weak | Low | 2 | Moderate | High | Low | 16.0 | Die |
| 327 | 51 | Male | Yes | > 5 | Multiple | B-C | Ⅲ-Ⅳ | Yes | 2 | Moderate | High | 1 | Weak | Low | Low | 51.0 | Die |
| 328 | 51 | Male | No | <= 5 | Single | 0-A | Ⅰ-Ⅱ | Yes | 2 | Moderate | High | 1 | Weak | Low | High | 20.0 | Die |
| 329 | 48 | Male | Yes | > 5 | Multiple | B-C | Ⅲ-Ⅳ | Yes | 3 | Strong | High | 1 | Weak | Low | High | 100.0 | Censored |
| 330 | 39 | Male | Yes | <= 5 | Single | 0-A | Ⅰ-Ⅱ | Yes | 2 | Moderate | High | 1 | Weak | Low | Low | 76.0 | Censored |
| 331 | 38 | Male | Yes | > 5 | Single | B-C | Ⅲ-Ⅳ | Yes | 2 | Moderate | High | 1 | Weak | Low | High | 39.0 | Die |
| 332 | 53 | Female | Yes | <= 5 | Single | 0-A | Ⅰ-Ⅱ | No | 2 | Moderate | High | 2 | Moderate | High | Low | 98.0 | Die |
| 333 | 49 | Male | Yes | <= 5 | Single | 0-A | Ⅰ-Ⅱ | Yes | 2 | Moderate | High | 1 | Weak | Low | Low | 63.0 | Die |
| 334 | 54 | Male | Yes | > 5 | Single | B-C | Ⅰ-Ⅱ | Yes | 3 | Strong | High | 1 | Weak | Low | Low | 54.0 | Die |
| 335 | 70 | Male | No | <= 5 | Single | 0-A | Ⅰ-Ⅱ | Yes | 2 | Moderate | High | 2 | Moderate | High | Low | 95.0 | Die |
| 336 | 47 | Female | Yes | <= 5 | Single | 0-A | Ⅰ-Ⅱ | No | 2 | Moderate | High | 1 | Weak | Low | High | 100.0 | Censored |
| 337 | 48 | Male | No | <= 5 | Single | 0-A | Ⅰ-Ⅱ | Yes | 2 | Moderate | High | 2 | Moderate | High | Low | 62.0 | Die |
| 338 | 36 | Male | Yes | > 5 | Multiple | B-C | Ⅲ-Ⅳ | No | 3 | Strong | High | 2 | Moderate | High | High | 37.0 | Die |
| 339 | 52 | Male | Yes | <= 5 | Single | 0-A | Ⅰ-Ⅱ | No | 3 | Strong | High | 3 | Strong | High | High | 62.0 | Die |
| 340 | 60 | Female | Yes | <= 5 | Single | 0-A | Ⅰ-Ⅱ | Yes | 3 | Strong | High | 2 | Moderate | High | High | 22.0 | Die |
| 341 | 43 | Male | Yes | <= 5 | Single | 0-A | Ⅰ-Ⅱ | No | 1 | Weak | Low | 1 | Weak | Low | Low | 63.0 | Censored |
| 342 | 48 | Male | Yes | <= 5 | Single | 0-A | Ⅰ-Ⅱ | No | 3 | Strong | High | 2 | Moderate | High | Low | 99.0 | Censored |
| 343 | 57 | Male | Yes | > 5 | Single | B-C | Ⅲ-Ⅳ | No | 2 | Moderate | High | 1 | Weak | Low | High | 99.0 | Censored |
| 344 | 54 | Male | No | <= 5 | Single | B-C | Ⅲ-Ⅳ | No | 1 | Weak | Low | 2 | Moderate | High | Low | 39.0 | Censored |
| 345 | 54 | Male | No | > 5 | Single | B-C | Ⅰ-Ⅱ | Yes | 2 | Moderate | High | 2 | Moderate | High | High | 15.0 | Die |
| 346 | 53 | Male | Yes | <= 5 | Multiple | B-C | Ⅲ-Ⅳ | Yes | 2 | Moderate | High | 2 | Moderate | High | High | 66.0 | Die |
| 347 | 34 | Male | Yes | <= 5 | Single | B-C | Ⅲ-Ⅳ | No | 2 | Moderate | High | 1 | Weak | Low | Low | 99.0 | Censored |
| 348 | 55 | Male | Yes | > 5 | Single | B-C | Ⅲ-Ⅳ | Yes | 3 | Strong | High | 2 | Moderate | High | Low | 61.0 | Die |
| 349 | 56 | Male | Yes | > 5 | Single | B-C | Ⅰ-Ⅱ | No | 2 | Moderate | High | 2 | Moderate | High | High | 101.0 | Censored |
| 350 | 62 | Male | Yes | <= 5 | Single | 0-A | Ⅰ-Ⅱ | Yes | 2 | Moderate | High | 2 | Moderate | High | Low | 99.0 | Censored |
| 351 | 72 | Male | Yes | <= 5 | Single | 0-A | Ⅰ-Ⅱ | Yes | 2 | Moderate | High | 2 | Moderate | High | Low | 37.0 | Die |
| 352 | 53 | Male | No | <= 5 | Single | 0-A | Ⅰ-Ⅱ | Yes | 2 | Moderate | High | 1 | Weak | Low | Low | 24.0 | Die |
| 353 | 58 | Male | No | <= 5 | Single | 0-A | Ⅰ-Ⅱ | Yes | 2 | Moderate | High | 1 | Weak | Low | Low | 99.0 | Die |
| 354 | 45 | Male | Yes | > 5 | Multiple | B-C | Ⅲ-Ⅳ | No | 2 | Moderate | High | 3 | Strong | High | Low | 99.0 | Censored |
| 355 | 67 | Male | Yes | <= 5 | Single | B-C | Ⅲ-Ⅳ | Yes | 2 | Moderate | High | 0 | Negative | Low | Low | 89.0 | Die |
| 356 | 70 | Female | Yes | > 5 | Single | B-C | Ⅲ-Ⅳ | Yes | 2 | Moderate | High | 2 | Moderate | High | Low | 25.0 | Die |
| 357 | 18 | Female | Yes | > 5 | Single | B-C | Ⅰ-Ⅱ | Yes | 2 | Moderate | High | 2 | Moderate | High | High | 43.0 | Die |
| 358 | 56 | Male | Yes | <= 5 | Single | 0-A | Ⅰ-Ⅱ | Yes | 3 | Strong | High | 2 | Moderate | High | High | 99.0 | Censored |
| 359 | 46 | Male | Yes | <= 5 | Single | 0-A | Ⅰ-Ⅱ | Yes | 2 | Moderate | High | 2 | Moderate | High | High | 99.0 | Censored |
| 360 | 45 | Male | Yes | <= 5 | Single | 0-A | Ⅰ-Ⅱ | Yes | 2 | Moderate | High | 1 | Weak | Low | High | 52.0 | Die |
| 361 | 55 | Male | Yes | <= 5 | Single | B-C | Ⅲ-Ⅳ | No | 2 | Moderate | High | 2 | Moderate | High | High | 75.0 | Censored |
| 362 | 73 | Female | Yes | > 5 | Single | B-C | Ⅰ-Ⅱ | No | 3 | Strong | High | 2 | Moderate | High | Low | 101.0 | Censored |
| 363 | 37 | Female | Yes | > 5 | Single | B-C | Ⅲ-Ⅳ | No | 2 | Moderate | High | 2 | Moderate | High | High | 99.0 | Censored |
| 364 | 52 | Female | Yes | > 5 | Single | B-C | Ⅲ-Ⅳ | No | 1 | Weak | Low | 1 | Weak | Low | High | 65.0 | Die |
| 365 | 65 | Male | Yes | <= 5 | Single | B-C | Ⅲ-Ⅳ | Yes | 2 | Moderate | High | 2 | Moderate | High | High | 52.0 | Censored |
| 366 | 56 | Male | Yes | <= 5 | Single | 0-A | Ⅰ-Ⅱ | Yes | 1 | Weak | Low | 2 | Moderate | High | High | 88.0 | Die |
| 367 | 53 | Male | Yes | <= 5 | Single | B-C | Ⅲ-Ⅳ | No | 2 | Moderate | High | 2 | Moderate | High | Low | 91.0 | Die |
| 368 | 35 | Female | No | <= 5 | Single | 0-A | Ⅰ-Ⅱ | No | 2 | Moderate | High | 2 | Moderate | High | High | 99.0 | Censored |
| 369 | 67 | Female | Yes | <= 5 | Single | 0-A | Ⅰ-Ⅱ | No | 1 | Weak | Low | 2 | Moderate | High | Low | 99.0 | Censored |
| 370 | 64 | Male | Yes | <= 5 | Multiple | B-C | Ⅲ-Ⅳ | Yes | 1 | Weak | Low | 1 | Weak | Low | Low | 41.0 | Die |
| 371 | 37 | Male | Yes | > 5 | Multiple | B-C | Ⅲ-Ⅳ | Yes | 2 | Moderate | High | 2 | Moderate | High | Low | 45.0 | Die |
| 372 | 56 | Male | No | <= 5 | Multiple | B-C | Ⅲ-Ⅳ | No | 1 | Weak | Low | 1 | Weak | Low | Low | 99.0 | Censored |
| 373 | 52 | Male | Yes | > 5 | Multiple | B-C | Ⅲ-Ⅳ | Yes | 1 | Weak | Low | 0 | Negative | Low | High | 12.0 | Die |
| 374 | 67 | Male | Yes | <= 5 | Multiple | 0-A | Ⅲ-Ⅳ | Yes | 1 | Weak | Low | 1 | Weak | Low | Low | 12.0 | Die |
| 375 | 31 | Male | Yes | <= 5 | Single | 0-A | Ⅰ-Ⅱ | No | 1 | Weak | Low | 1 | Weak | Low | Low | 99.0 | Censored |
| 376 | 49 | Male | Yes | <= 5 | Single | 0-A | Ⅰ-Ⅱ | No | 1 | Weak | Low | 1 | Weak | Low | High | 99.0 | Censored |
| 377 | 45 | Male | Yes | <= 5 | Multiple | B-C | Ⅲ-Ⅳ | No | 2 | Moderate | High | 2 | Moderate | High | High | 99.0 | Censored |
| 378 | 39 | Male | Yes | <= 5 | Single | B-C | Ⅲ-Ⅳ | Yes | 2 | Moderate | High | 2 | Moderate | High | Low | 15.0 | Die |
| 379 | 69 | Male | Yes | <= 5 | Single | 0-A | Ⅰ-Ⅱ | No | 1 | Weak | Low | 2 | Moderate | High | Low | 99.0 | Censored |
| 380 | 41 | Male | No | > 5 | Single | B-C | Ⅰ-Ⅱ | Yes | 1 | Weak | Low | 1 | Weak | Low | Low | 13.0 | Die |
| 381 | 52 | Male | Yes | <= 5 | Single | 0-A | Ⅰ-Ⅱ | No | 2 | Moderate | High | 2 | Moderate | High | Low | 99.0 | Censored |
| 382 | 34 | Female | Yes | <= 5 | Single | 0-A | Ⅰ-Ⅱ | No | 1 | Weak | Low | 1 | Weak | Low | Low | 99.0 | Censored |
