# Supplementary material for: Differential expression pattern of CC chemokine receptor 7 guides precision treatment of hepatocellular carcinoma
Source: Signal Transduct Target Ther. 2025 Jul 21;10:229. doi: 10.1038/s41392-025-02308-6 (PMC12277428; doi:10.1038/s41392-025-02308-6)
Supplement: Supplementary file 4 — Supplementary materials for original images of western blot [file 41392_2025_2308_MOESM4_ESM.docx]

**Supplemental materials for**

**Differential expression pattern of CC chemokine receptor 7 guides precision treatment of hepatocellular carcinoma**

**Running title:** The Effect of CCL21/CCR7 axis in Tumor Microenvironment

Jie Qin^1†^, Qianyi Gong^1†^, Cheng Zhou^2†^, Jietian Xu^1,3†^, Yifei Cheng^2†^, Weiyue Xu^1^, Di Zhu^1^, Yiming Liu^1^, Yuye Zhang^1^, Yanru Wang^1^, Lingling Gao^1^, Lanfang Li^1^, Wulei Hou^1^, Qian Li^1^, Binbin Liu^2^, Yazhen Zhu^4^, Zuoyun Wang^1^, Jieyi Shi^2*^, Shuangjian Qiu^2*^, Chunmin Liang^1,2,3*^

* Correspondence to: cmliang@fudan.edu.cn (Chunmin Liang);

qiu.shuangjian@zs-hospital.sh.cn (Shuangjian Qiu);

shi.jieyi@zs-hospital.sh.cn (Jieyi Shi);

† These authors have contributed equally to this work and share first authorship

**This file includes:** Original images of western blot

**
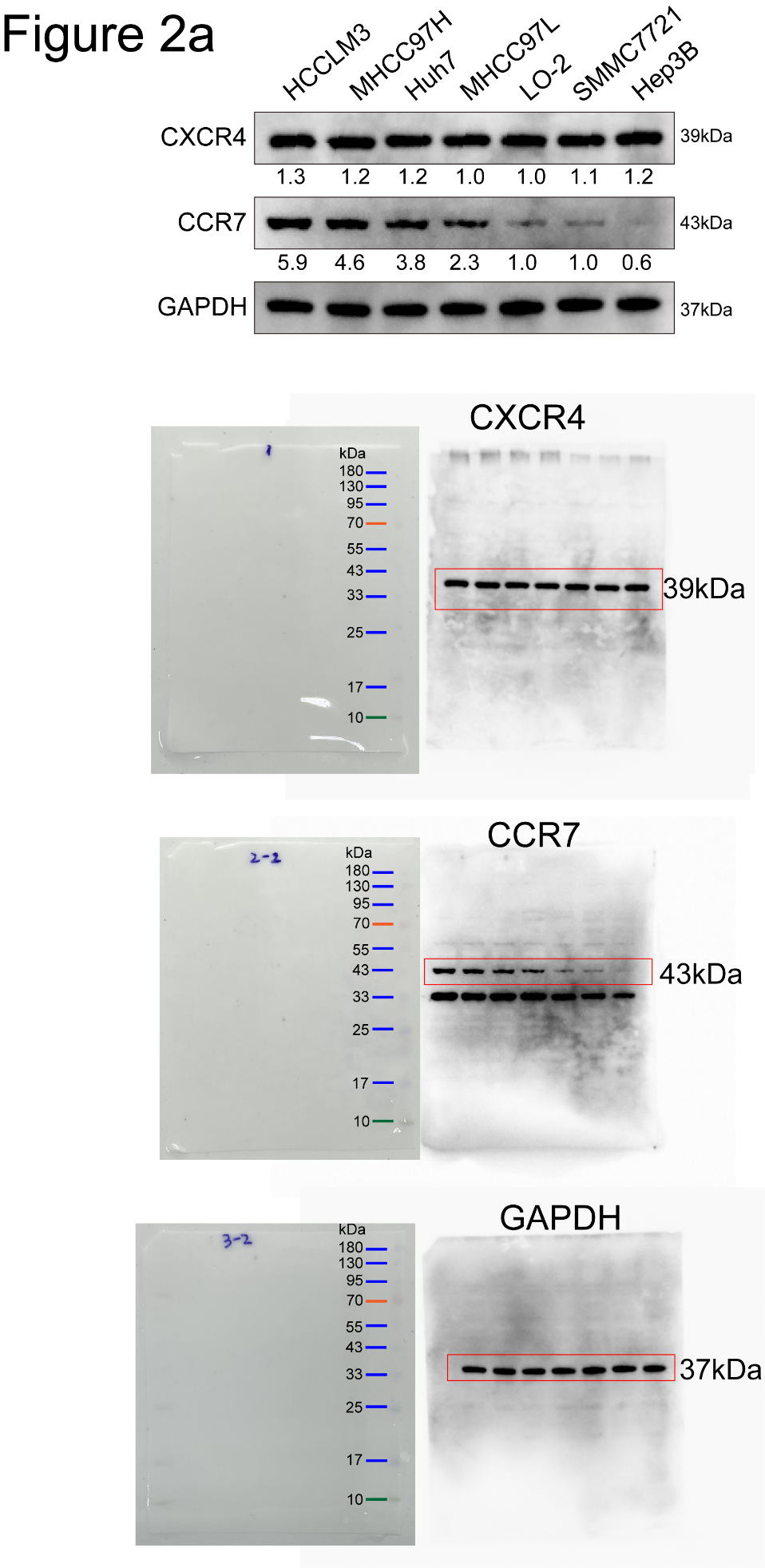
**

**
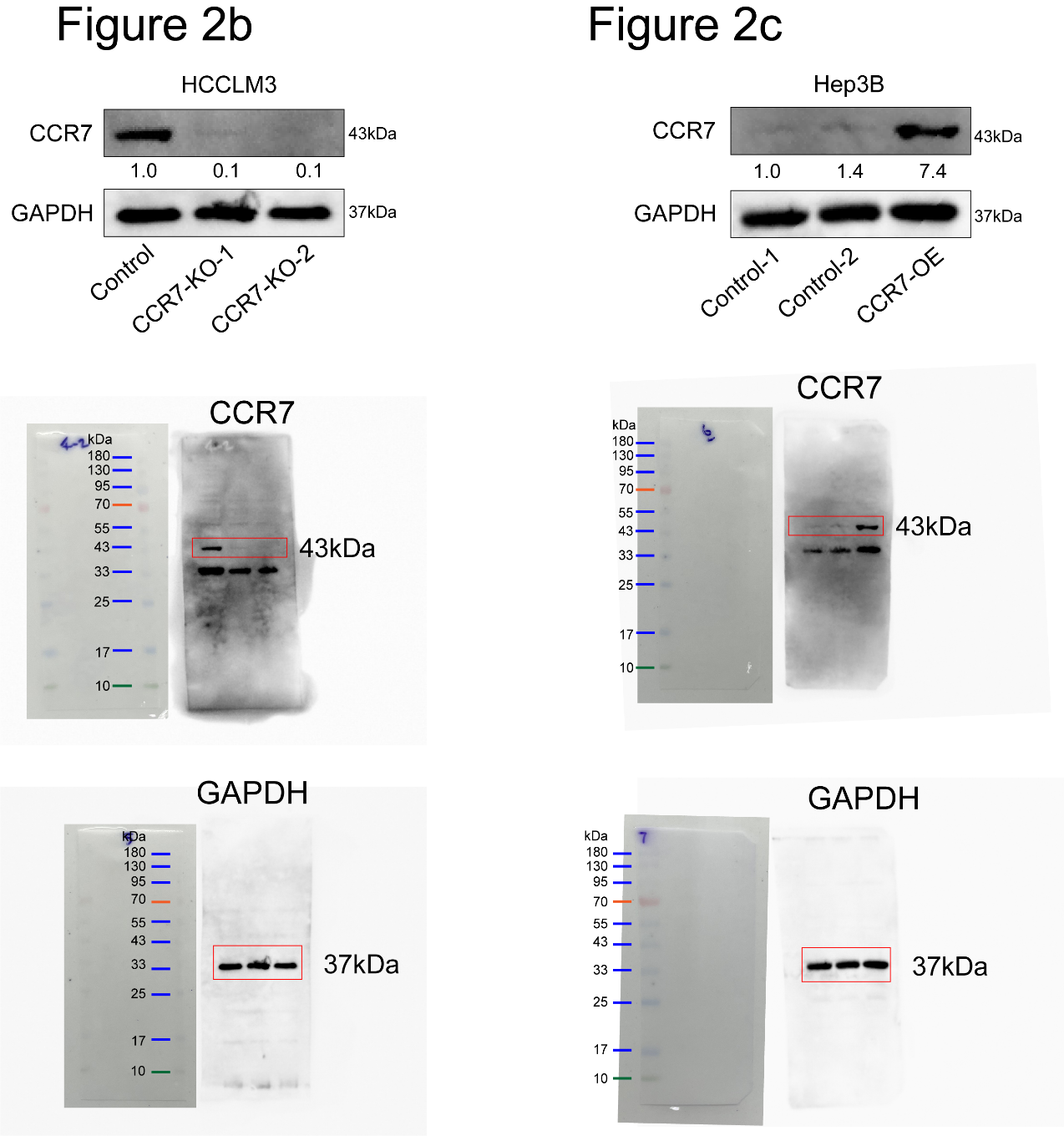
**

**
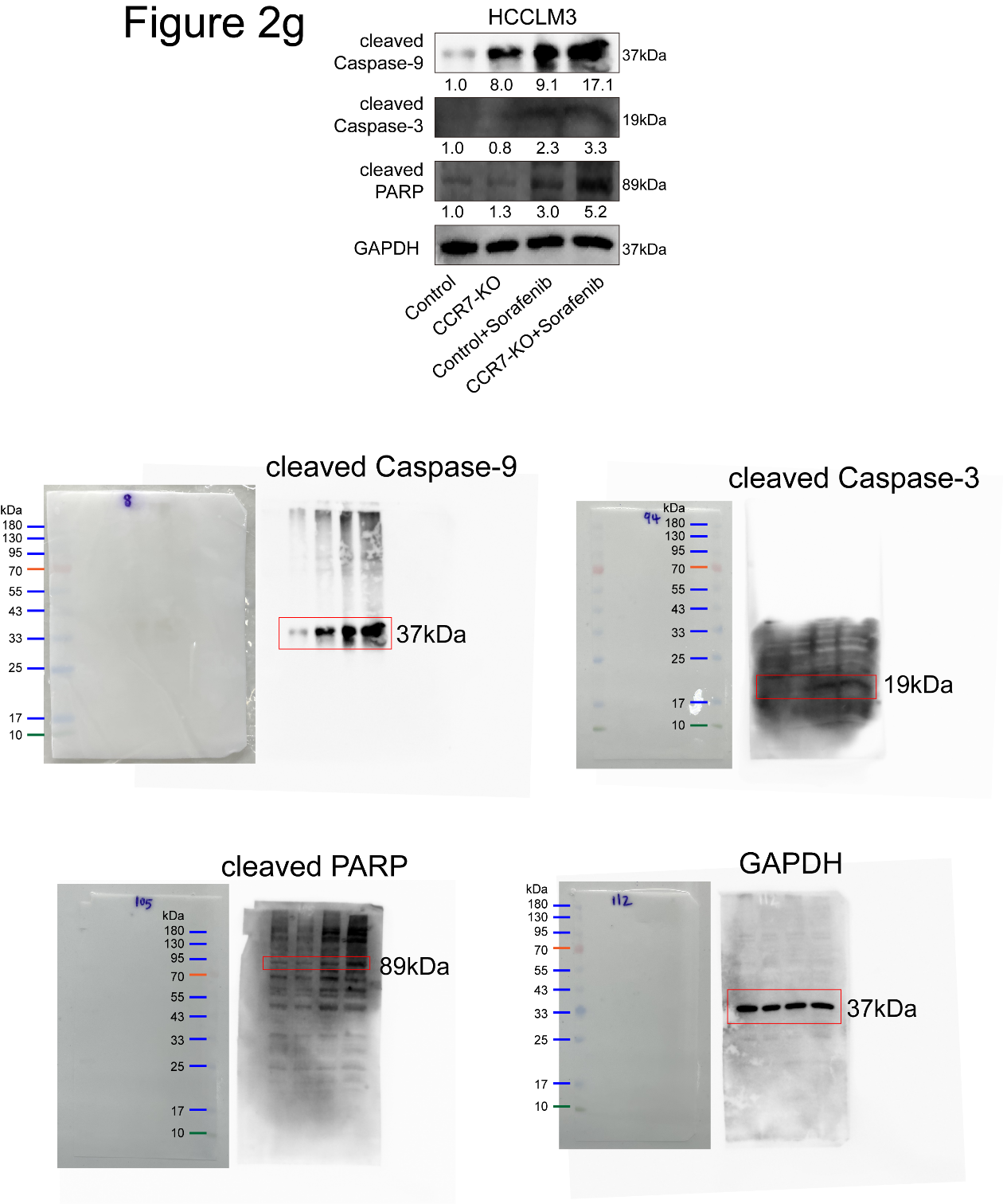
**

**
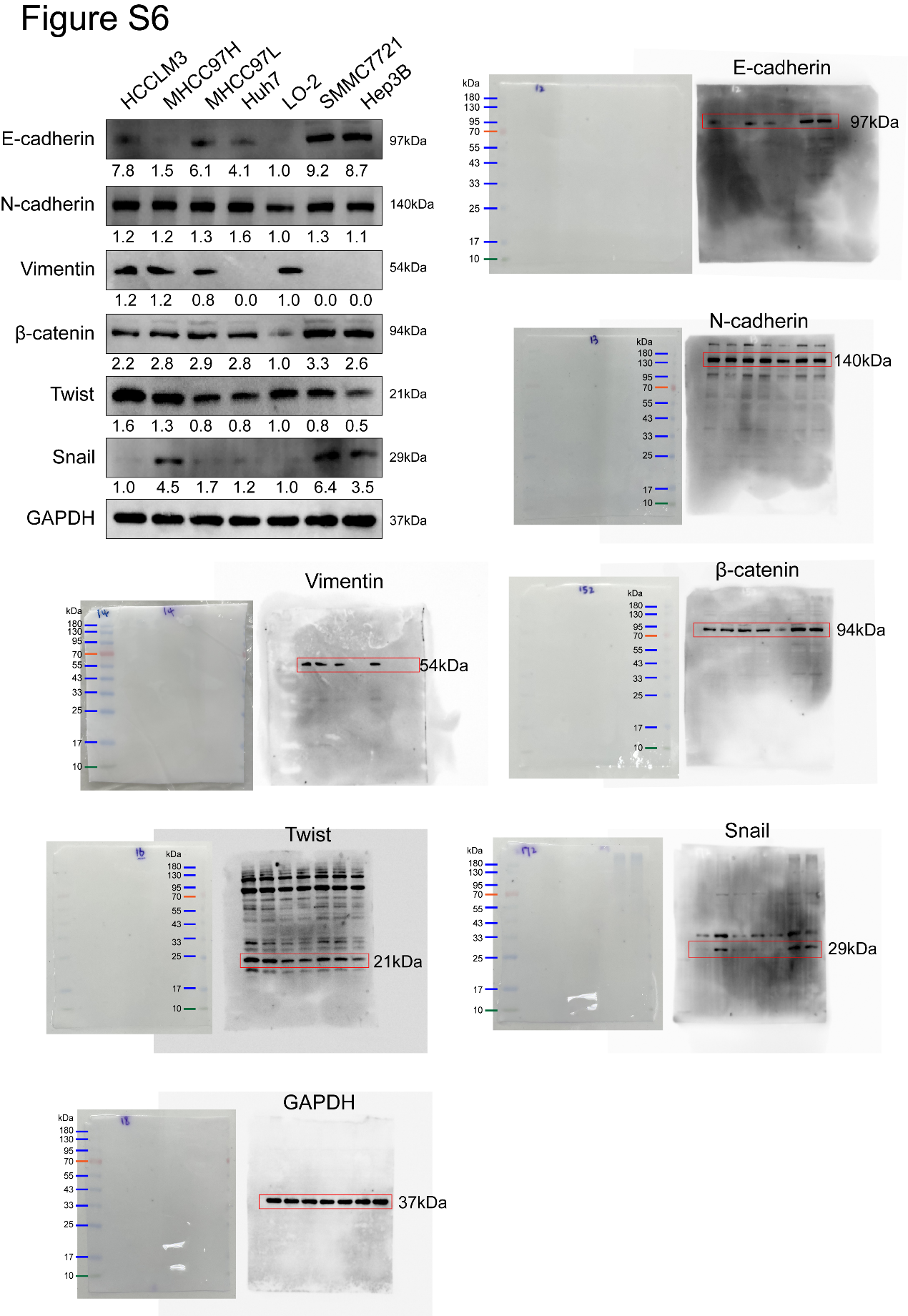
**

**
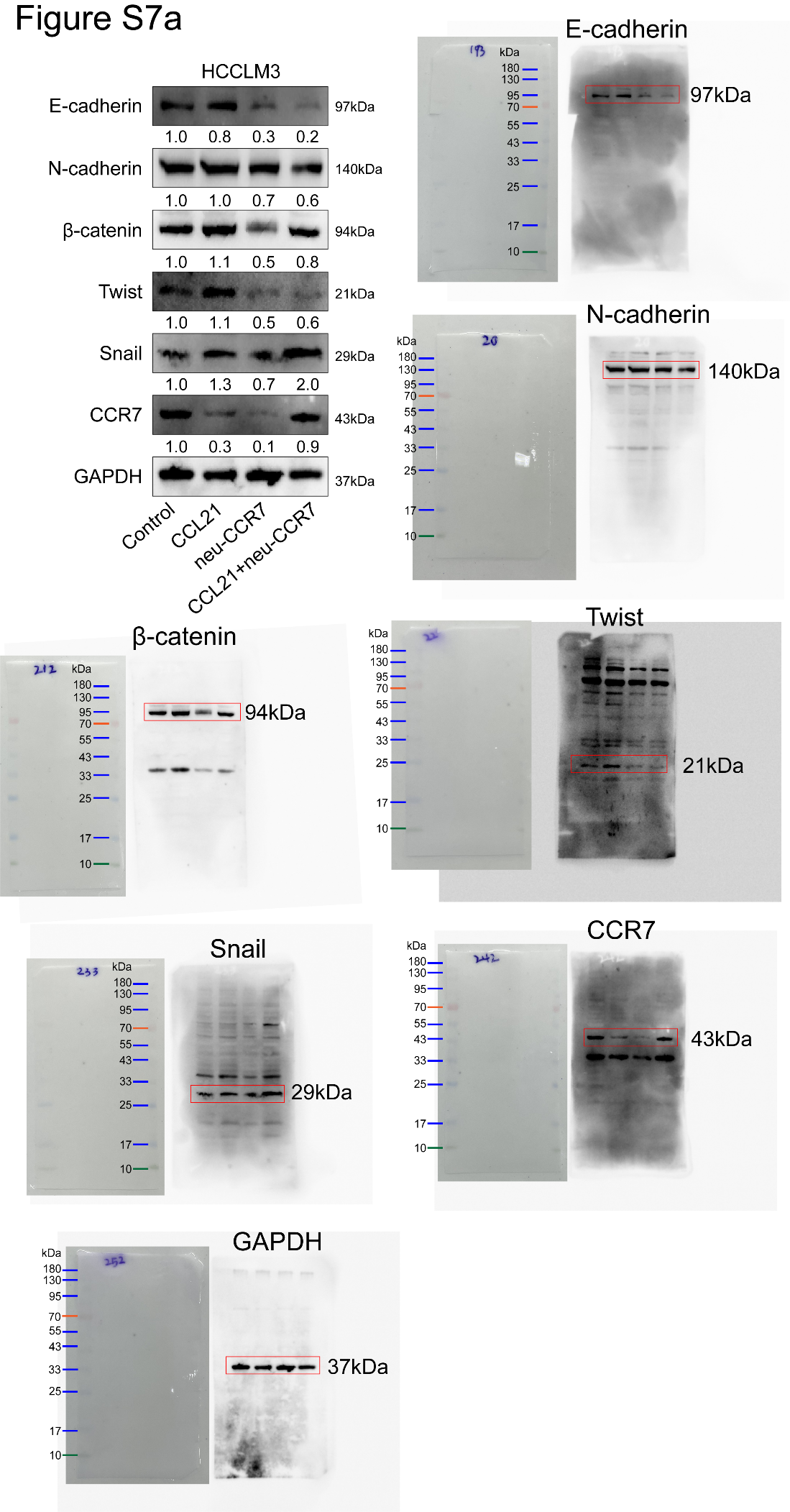
**

**
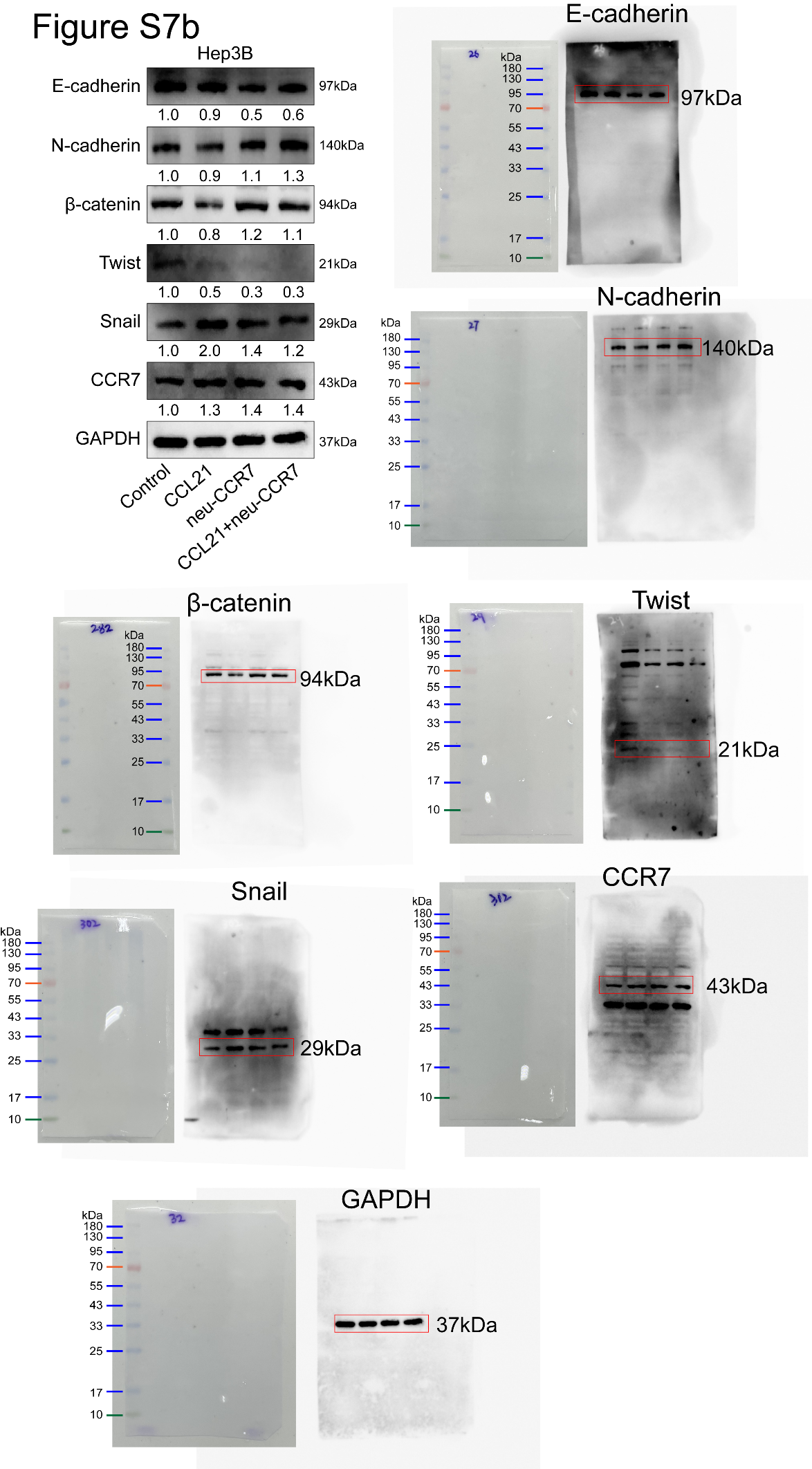
**

**
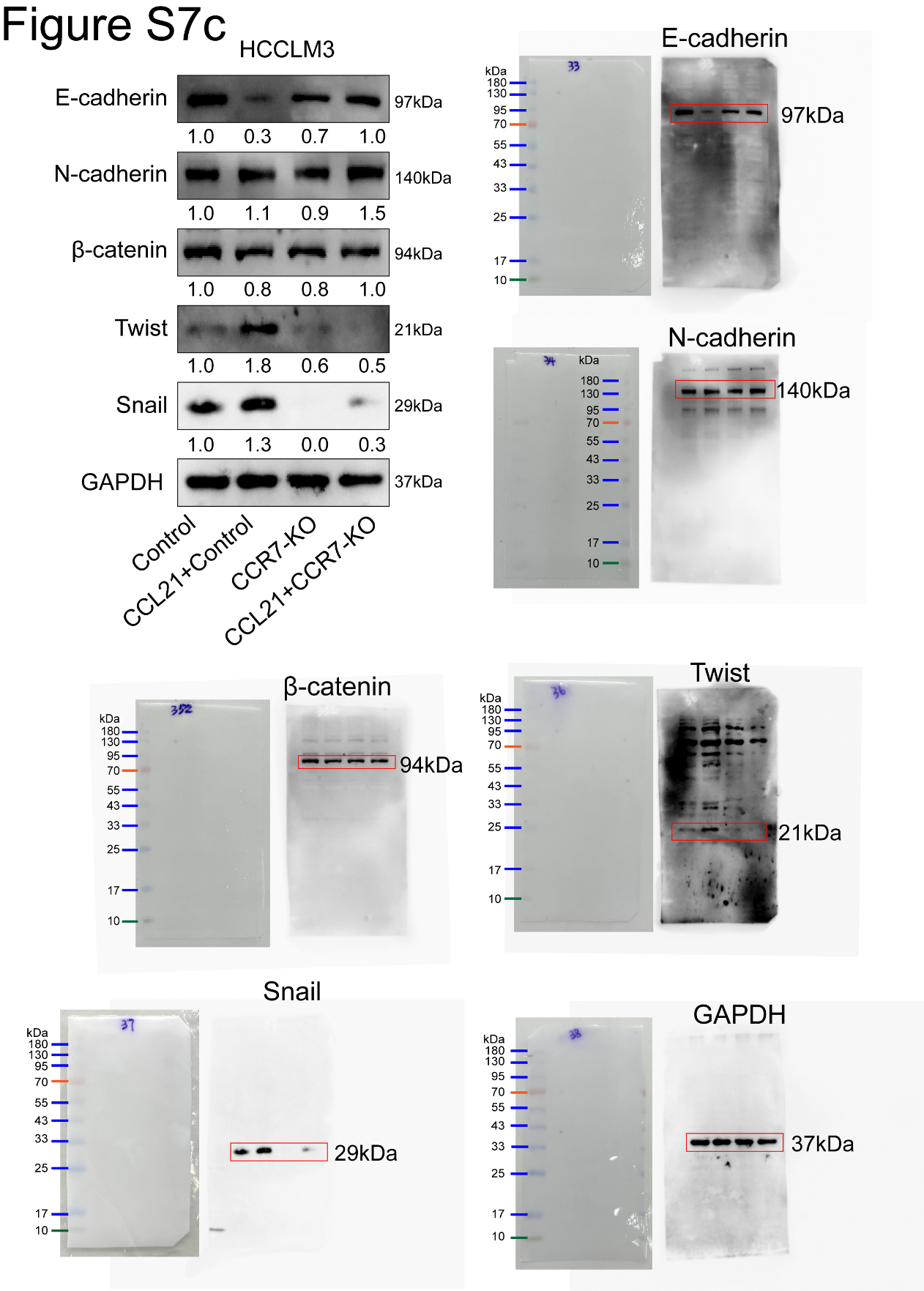
**

**
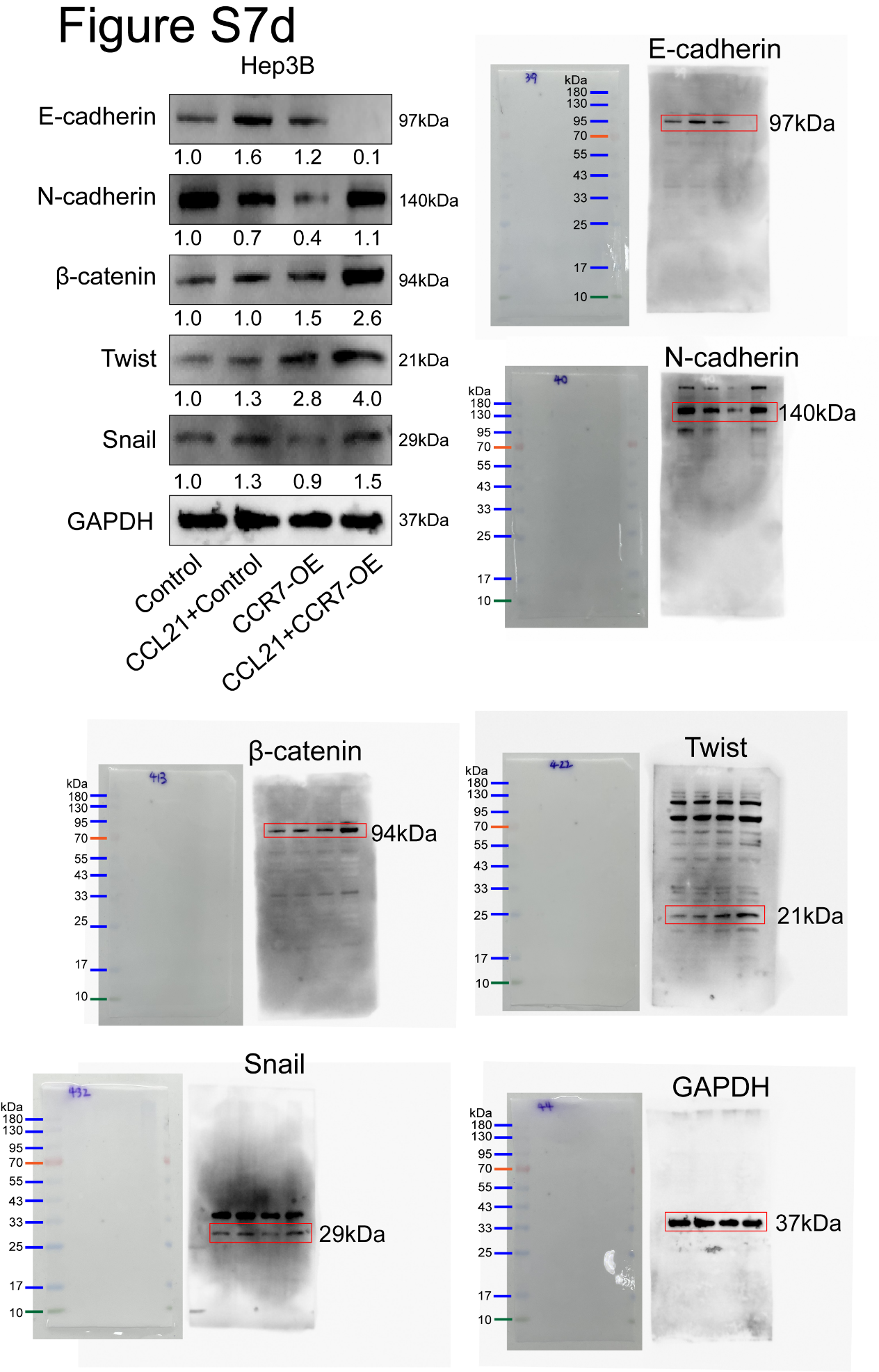
**

**
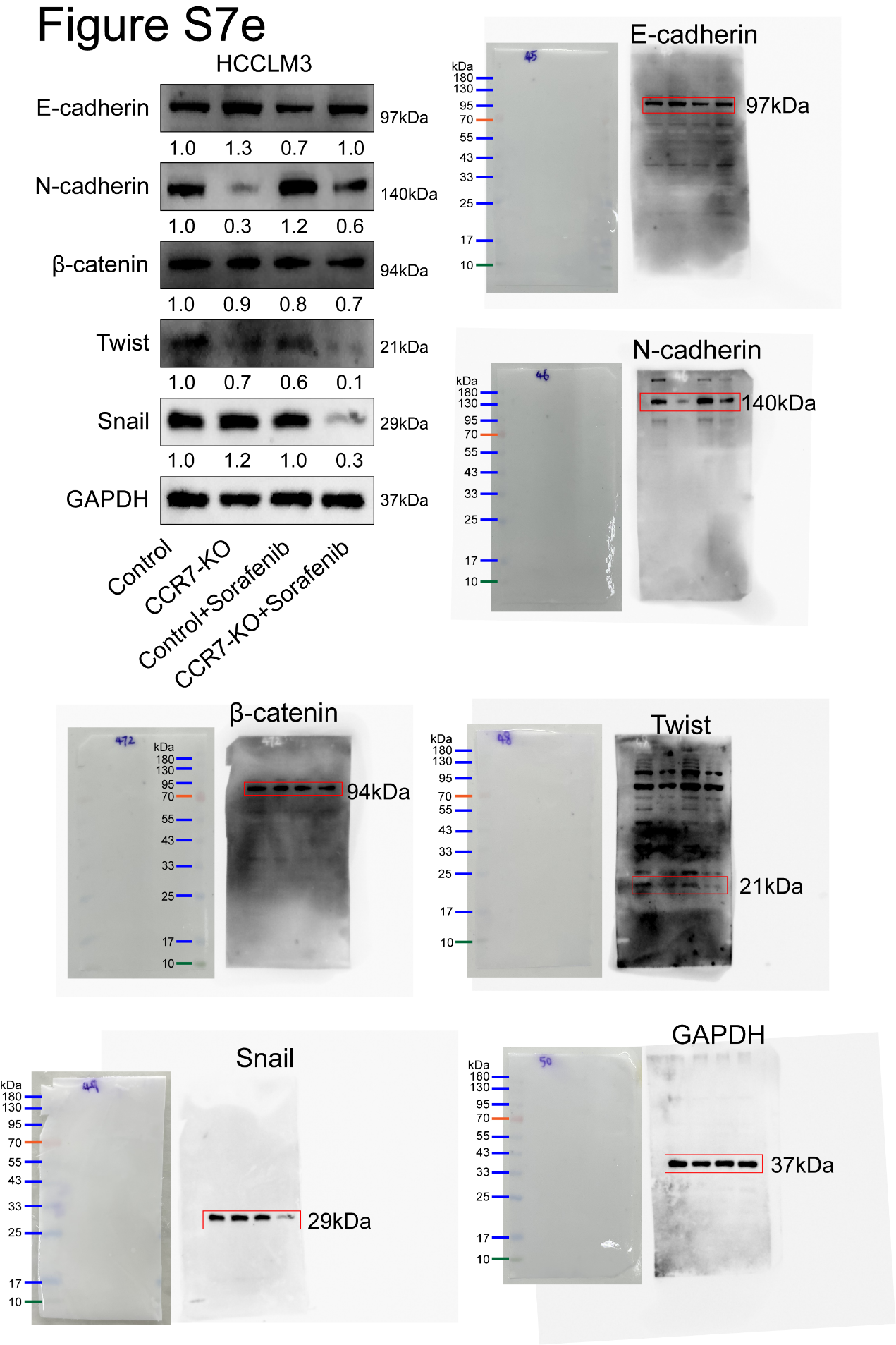
**

**
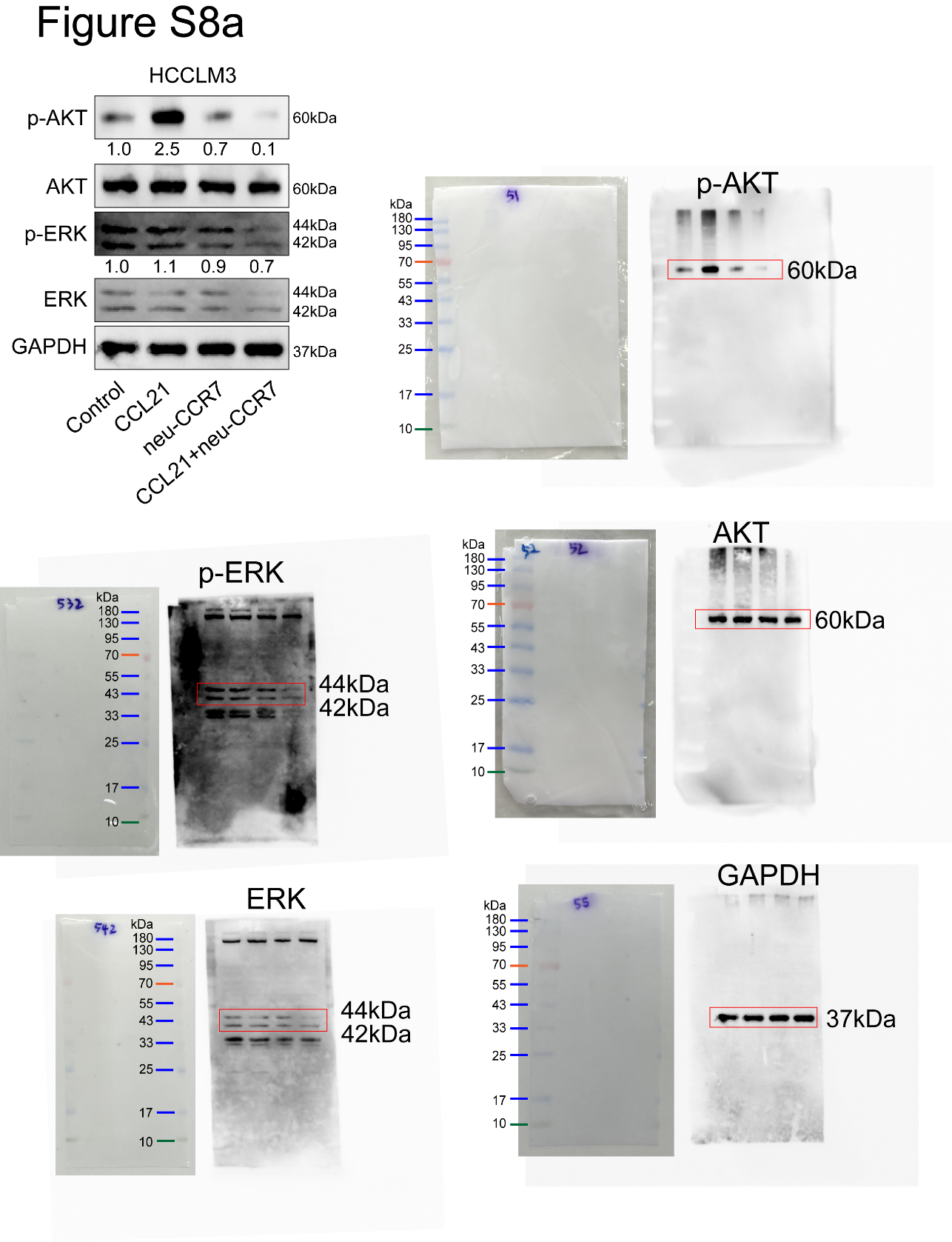
**

**
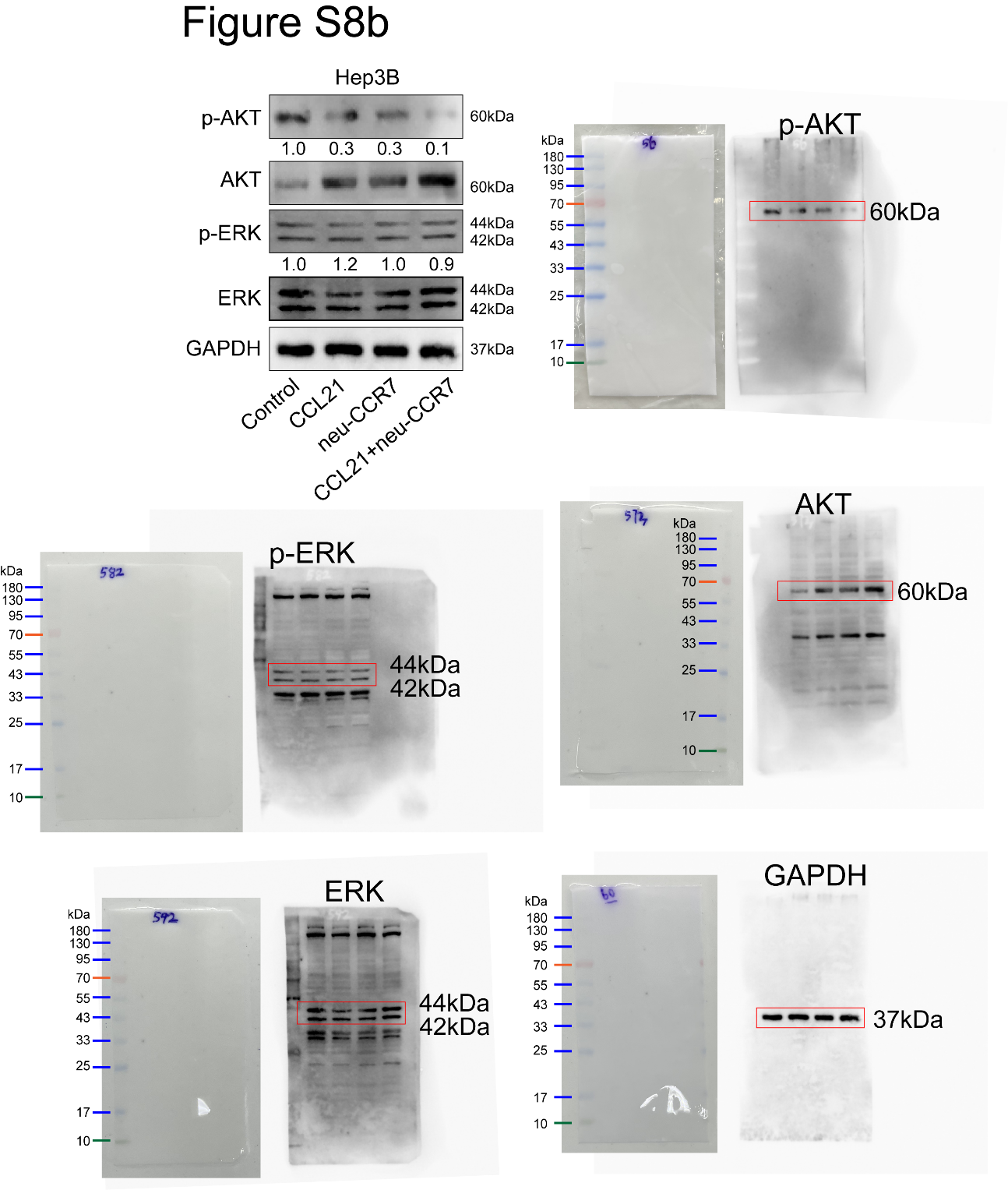
**

**
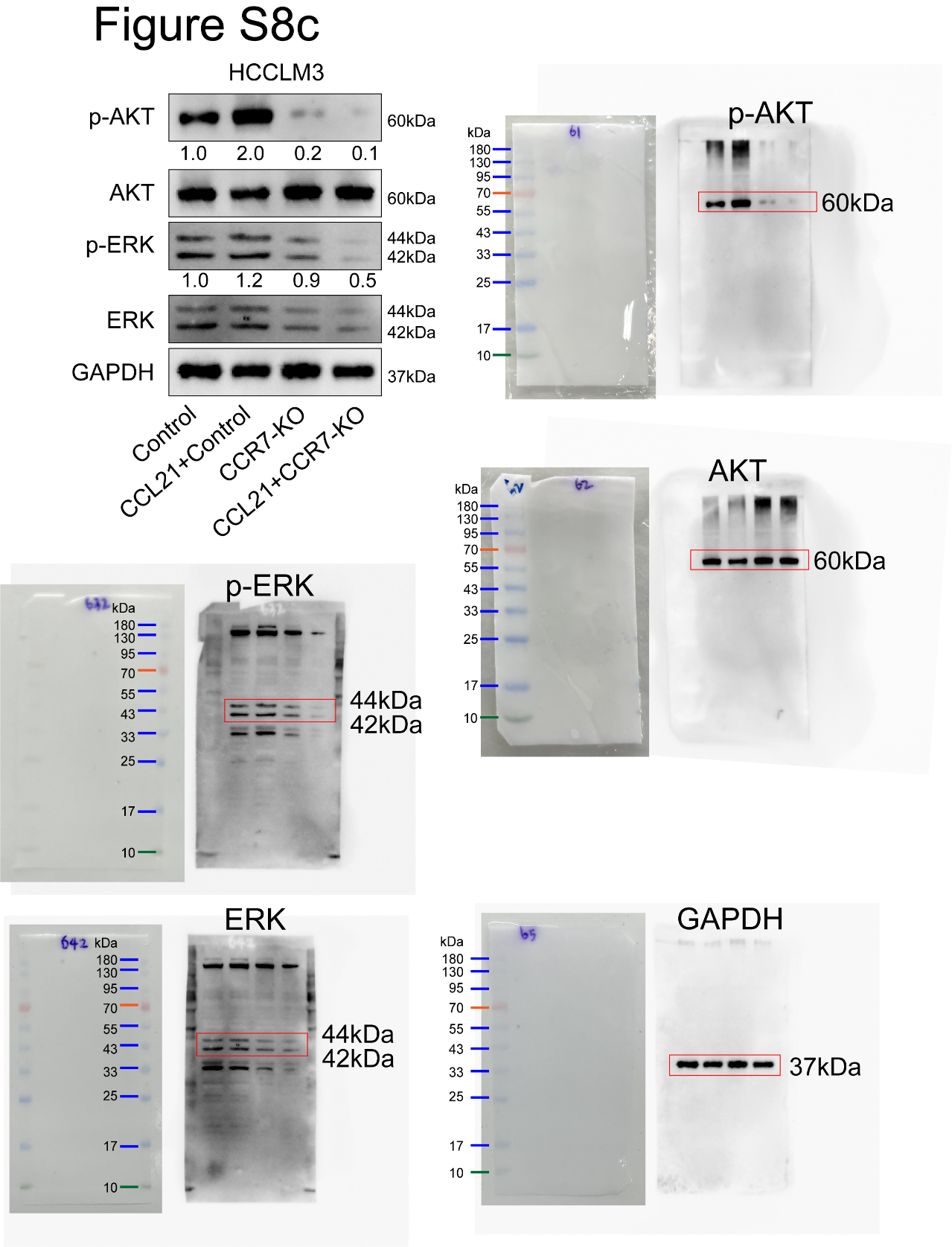
**

**
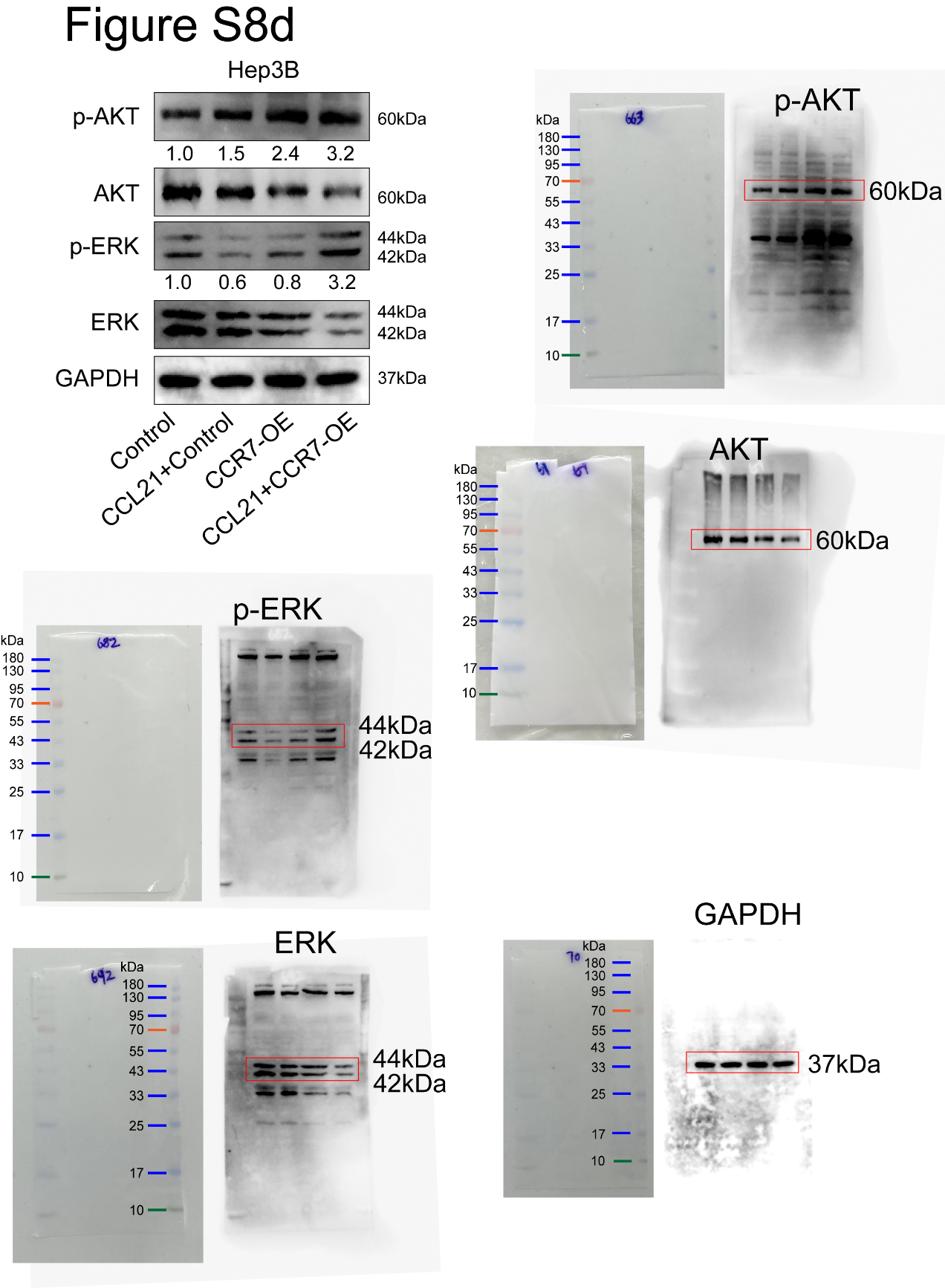
**

**
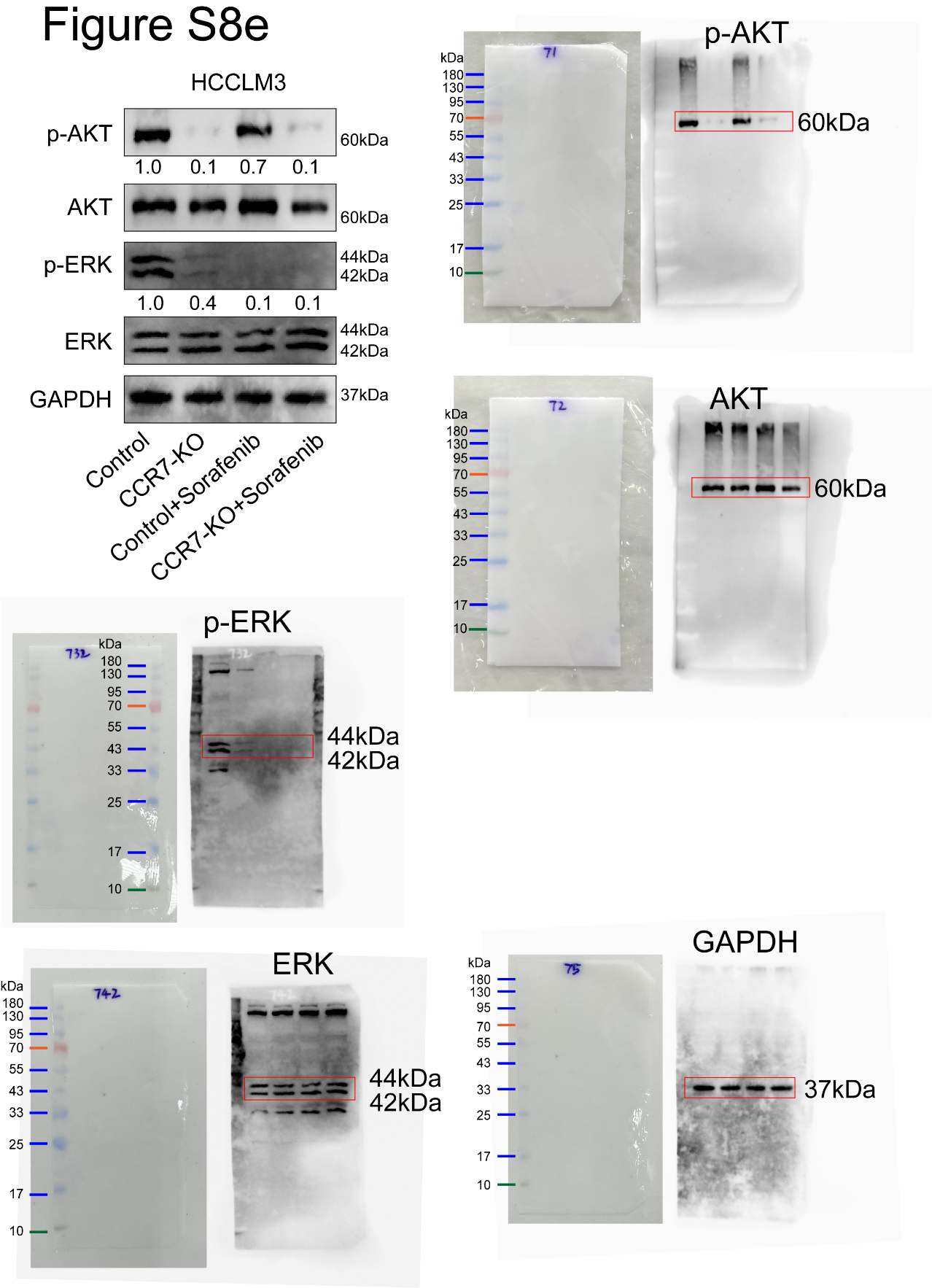
**
