# Supplementary material for: Differential expression pattern of CC chemokine receptor 7 guides precision treatment of hepatocellular carcinoma
Source: Signal Transduct Target Ther. 2025 Jul 21;10:229. doi: 10.1038/s41392-025-02308-6 (PMC12277428; doi:10.1038/s41392-025-02308-6)
Supplement: Supplementary file 5 — 06-16-Authorship form with signatures [file 41392_2025_2308_MOESM5_ESM.pdf]

SPRINGER NATURE

Change of authorship request form - Journals (pre-acceptance)

Important information. Please read.

- This form should be used by authors to request any change in authorship (adding/deleting authors) including changes in corresponding authors. This form should not be used for name changes. Please fully complete all sections. Use black ink and block capitals and provide each author's full name with the given name first followed by the family name.

- By signing this declaration, all authors guarantee that the order of the authors are in accordance with their scientific contribution, if applicable as different conventions apply per discipline, and that only authors have been added who made a meaningful contribution to the work.

- Please note, in author collaborations where there is formal agreement for representing the collaboration, it is sufficient for the representative or legal guarantor (usually the corresponding author) to complete and sign the Authorship Change Form on behalf of all authors, **next to the added/removed author(s)**. **(Complete Section 3, followed by Section 6.)**
  In author collaborations where there is no formal agreement for representing the collaboration and **there are more than 10 authors**, one may sign for all, provided the signer appends correspondence that attests that each of the authors have agreed to the change **and the added/removed authors sign the form**. **(Complete Section 3, followed by Section 6.)**

- Please note, we cannot investigate or mediate any authorship disputes. If you are unable to obtain agreement from all authors (including those who you wish to be removed) you must refer the matter to your institution(s) for investigation. Please inform us if you need to do this.

- If you are not able to return a fully completed form within **30 days** of the date that it was sent to the author requesting the change, we may have to withdraw your manuscript. We cannot publish manuscripts where authorship has not been agreed by all authors (including those who have been removed).

- Incomplete forms will be rejected.

- Please return/upload this form, fully completed, to the Journals Editorial Office. The Journal and/or Publisher will consider the information you have provided to decide whether to approve the proposed change in authorship. We may decide to contact your institution for more information or undertake a further investigation, if appropriate, before making a final decision.

SPRINGER NATURE

Change of authorship request form - Journals (pre-acceptance)

Section 1: Please provide the current title of manuscript

Manuscript ID no.: SIGTRANS-16557R1

Title: Differential expression pattern of CC chemokine receptor 7 guides precision treatment of hepatocellular carcinoma

Section 2: Please provide the previous authorship, in the order shown on the manuscript before the changes were introduced. Please indicate the corresponding author by adding (CA) behind the name.

	First name(s)	Family name	ORCID or SCOPUS id, if available
1st author	JIE	QIN	
2nd author	QIANYI	GONG	
3rd author	CHENG	ZHOU	
4th author	JIETIAN	XU	
5th author	WEIYUE	XU	
6th author	YIFEI	CHENG	
7th author	DI	ZHU	
8th author	YIMING	LIU	
9th author	YUYE	ZHANG	
10th author	YANRU	WANG	

Please use an additional sheet if there are more than 10 authors.

SPRINGER NATURE Springer Nature is one of the world's leading global research, educational and professional publishers, created in May 2015 through the combination of Nature Publishing Group, Palgrave Macmillan, Macmillan Education and Springer Science+Business Media.

Change of authorship request form - Journals (pre-acceptance)

Section 2: Please provide the previous authorship, in the order shown on the manuscript before the changes were introduced. Please indicate the corresponding author by adding (CA) behind the name.

	First name(s)	Family name	ORCID or SCOPUS id, if available
11th author	LINGLING	GAO	
12th author	LANFANG	LI	
13th author	WULEI	HOU	
14th author	QIAN	LI	
15th author	BINBIN	LIU	
16th author	YAZHEN	ZHU	
17th author	ZUOYUN	WANG (CA)	
18th author	JIEYI	SHI (CA)	
19th author	SHUANGJIAN	QIU (CA)	
20th author	CHUNMIN	LIANG (CA)	https://orcid.org/0000-0002-6129-9902

Please use an additional sheet if there are more than 10 authors.

SPRINGER NATURE Springer Nature is one of the world's leading global research, educational and professional publishers, created in May 2015 through the combination of Nature Publishing Group, Palgrave Macmillan, Macmillan Education and Springer Science+Business Media.

SPRINGER NATURE

Change of authorship request form - Journals (pre-acceptance)

Section 3: Please provide a justification for change. Please use this section to explain your reasons for changing the authorship of your manuscript, e.g. what necessitated the change in authorship? Please refer to the (journal) policy pages for more information about authorship. Please explain why omitted authors were not originally included and/or why authors were removed on the submitted manuscript.

(1) According to the journal's requirements, no more than 3 corresponding authors are allowed. The corresponding authors have been adjusted based on the level of contribution. The revised 3 corresponding authors are listed in the revised MS.

(2) In addition, YIFEI CHENG's order has been adjusted to as a co-first author (5th position). She has made significant contributions during the revision period, particularly in analyzing the clinical patient database, which provided stronger support for the results.

Section 4: Proposed new authorship. Please provide your new authorship list in the order you would like it to appear on the manuscript. Please indicate the corresponding author by adding (CA) behind the name. <u>If the Corresponding Author has changed, please indicate the reason under section 3</u>.

	First name(s)	Family name (this name will appear in full on the final publication and will be searchable in various abstract and indexing databases)	Affiliated institute	E-mail address
1st author	JIE	QIN	School of Basic Medical Sciences, Fudan University	shhqj@shmu.edu.cn
2nd author	QIANYI	GONG	School of Basic Medical Sciences, Fudan University	gongqianyi95@163.com
3rd author	CHENG	ZHOU	Zhongshan Hospital, Fudan University	zhoucheng16@fudan.edu.cn
4th author	JIETIAN	XU	School of Basic Medical Sciences, Fudan University	354865653@qq.com
5th author	YIFEI	CHENG	Zhongshan Hospital, Fudan University	chengyf21@m.fudan.edu.cn
6th author	WEIYUE	XU	School of Basic Medical Sciences, Fudan University	378702189@qq.com
7th author	DI	ZHU	School of Basic Medical Sciences, Fudan University	judy19730@163.com
8th author	LIMING	LIU	School of Basic Medical Sciences, Fudan University	17301010006@fudan.edu.cn
9th author	YUYE	ZHANG	School of Basic Medical Sciences, Fudan University	yyzhang2@163.com
10th author	YANRU	WANG	School of Basic Medical Sciences, Fudan University	Yera176@163.com

Please use an additional sheet if there are more than 10 authors.

SPRINGER NATURE Springer Nature is one of the world's leading global research, educational and professional publishers, created in May 2015 through the combination of Nature Publishing Group, Palgrave Macmillan, Macmillan Education and Springer Science+Business Media.

SPRINGER NATURE

Change of authorship request form - Journals (pre-acceptance)

Section 4: Proposed new authorship. Please provide your new authorship list in the order you would like it to appear on the manuscript. Please indicate the corresponding author by adding (CA) behind the name. If the Corresponding Author has changed, please indicate the reason under section 3.

	First name(s)	Family name (this name will appear in full on the final publication and will be searchable in various abstract and indexing databases)	Affiliated institute	E-mail address
11th author	LINGLING	GAO	School of Basic Medical Sciences, Fudan University	1642079824@qq.com
12th author	LANFANG	LI	School of Basic Medical Sciences, Fudan University	lf8858420452@163.com
13th author	WULEI	HOU	School of Basic Medical Sciences, Fudan University	houwl23@m.fudan.edu.cn
14th author	QIAN	LI	School of Basic Medical Sciences, Fudan University	qianli_fd@fudan.edu.cn
15th author	BINBIN	LIU	Zhongshan Hospital, Fudan University	liu.binbin@zs-hospital.sh.cn
16th author	YAZHEN	ZHU	David Geffen School of Medicine at UCLA	yazhenzhu@mednet.ucla.edu
17th author	ZUOYUN	WANG	School of Basic Medical Sciences, Fudan University	wangzuoyun@fudan.edu.cn
18th author	JIEYI	SHI (CA)	Zhongshan Hospital, Fudan University	shi.jieyi@zs-hospital.sh.cn
19th author	SHUANGJIAN	QIU (CA)	Zhongshan Hospital, Fudan University	qiu.shuangjian@zs-hospital.sh.cn
20th author	CHUNMIN	LIANG (CA)	School of Basic Medical Sciences, Fudan University	cmliang@fudan.edu.cn

Please use an additional sheet if there are more than 10 authors.

SPRINGER NATURE Springer Nature is one of the world's leading global research, educational and professional publishers, created in May 2015 through the combination of Nature Publishing Group, Palgrave Macmillan, Macmillan Education and Springer Science+Business Media.

SPRINGER NATURE

Change of authorship request form - Journals (pre-acceptance)

Section 5: Author contribution, Acknowledgement and Disclosures. Please use this section to provide a new disclosure statement and, if appropriate, acknowledge any contributors who have been removed as authors and ensure you state what contribution any new authors made (if applicable per the journal or book (series) policy). Please ensure these are updated in your manuscript - after approval of the change(s) - as our production department will not transfer the information in this form to your manuscript.

New acknowledgements:

Not applicable

New Disclosures (financial and non-financial interests, funding):

Not applicable

New Author Contributions statement (if applicable per the journal policy):

Not applicable

State 'Not applicable' if there are no new authors.

SPRINGER NATURE Springer Nature is one of the world's leading global research, educational and professional publishers, created in May 2015 through the combination of Nature Publishing Group, Palgrave Macmillan, Macmillan Education and Springer Science+Business Media.

SPRINGER NATURE

Change of authorship request form - Journals (pre-acceptance)

Section 6: Declaration of agreement. *All* authors, unchanged, new and removed *must* sign this declaration.
(NB: Please print the form, (docu)-sign and return/upload a scanned copy. Please note that signatures that have been inserted as an image file are acceptable as long as it is handwritten. Typed names in the signature box are unacceptable.) *** Please delete as appropriate. Delete all of the bold if you were on the original authorship list and are remaining as an author.**

	First name	Family name		Signature	Date
1st author	JIE	QIN	I agree to the proposed new authorship shown in section 4 and the proposed change in corresponding author	JIE QIN	2025-6-12
2nd author	QIANYI	GONG	I agree to the proposed new authorship shown in section 4 and the proposed change in corresponding author	QIANYI GONG	2025.6.12
3rd author	CHENG	ZHOU	I agree to the proposed new authorship shown in section 4 and the proposed change in corresponding author	Cheng Zhou	2025-6-12
4th authors	JIETIAN	XU	I agree to the proposed new authorship shown in section 4 and the proposed change in corresponding author	JIE TIAN XU	2025-6-12
5th author	YIFEI	CHENG	I agree to the proposed new authorship shown in section 4 and the proposed change in corresponding author	YIFEI CHENG	2025-6-12
6th author	WEIYUE	XU	I agree to the proposed new authorship shown in section 4 and the proposed change in corresponding author	WEIYUE XU	2025-6-12
7th author	DI	ZHU	I agree to the proposed new authorship shown in section 4 and the proposed change in corresponding author	DI ZHU	2025-6-12

SPRINGER NATURE Springer Nature is one of the world's leading global research, educational and professional publishers, created in May 2015 through the combination of Nature Publishing Group, Palgrave Macmillan, Macmillan Education and Springer Science+Business Media.

SPRINGER NATURE

Change of authorship request form - Journals (pre-acceptance)

	First name	Family name		Signature	Date
8th author	YIMING	LIU	I agree to the proposed new authorship shown in section 4 and the proposed change in corresponding author	YIMING LIU	June 12, 2025
9th author	YUYE	ZHANG	I agree to the proposed new authorship shown in section 4 and the proposed change in corresponding author	YUYE ZHANG	2025-6-12
10th author	YANRU	WANG	I agree to the proposed new authorship shown in section 4 and the proposed change in corresponding author	YANRU WANG	2025-6-12

Please use an additional sheet if there are more than 10 authors.

SPRINGER NATURE Springer Nature is one of the world's leading global research, educational and professional publishers, created in May 2015 through the combination of Nature Publishing Group, Palgrave Macmillan, Macmillan Education and Springer Science+Business Media.

SPRINGER NATURE

Change of authorship request form - Journals (pre-acceptance)

Section 6: Declaration of agreement. *All* authors, unchanged, new and removed *must* sign this declaration.
(NB: Please print the form, (docu)-sign and return/upload a scanned copy. Please note that signatures that have been inserted as an image file are acceptable as long as it is handwritten. Typed names in the signature box are unacceptable.) *** Please delete as appropriate. Delete all of the bold if you were on the original authorship list and are remaining as an author.**

	First name	Family name		Signature	Date
11th author	LINGLING	GAO	I agree to the proposed new authorship shown in section 4 and the proposed change in corresponding author	LINGLING GAO	2025-6-12
12th author	LANFANG	LI	I agree to the proposed new authorship shown in section 4 and the proposed change in corresponding author	LANFANG LI	2025-6-12
13th author	WULEI	HOU	I agree to the proposed new authorship shown in section 4 and the proposed change in corresponding author	WULEI HOU	2025-6-12
14th authors	QIAN	LI	I agree to the proposed new authorship shown in section 4 and the proposed change in corresponding author	QIAN LI	2025.6.12
15th author	BINBIN	LIU	I agree to the proposed new authorship shown in section 4 and the proposed change in corresponding author	BIN BIN LIU	June 12, 2025
16th author	YAZHEN	ZHU	I agree to the proposed new authorship shown in section 4 and the proposed change in corresponding author	YAZHEN ZHU	2025-6-12
17th author	ZUOYUN	WANG	I agree to the proposed new authorship shown in section 4 and the proposed change in corresponding author	Zuoyun Wang	2025-6-12

SPRINGER NATURE Springer Nature is one of the world's leading global research, educational and professional publishers, created in May 2015 through the combination of Nature Publishing Group, Palgrave Macmillan, Macmillan Education and Springer Science+Business Media.

SPRINGER NATURE

Change of authorship request form - Journals (pre-acceptance)

	First name	Family name		Signature	Date
18th author	JIEYI	SHI	I agree to the proposed new authorship shown in section 4 and the proposed change in corresponding author	JIEYI SHI	2025-06-12
19th author	SHUANGJIAN	QIU	I agree to the proposed new authorship shown in section 4 and the proposed change in corresponding author	SHUANGJIAN QIU	June 12, 2025
20th author	CHUNMIN	LIANG	I agree to the proposed new authorship shown in section 4 and the proposed change in corresponding author	CHUNMIN LIANG	06-12-2025

Please use an additional sheet if there are more than 10 authors.

In case of author collaborations with formal agreement:

	Name of consortium/consortia	First name	Family name		Signature	Date
Representative/legal guarantor				I agree to the proposed new authorship shown in section 4 /**and the addition/removal*of my name to the authorship list** /and the proposed change in corresponding author		

Both added/removed authors should complete the information in the first table under Section 6.

---- End of form ----

SPRINGER NATURE Springer Nature is one of the world's leading global research, educational and professional publishers, created in May 2015 through the combination of Nature Publishing Group, Palgrave Macmillan, Macmillan Education and Springer Science+Business Media.
